# One-pot sequential synthesis of tetrasubstituted thiophenes via sulfur ylide-like intermediates

**DOI:** 10.3762/bjoc.14.16

**Published:** 2018-01-26

**Authors:** Jun Ki Kim, Hwan Jung Lim, Kyung Chae Jeong, Seong Jun Park

**Affiliations:** 1 Research Center for Medicinal Chemistry, Korea Research Institute of Chemical Technology (KRICT), 141 Gajeong-ro, Yuseong-gu, Daejeon 34114, Korea; 2 Department of Chemistry, Chungnam National University, 99 Daehak-ro, Yuseong-gu, Daejeon 34134, Korea; 3 Translational Research Branch, National Cancer Center, 323 Ilsan-ro, Ilsandong-gu, Goyang-si Gyeonggi-do 10408, Korea

**Keywords:** 5-(heterocyclic)thiophenes, one-pot sequential synthesis, sulfur ylide, tetrasubstituted thiophene

## Abstract

Herein, we describe a novel approach for the practical synthesis of tetrasubstituted thiophenes **8**. The developed method was particularly used for the facile preparation of thienyl heterocycles **8**. The mechanism for this reaction is based on the formation of a sulfur ylide-like intermediate. It was clearly suggested by (i) the intramolecular cyclization of ketene *N,S*-acetals **7** to the corresponding thiophenes **8**, (ii) ^1^H NMR studies of Meldrum’s acid-substituted aminothioacetals **9**, and (iii) substitution studies of the methoxy group on Meldrum’s acid containing *N,S*-acetals **9b**. Notably, in terms of structural effects on the reactivity and stability of sulfur ylide-like intermediates, 2-pyridyl substituted compound **7a** exhibited superior properties over those of others.

## Introduction

**Figure 1:**
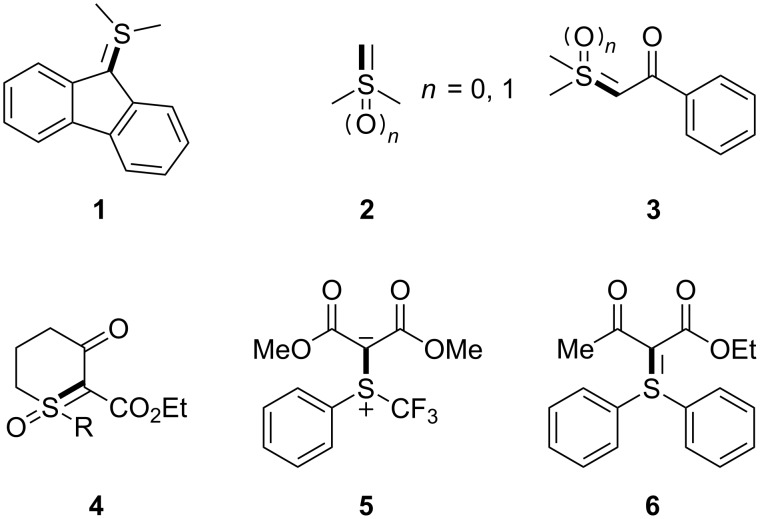
The selected examples of sulfur(IV) and sulfur(VI) ylides **1** [1], **2** [5–7], **3** [6–7,9], **4** [11–12], **5** [33–34], **6** [35–38].

Since the discovery of stable sulfonium ylides **1** in 1930 [1] and the pioneering work of several research groups during the 1960s (**2** and **3**) [2–9], these carbene precursors have been played an important role in organic chemistry [10–22]. As shown in Figure 1, sulfur(IV) and sulfur(VI) ylides are stable. The stability of sulfonium ylides is determined by the electron delocalization of the carbanionic center and the substituents on the sulfur atom [10]. In general, these reagents are often applied in the preparation of simple small rings [13], such as epoxides [14–18], cyclopropanes [19–22], aziridines [23], indoles [24], pyrroles [24], and indolines [25]. In addition, other reactions involving sulfonium and sulfoxonium ylides have been reported recently [26–32]. For example, Shen and co-workers reported the use of trifluoromethyl-substituted sulfonium ylide **5** in electrophilic trifluoromethylation reactions [33–34]. Moreover, Maulide and co-workers reported an effective ylide transfer reagent, which led to sulfonium ylide **6** [35–38].

As part of our ongoing efforts to discover small molecule modulators of protein–protein interactions (PPIs), we are particularly interested in coplanar compounds that mimic β-strand side-chain distributions [39–43]. Consequently, we are fascinated with thienyl–pyridyl ring systems [43] and have explored facile synthetic procedures to facilitate their production. For the synthesis of heterocyclic–heterocyclic biaryl compounds, numerous studies have been carried out to develop efficient catalytic methods [44–49]. In general, Pd-catalyzed Suzuki–Miyaura cross-coupling reactions are the most popular synthetic strategy for aryl–aryl bond-forming reactions [50–52]. However, it has been reported that the Suzuki cross-coupling of nitrogen- and sulfur-containing heterocycles is more challenging than those of aryl–aryl derivatives. These difficulties resulted from the special properties of thiopheneboronic acids – the sensitivity to polar reaction media and easy degradation by protodeboronation [53].

**Figure 2:**
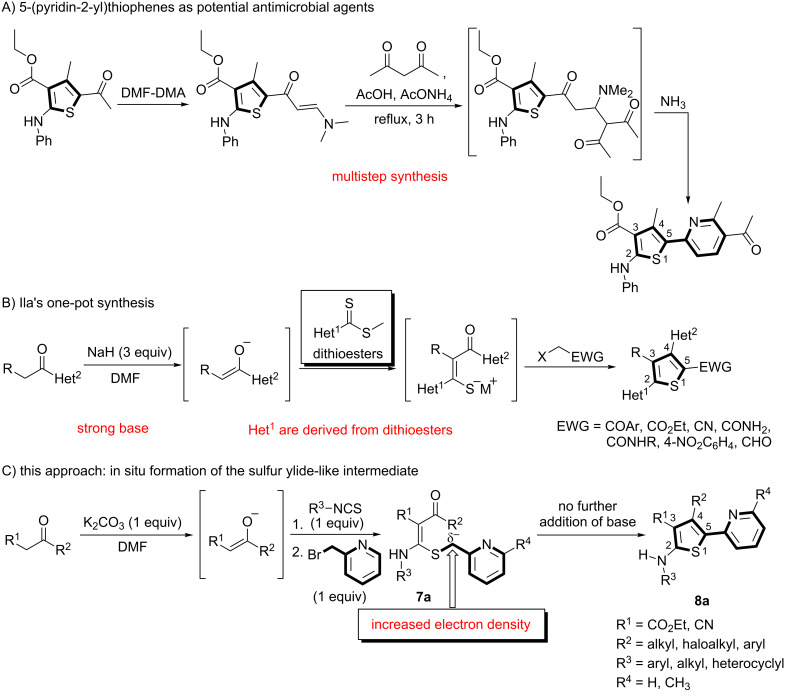
Metal-free synthesis of thiophene-based heterocycles (A) [54–55], (B) [56].

As a recent example of a metal-free synthesis of the targeted thienylpyridines (Figure 2A and 2B), Al-Showiman and co-workers reported a trisubstituted 5-(pyridin-2-yl)thiophene, obtained from the reaction of 5-(enaminone)thiophene with 2,4-pentanedione in glacial acetic acid in the presence of ammonium acetate [54–55]. Ila and co-workers reported the synthesis of tri- and tetrasubstituted thiophenes via the intramolecular cyclization of *S*-alkylated heterocyclic–aryl dithioesters [56]. However, these approaches are limited by the multistep synthesis (Figure 2A) [54–55] and the complicated dithioester preparation (Figure 2B) [56]. In general, tetrasubstituted thiophenes have primarily been prepared by base-catalyzed intramolecular Dieckmann-, Thorpe–Ziegler, and aldol-type condensations of the corresponding ketene-*N,S*-acetals [57–67]. These methods are still need strong bases [60], high temperatures [62,64–65], and are generally low yielding [57,62]. Thus, a new mild synthetic route for the synthesis of 5-(pyridyl)thiophenes is required. We therefore investigated the synthesis of thienylpyridines using a metal-free approach.

## Results and Discussion

**Scheme 1:**
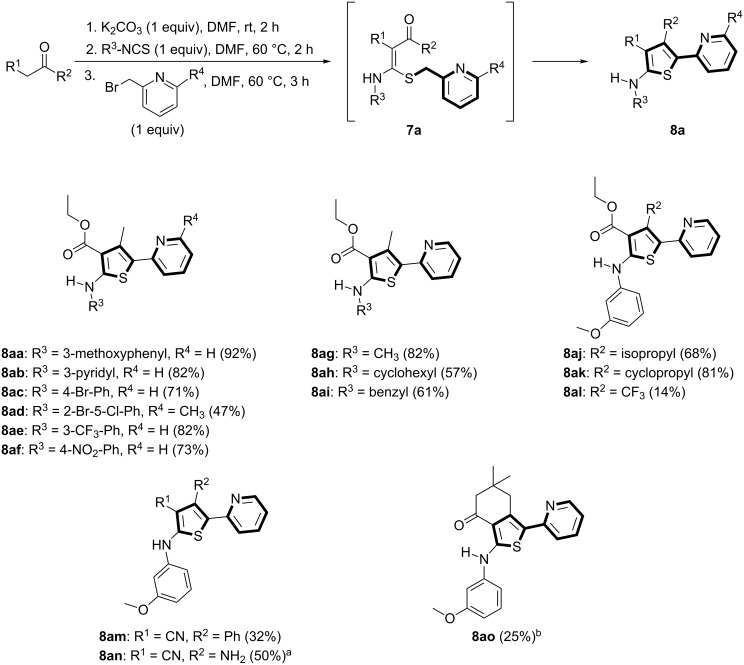
One-pot sequential synthesis of the trisubstituted 5-(pyridine-2-yl)thiophenes **8a**. Substrate: ^a^malonitrile; ^b^5,5-dimethylcyclohexane-1,3-dione.

At first, our efforts focused on the intramolecular cyclization reactions with mild conditions – in the absence of an added base at room temperature. To obtain aminothioacetal **7a**, we initially performed the *S*-alkylation of the intermediate thiolate salt with 2-(bromomethyl)pyridine at room temperature overnight. We interestingly found that the desired 5-(pyridin-2-yl)thiophenes **8a** has already been achieved by the intramolecular aldol-type condensation of *N,S*-acetal **7a** (Figure 2C). Subsequently, we investigated the scope of the reaction using our optimized conditions (Scheme 1).

**Figure 3:**
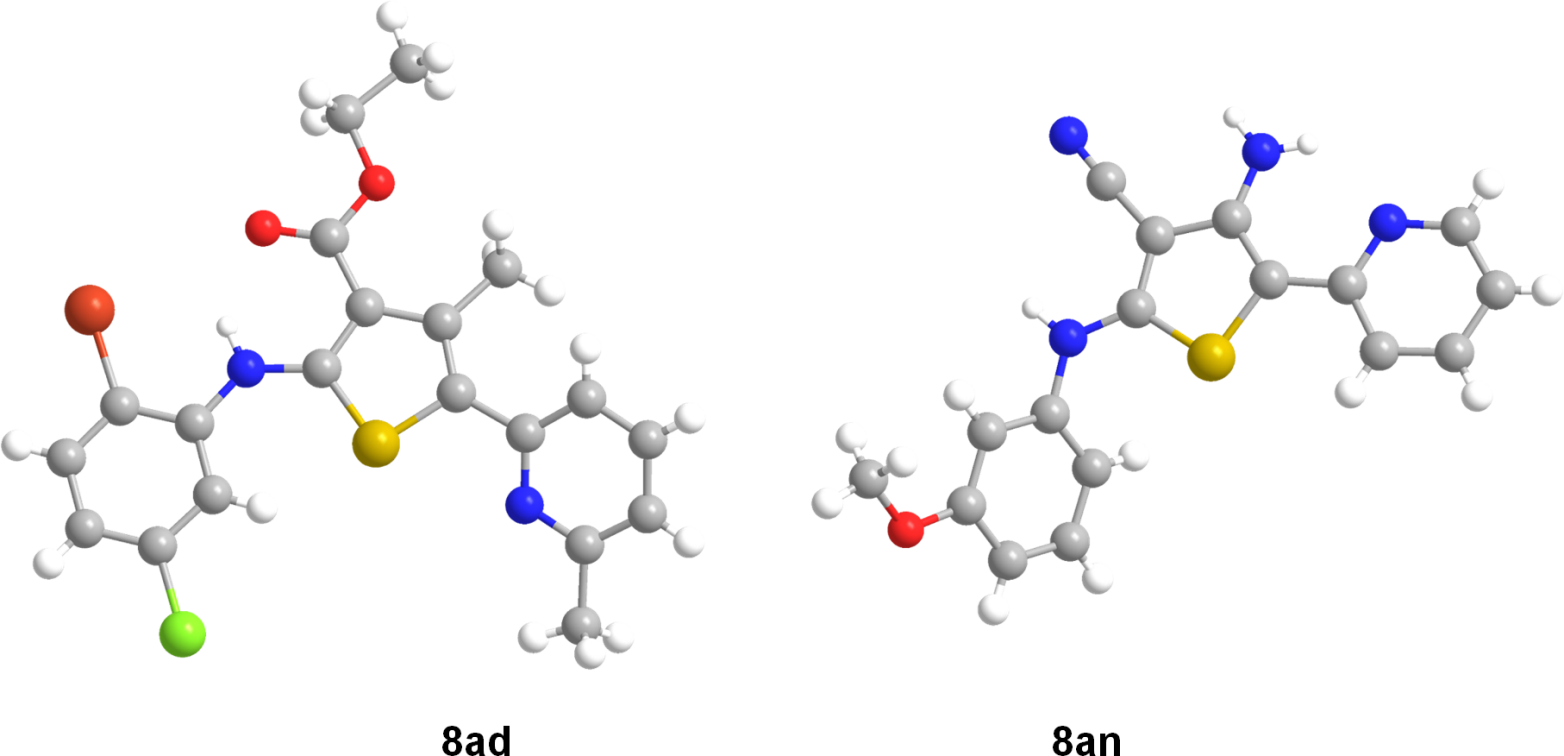
X-ray crystal structures of **8ad** and **8an** [68].

As shown in Scheme 1, various isothiocyanates containing aryl and alkyl groups were applied, and the desired thiophenes (**8aa–ai**) were obtained in moderate to excellent yields (47–92%). When different 1,3-diketones were applied, the yields were affected by the keto–enol tautomer ratio. Alkyl substituents (isopropyl and cyclopropyl), which promote the enol forms of the ketones, afforded thiophenes **8aj** and **8ak** in good to excellent yields (68% and 81%). However, a CF_3_ substituent, which is electron-withdrawing and might promote the keto form, provided the desired compound **8al** in a low yield (14%). When the enolate was derived from 3-oxo-3-phenylpropanenitrile, 3-cyano-4-phenylthiophene **8am** was obtained in a low yield (32%). Starting from malonitrile, compound **8an** was also prepared in a moderate yield (50%) via a Thorpe–Ziegler-type cyclization of *N,S*-acetal **7an**. In this case, the intramolecular cyclization reaction was carried out at 100 °C for 3 h. With 5,5-dimethylcyclohexane-1,3-dione, thiophene **8ao** was obtained in a low yield (25%). X-ray crystal structures of thiophenes **8ad** and **8an** are illustrated in Figure 3 [68].

Mechanistically, our experimental findings may be attributed to the formation of sulfur ylide-like intermediates. To support this reaction pathway, further studies were performed. By changing the substituent groups on *N,S*-acetals **7**, the effects of the structure on the stability and the reactivity of the intermediates were investigated (Table 1).

**Table 1:** Examination of *N*,*S*-acetals substituted with a heterocycle (**7aa–k**) or an arene (**7l–p**).

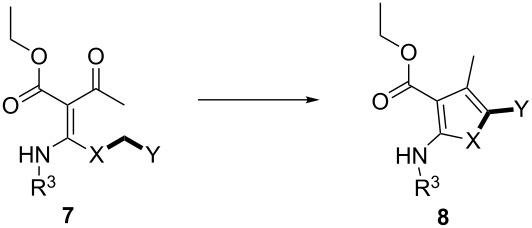					

Entry	Substrates	X	Y	Products	Yield (%)^a,b^

1	**7aa**	S	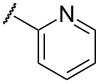	**8aa**	92
2	**7b**	S	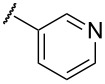	**8b**	–^c^
3^d^	**7c**	S	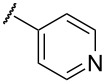	**8c**	80
4	**7d**	S	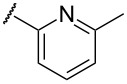	**8d**	34
5	**7e**	O	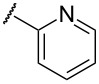	**8e**	–^c^

6	**7f**	S	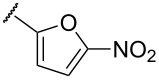	**8f**	33
7	**7g**	S	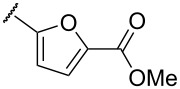	**8g**	20
8	**7h**	S	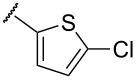	**8h**	–^c^
9	**7i**	S	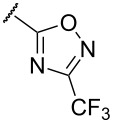	**8i**	47
10	**7j**	S	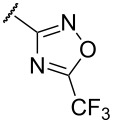	**8j**	–^c^
11	**7k**	S	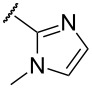	**8k**	8

12	**7l**	S	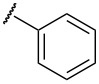	**8l**	–^c^
13	**7m**	S	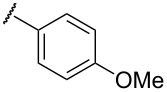	**8m**	–^c^
14	**7n**	S	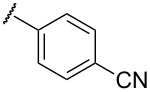	**8n**	–^c^
15	**7o**	S	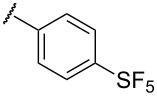	**8o**	–^c^
16	**7p**	S	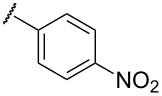	**8p**	42

^a^R^3^ = 3-methoxyphenyl, one-pot sequential reactions to thiophenes **8**: a) ethyl acetoacetate (1 equiv), K_2_CO_3_ (1 equiv), DMF, rt, 2 h; b) 3-methoxyphenyl isothiocyanate (1 equiv), DMF, 60 °C, 2 h; c) the corresponding halomethylarenes or halomethyl heterocycles (1 equiv), DMF, 60 °C, 3 h; ^b^After column chromatography; ^c^no desired reaction; ^d^because *N,S*-acetal **7c** readily transfer to the corresponding thiophene **8c** at 0 °C, the substrate **7c** could not be isolated.

In terms of inductive and mesomeric effects, we postulated that the electron rich pyridyl N atom could carry a negative charge at the picolinyl position (Table 1, entries 1 to 5). Interestingly, the 2-pyridyl moiety provided stable and reactive *N,S*-acetal **7aa**, which could be isolated and afforded the desired thiophene **8aa** in an excellent yield (92%, Table 1, entry 1). The substrate containing a 3-pyridyl group only afforded *S*-alkylated compound **7b**, while 4-pyridyl substituted intermediate **7c** could be easily transformed into thiophene **8c** at 0 °C (Table 1, entries 2 and 3). Notably, the special properties associated with the 2- and 4-positions of pyridine [69–72] are evident in this study. In the case of 6-methylpyridine-substituted *N,S*-acetal **7d**, the formation of a resonance stabilized enaminate anion had a smaller contribution and this resulted in a reduced yield (34%, Table 1, entry 4) [70]. To identify the effects of sulfur, a reaction with the corresponding isocyanate was performed to introduce an oxygen atom. As a result, only *O*-alkylation compound **7e** was obtained instead of the desired furan (Table 1, entry 5). It is possible to consider that the d orbitals of the sulfur atom in a sulfide group could possibly stabilize the adjacent carbanion [73–74].

To expand the scope of substituted *N*,*S*-acetals that could provide the desired sulfur ylide-like intermediates, various heterocycles were subjected to the reaction (Table 1, entries 6–11). The desired thiophenes **8f** and **8g** were obtained in low yields from the respective furans (33% and 20%, Table 1, entries 6 and 7). With thiophene, however, only *N,S*-acetal compound **7h** was obtained. Thiophene could not generate the desired intermediate because of the lower electronegativity and a weaker inductive effect of sulfur (Table 1, entry 8). Among 1,2,4-oxadiazole moieties, the 3-trifluoromethyloxadiazole group afforded the desired thiophene **8i** (Table 1, entry 9), whereas the 5-trifluoromethyloxadiazole substituent was not a viable substrate (Table 1, entry 10). Because of a similar result obtained with the *N*-methylimidazole substituted compound **7k**, the difference between **7i** and **7j** could be explained by the imidazole-like structure of the 5-trifluoromethyloxadiazole moiety. The reduced inductive effect of the amine might be attributed to the resonance structures of imidazole (Table 1, entry 11) [72].

To determine the influence of substituents on the phenyl group, various arene(methyl)sulfanes **7l–p** were tested (Table 1, entries 12–16,). Simple phenyl and electron-donating compounds **7l** and **7m** did not provide the desired thiophenes **8l** and **8m**. Although electron-withdrawing groups such as CN and SF_5_ did not show any effect (Table 1, entries 14 and 15), NO_2_, the strongest electron-withdrawing group [75–77], provided the desired thiophene **8p** in a moderate yield (42%).

**Figure 4:**
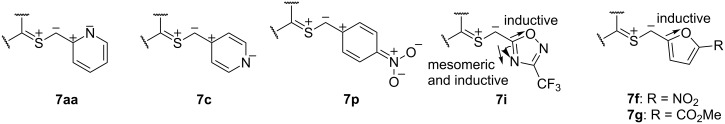
The proposed structure of sulfur ylide-like intermediates; resonance contributors (mesomeric structures) [69–72,78].

While further studies are required, we suggest the sulfur ylide-like intermediates **7aa**, **7c**, **7p**, **7i**, and **7f**,**g** after considering the literature [69–72] and McNab’s research on the synthesis of 3-hydroxythiophene and thiphene-3(2*H*)-ones (Figure 4) [78]. With regards to McNab’s work, the dipolar species [R_2_C=S^+^−CH^−^−R′] were proposed as reaction intermediates [78]. In our studies, it was shown that the order of reactivity was **7c** ≥ **7aa** > **7i**, **7p** ≥ **7f**, **7g**. The different reactivities of the intermediates were related to the presence of heteroatoms, particularly their inductive and mesomeric effects [69–72]. For example, 2-pyridyl-substituted ylide-like intermediate **7aa** showed the desired properties in terms of both reactivity and stability, whereas the 4-pyridyl group only displayed high reactivity. For alkylpyridines **7aa** and **7c**, our observations may be explained by Fraser’s measurements of the p*K*a values [69–70,79]: among isomeric benzylpyridines, the 4-isomer is more acidic than the 2-isomer, and the 4- and 2-isomers are much more acidic than the 3-isomer. In the case of the oxadiazole-substituted compound **7i**, inductive and mesomeric effects facilitated its sulfur ylide-like intermediate formation [71]. For compounds **7f** and **7g**, the low reactivity resulted from the decreased mesomeric effect of the furan structure: the higher electronegativity of oxygen facilitated the polarized form [71]. Among various arenes, the 4-nitrophenyl substituent **7p** only afforded the desired thiophene **8p** in a moderated yield (42%) and, the favorable resonance form is illustrated in Figure 4.

According to the recent reports on the multiple isomeric structures of ketene *N,S*-acetals [80–83], structural assignments of the ketene *N,S*-aminothioacetals **7** by ^1^H NMR are not facile. To overcome these difficulties, we prepared *N,S*-acetals **9a**–**c** since the X-ray crystal structure of Meldrum’s acid-based *N,S*-acetal was reported by Wentrup [84]. In addition, the intramolecular aldol condensation of Meldrum’s acids did not occur due to the ketone structures. Table 2 displays the ^1^H NMR result of the sulfur ylide-like intermediate **9b**, and demonstrates the effect of increasing electronegativity on the CH_2_ proton. The 2-pyridyl group caused a downfield shift of 0.13 to 0.14 ppm compared to phenyl and 3-pyridyl groups (Table 2, entry 2).

**Table 2:** ^1^H NMR studies of Meldrum’s acid-based *N,S*-acetals **9a–c**^a,b^ [84].

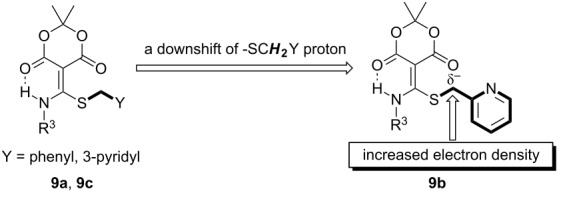			

Entry	*N,S*-Acetals^c,d^	Structure	-SC**H_2_**Y ^1^H NMR (ppm)^e^

1	**9a**	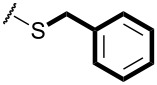	4.02
2	**9b**	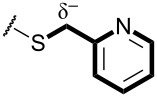	4.15
3	**9c**	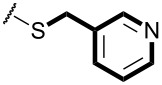	4.01

^a^R^3^ = 3-methoxyphenyl; ^b^*S*-alkylation of the thiolate with 4-(bromomethyl)pyridine hydrobromide was not successful; ^c^one-pot sequential reactions to *N,S*-acetals **9**: a) Meldrum’s acid (1 equiv), K_2_CO_3_ (1 equiv), DMF, rt, 2 h; b) 3-methoxyphenyl isothiocyanate (1 equiv), DMF, 60 °C, 2 h; c) the corresponding benzyl bromide or bromomethylpyridine (1 equiv), DMF, 60 °C, 3 h; ^d^After column chromatography; ^e^in CDCl_3_.

Further ^1^H NMR studies of pyridin-2-ylmalononitrile **7an**, pyridine-2-ylmethyl methanimidothioate **7ao**, and time dependent experiments of the intramolecular aldol condensation of *N,S*-acetal **7aa** to **8aa** in *N,N*-dimethylformamide-*d*_7_ at room temperature confirmed the formation of the stable sulfur ylide-like intermediates, thus indicating the successful transformation into thiophenes **8an**, **8ao**, and **8a** (see Supporting Information File 1).

**Scheme 2:**
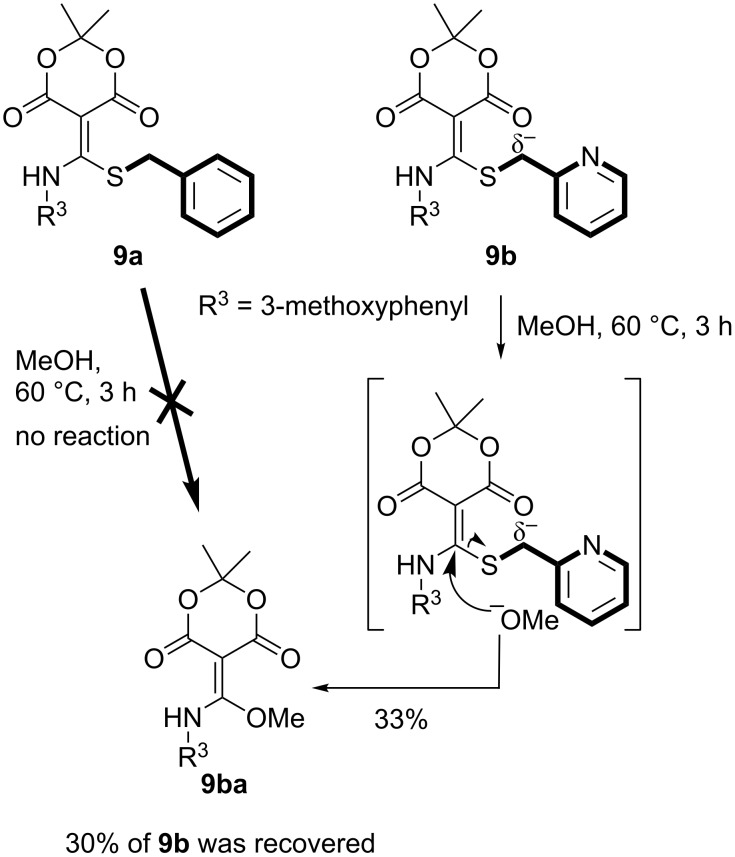
The substitution reaction with MeOH.

In addition to the spectroscopic studies, we attempted to gain additional evidence to support the formation of sulfur ylide-like intermediates via another approach. We selected stable Meldrum’s acid containing *N,S*-acetals **9a** and **9b** for further investigation. Based on previous reports regarding carbene generation from sulfonium ylides [6,85–86], compounds **9a** and **9b** were reacted with excess MeOH (Scheme 2). Interestingly, 2-pyridyl-substituted *N,S*-acetal **9b** only provided *N,O*-acetal **9ba** via a 1,4-Micheal addition, whereas *N,S*-acetal **9a** was completely recovered after the reaction. We believed that these results support the existence of sulfur ylide-like intermediates (Scheme 2) [87].

## Conclusion

In conclusion, we have developed a new synthetic pathway for the preparation of 2-amino-5-(heterocyclic)thiophenes **8**. We have also shown that sulfur ylide-like intermediates **7**, which are easily converted into the desired thiophenes **8**, can be generated in situ by *S*-alkylation of the intermediate thiolate salts. By ^1^H NMR analysis of *N,S*-acetals **9** and methoxy group substitution of **9b**, the formation of sulfur ylide-like intermediates was successfully demonstrated. The transformation of ylide-like intermediates into the corresponding thiophenes was affected by their electronic properties. Among the various tested residues, the 2-pyridyl motif provided the desired reactivity and stability. This approach could be considered a powerful strategy for the preparation of biologically important thienyl heterocycles. Subsequent studies shall focus on applying this chemistry in other reactions that require sulfur ylides, and the biological activities of thiophenes **8** will also be reported in due course.

## Supporting Information

File 1 Experimental part.
