# Supplementary material for: One-pot sequential synthesis of tetrasubstituted thiophenes via sulfur ylide-like intermediates
Source: Beilstein J Org Chem. 2018 Jan 26;14:243–52. doi: 10.3762/bjoc.14.16 (PMC5789428; doi:10.3762/bjoc.14.16)
Supplement: File 1 — Experimental part. [file Beilstein_J_Org_Chem-14-243-s001.pdf]

Supporting Information

for

One-pot sequential synthesis of tetrasubstituted thiophenes via sulfur ylide-like intermediates

Jun Ki Kim[1,3], Hwan Jung Lim[1], Kyung Chae Jeong[2,*] and Seong Jun Park[1,*]

Address: [1]Research Center for Medicinal Chemistry, Korea Research Institute of Chemical Technology (KRICT), 141 Gajeong-ro, Yuseong-gu, Daejeon 34114, Korea, [2]Translational Research Branch, National Cancer Center, 323 Ilsan-ro, Ilsandong-gu, Goyang-si Gyeonggi-do 10408, Korea and [3]Department of Chemistry, Chungnam National University, 99 Daehak-ro, Yuseong-gu, Daejeon 34134, Korea

Email: Email: Kyung Chae Jeong - jeongkc@ncc.re.kr; Seong Jun Park - sjunpark@krict.re.kr

* Corresponding author

Experimental part

Contents :

1. Experimental S2
2. ^{1}H and ^{13}C NMR of compound **8aa** S23
3. ^{1}H NMR Studies of *N,S*-acetals **7an** and **7ao** S50
4. The time dependent ^{1}H NMR studies of the intramolecular aldol condensation of sulfur ylide-like intermediates **7aa** to **8aa** in *N,N*-dimethylformamide-d_7 at room temperature S51
5. X-ray data of **8ad** S52
6. X-ray data of **8an** S62

Experimental

General information

Analytical thin layer chromatography (TLC) was performed on Kieselgel 60 F_{254} glass plates precoated with a 0.2 mm thickness of silica gel. The TLC plates were visualized by shortwave (254 nm), potassium permanganate or ceric ammonium molybdate stain. Flash chromatography was carried out with Kieselgel 60 (230−400 mesh) silica gel. Melting points: Barnstead/Elecrothermal 9300, measurements were performed in open glass capillaries. IR spetra: Bruker ALPHA-P & ALPHA-T. NMR spectra: Bruker AV 300MHz (^{1}H NMR: 300 MHz, ^{13}C NMR: 75 MHz), AV 500 MHz (^{1}H NMR: 500 MHz, ^{13}C NMR: 125 MHz), AV2 500 MHz (^{19}F NMR: 470 MHz), the spectra were recorded in $CDCl_3$ and DMSO-d_6 using TMS as internal standard and are reported in ppm. ^{1}H NMR data are reported as: (s = singlet, d = doublet, t = triplet, q = quartet, br = broad singlet, qui = quintet, oct = octet, m = multiplet; coupling constant(s) in Hz; integration, proton assignment). High-resolution mass spectra (HRMS): JEOL JMS-700. All solvents were purified using column filter solvent purification system before use unless otherwise indicated. Reagents were purchased and used without further purification.

Ethyl 2-((3-methoxyphenyl)amino)-4-methyl-5-(pyridin-2-yl)thiophene-3-carboxylate (8aa)

To a stirred solution of potassium carbonate (102 mg, 0.740 mmol) in DMF (0.5 mL) was added ethyl acetoacetate (0.09 mL, 0.740 mmol). After stirring at rt for 2 h, 3-methoxyphenyl isothiocyanate (0.10 mL, 0.740 mmol) was added dropwise at 0 °C. Then, the mixture was stirred at 60 °C for 2 h before addition of 2-(bromomethyl)pyridine hydrobromide (187 mg, 0.740 mmol). The reaction mixture was stirred at 60 °C for 3 h and extracted with ethyl acetate. The organic layer was dried over anhydrous Na_2SO_4, filtered, and evaporated. The resulting crude residue was purified by column chromatography (ethyl acetate/Hex, 5%) on silica gel to give the thiophene **8aa** (250 mg, 92% yield) as a brown solid.

mp. 88 - 89 °C; ^{1}H NMR (300 MHz, $CDCl_3$) δ 10.48 (s, 1H), 8.56 – 8.59 (m, 1H), 7.67 (td, *J* = 1.8 Hz, *J* = 7.8 Hz, 1H), 7.48 (d, *J* = 8.1 Hz, 1H), 7.26 (dd, *J* = 7.2 Hz, *J* = 9.1 Hz, 1H), 7.07 – 7.12 (m, 1H), 7.00 (dd, *J* = 1.8 Hz, *J* = 8.0 Hz, 1H), 6.89 (t, *J* = 2.2 Hz, 1H), 6.62 (dd, *J* = 2.1 Hz, *J* = 8.1 Hz, 1H), 4.37 (q, *J* = 7.1 Hz, 2H), 3.81 (s, 3H), 2.59 (s, 3H), 1.42 (t, *J* = 7.1 Hz, 3H). ^{13}C NMR (126 MHz, $CDCl_3$) δ 167.1, 160.6, 160.4, 153.1, 149.5, 141.7, 136.2, 133.9, 130.2, 122.3, 121.3, 120.7, 111.6, 109.1, 109.0, 105.1, 60.2, 55.4, 16.7, 14.4; HRMS (EI) calcd for $C_{20}H_{20}N_2O_3S$ 368.1195, found 368.1197.

Ethyl 4-methyl-5-(pyridin-2-yl)-2-(pyridin-3-ylamino)thiophene-3-carboxylate (8ab)

To a stirred solution of potassium carbonate (110 mg, 0.796 mmol) in DMF (0.5 mL) was added ethyl acetoacetate (0.10 mL, 0.796 mmol). After stirring at rt for 2 h, a solution of 3-pyridyl isothiocyanate (0.09 mL, 0.796 mmol) in DMF (1 mL) was added

dropwise at 0 °C. Then, the mixture was stirred at 60 °C for 2 h before addition of 2-(bromomethyl)pyridine hydrobromide (201 mg, 0.796 mmol). The reaction mixture was stirred at 60 °C for 3 h and extracted with ethyl acetate. The organic layer was dried over anhydrous Na_2SO_4, filtered and evaporated. The resulting crude residue was purified by column chromatography (ethyl acetate/Hex, 30%) on silica gel to give the thiophene **8ab** (221 mg, 82% yield) as a brown solid.

mp. 113 - 114 °C; ^{1}H NMR (300 MHz, $CDCl_3$) δ 10.54 (s, 1H), 8.57-8.60 (m, 2H), 8.31 (dd, *J* = 1.3 Hz, *J* = 4.7 Hz, 1H), 7.83 (ddd, *J* = 1.3 Hz, *J* = 2.9 Hz, *J* = 8.3 Hz, 1H), 7.69 (td, *J* = 1.8 Hz, *J* = 7.9 Hz, 1H), 7.51 (d, *J* = 8.0 Hz, 1H), 7.28-7.31 (m, 1H), 7.13 (ddd, *J* = 1.0 Hz, *J* = 4.9 Hz, *J* = 7.5 Hz, 1H), 4.39 (q, *J* = 7.1 Hz, 2H), 2.61 (s, 3H), 1.42 (t, *J* = 7.1, 3H); ^{13} C NMR (125 MHz, $CDCl_3$) δ 167.0, 159.5, 152.8, 149.5, 144.1, 141.8, 137.2, 136.3, 133.9, 125.0, 123.8, 122.2, 122.1, 120.9, 110.3, 60.4, 16.6, 14.4; HRMS (EI) calcd for $C_{18}H_{17}N_3O_2S$ 339.1041, found 339.1045.

Ethyl 2-((4-bromophenyl)amino)-4-methyl-5-(pyridin-2-yl)thiophene-3-carboxylate (8ac)

To a stirred solution of potassium carbonate (64 mg, 0.467 mmol) in DMF (0.5 mL) was added ethyl acetoacetate (0.06 mL, 0.4671mmol). After stirring at rt for 2 h, 4-bromophenyl isothiocyanate (100 mg, 0.4671mmol) was added dropwise at 0 °C. Then, the mixture was stirred at 60 °C for 2 h before addition of 2-(bromomethyl)pyridine hydrobromide (118 mg, 0.4671mmol). The reaction mixture was stirred at 60 °C for 3 h and extracted with ethyl acetate. The organic layer was dried over anhydrous Na_2SO_4, filtered and evaporated. The resulting crude residue was purified by column chromatography (ethyl acetate/Hex, 5%) on silica gel to give the thiophene **8ac** (138 mg, 71% yield) a yellow solid.

mp. 115 - 116 °C; ^{1}H NMR (300 MHz, $CDCl_3$) δ 10.50 (s, 1H), 8.59 (d, *J* = 4.7 Hz, 1H), 7.69 (td, *J* = 1.5 Hz, *J* = 7.9 Hz, 1H), 7.42-7.52 (m, 3H), 7.27 (d, *J* = 4.0 Hz, 2H), 7.12 (dd, *J* = 5.4 Hz, *J* = 6.9 Hz, 1H), 4.38 (q, *J* = 7.1 Hz, 2H), 2.60 (s, 3H), 1.42 (t, *J* = 7.1, 3H); ^{13} C NMR (125 MHz, $CDCl_3$) δ 167.0, 159.8, 153.0, 149.5, 139.6, 136.3, 133.9, 132.4, 122.2, 121.6, 120.8, 120.7, 115.6, 109.6, 60.3, 16.7, 14.4; HRMS (EI) calcd for $C_{19}H_{17}BrN_2O_2S$ 418.0175, found 416.0179.

Ethyl 2-((2-bromo-5-chlorophenyl)amino)-4-methyl-5-(6-methylpyridin-2-yl)thiophene-3-carboxylate (8ad)

To a stirred solution of potassium carbonate (74 mg, 0.537 mmol) in DMF (0.5 mL) was added ethyl acetoacetate (0.07 mL, 0.537 mmol). After stirring at rt for 2 h, 2-bromo-5-chlorophenyl isothiocyanate (134 mg, 0.537 mmol) was added dropwise at 0 °C. Then, the mixture was stirred at 60 °C for 2 h before addition of 2-(bromomethyl)-6-methylpyridine (100 mg, 0.537 mmol). The reaction mixture was stirred at 60 °C for 3 h and extracted with ethyl acetate. The organic layer was dried over anhydrous Na_2SO_4, filtered and evaporated. The resulting crude residue was purified by column chromatography (ethyl acetate/Hex, 5%) on silica gel to give the thiophene **8ad** (118 mg, 47 % yield) a yellow solid

mp. 117 - 118 °C; ^{1}H NMR (300 MHz, CDCl3) δ 10.64 (s, 1H), 7.81 (s, 1H), 7.60 (t, *J* = 7.8 Hz, 1H), 7.51 (d, *J* = 8.5 Hz, 1H), 7.31 (d, *J* = 7.8 Hz, 1H), 7.02 (d, *J* = 7.5 Hz, 1H), 6.88 (d, *J* = 6.6 Hz, 1H), 4.41 (q, *J* = 7.0 Hz, 2H), 2.59 (s, 3H), 2.58 (s, 3H), 1.42 (t, *J* = 7.1 Hz, 3H). ^{13}C NMR (126 MHz, CDCl3) δ 166.4, 158.5, 156.9, 151.9, 139.8, 136.6, 134.1, 133.8, 133.7, 123.2, 120.8, 119.8, 117.6, 111.75, 111.67, 60.5, 24.5, 16.5, 14.4; HRMS (EI) calcd for $C_{20}H_{18}BrClN_2O_2S$ 463.9961, found 463.9949.

Ethyl 4-methyl-5-(pyridin-2-yl)-2-((3-(trifluoromethyl)phenyl)amino)thiophene-3-carboxylate (8ae)

To a stirred solution of potassium carbonate (68 mg, 0.492 mmol) in DMF (0.5 mL) was added ethyl acetoacetate (0.06 mL, 0.492 mmol). After stirring at rt for 2 h, 3-(trifluoromethyl)phenyl isothiocyanate (0.07 mL, 0.492 mmol) was added dropwise at 0 °C. Then, the mixture was stirred at 60 °C for 2 h before addition of 2-(bromomethyl)pyridine hydrobromide (124 mg, 0.492 mmol). The reaction mixture was stirred at 60 °C for 3 h and extracted with ethyl acetate. The organic layer was dried over anhydrous Na_2SO_4 filtered and evaporated. The resulting crude residue was purified by column chromatography (ethyl acetate/Hex, 5%) on silica gel to give the thiophene **8ae** (123 mg, 82% yield) a yellow solid.

mp. 120 - 121 °C; ^{1}H NMR (300 MHz, $CDCl_3$) δ 10.68 (s, 1H), 8.60 (d, *J* = 4.7 Hz, 1H), 7.70 (td, *J* = 1.7 Hz, *J* = 7.8 Hz, 1H), 7.62 (d, *J* = 8.2 Hz, 1H), 7.43-7.54 (m, 3H), 7.30 (d, *J* = 7.7 Hz, 1H), 7.14 (dd, *J* = 4.9 Hz, *J* = 7.4 Hz, 1H), 4.40 (q, *J* = 7.1 Hz, 2H), 2.61 (s, 3H), 1.43 (t, *J* = 7.1 Hz, 3H); ^{13} C NMR (125 MHz, $CDCl_3$) δ 167.1, 159.2, 152.9, 149.6, 141.0, 136.3, 133.9, 132.0 (d, *J* = 32.3 Hz), 130.1, 122.4, 122.1, 121.7 (q, *J* = 271 Hz, CF_3), 121.5, 121.0, 119.4 (d, *J* = 3.3 Hz), 115.9 (d, *J* = 3.4 Hz), 110.1, 60.4, 16.6, 14.3; HRMS (EI) calcd for $C_{20}H_{17}F_3N_2O_2S$ 406.0963, found 406.0957.

Ethyl 4-methyl-2-((4-nitrophenyl)amino)-5-(pyridin-2-yl)thiophene-3-carboxylate (8af)

To a stirred solution of potassium carbonate (180 mg, 0.555 mmol) in DMF (0.5 mL) was added ethyl acetoacetate (0.07 mL, 0.555 mmol). After stirring at rt for 2 h, 4-nitrophenyl isothiocyanate (0.07 mL, 0.555 mmol) was added dropwise at 0 °C.

Then, the mixture was stirred at 60 °C for 2 h before addition of 2-(bromomethyl)pyridine hydrobromide (140 mg, 0.555 mmol). The reaction mixture was stirred at 60 °C for 3 h and extracted with ethyl acetate. The organic layer was dried over anhydrous Na_2SO_4 filtered and evaporated. The resulting crude residue was purified by column chromatography (MC/MeOH 2%) to give the thiophene **8af** (156 mg, 73% yield) an orange solid.

mp. 182 - 183 °C; ^{1}H NMR (300 MHz, $CDCl_3$) δ 11.18 (s, 1H), 8.63 (d, *J* = 4.5 Hz, 1H), 8.24 (d, *J* = 9.1 Hz, 2H), 7.74 (td, *J* = 1.7 Hz, *J* = 8.0 Hz, 1H), 7.55 (d, *J* = 8.1. Hz, 1H), 7.41 (d, *J* = 9.1 Hz, 2H), 7.19 (dd, *J* = 4.9 Hz, *J* = 7.4 Hz, 1H), 4.42 (q, *J* = 7.1 Hz, 2H), 2.62 (s, 3H), 1.44 (t, *J* = 7.1, 3H); ^{13} C NMR (125 MHz, $CDCl_3$) δ 167.0, 156.1, 152.5, 149.7, 145.8, 141.6, 136.5, 133.7, 125.9, 124.2, 122.4, 121.4, 116.7, 112.3, 60.8, 16.6, 14.3; HRMS (EI) calcd for $C_{19}H_{17}N_3O_4S$ 383.0940, found 383.0939.

Ethyl 4-methyl-2-(methylamino)-5-(pyridin-2-yl)thiophene-3-carboxylate (8ag)

To a stirred solution of potassium carbonate (220 mg, 1.592 mmol) in DMF (0.5 mL) was added ethyl acetoacetate (0.20 mL, 1.592mmol). After stirring at rt for 2 h, a solution of methyl isothiocyanate (0.20 mL, 1.592 mmol) in DMF (1 mL) was added dropwise at 0 °C. Then, the mixture was stirred at 60 °C for 2 h before addition of 2-(bromomethyl)pyridine hydrobromide (402 mg, 1.592 mmol). The reaction mixture was stirred at 60 °C for 3h and extracted with ethyl acetate. The organic layer was dried over anhydrous Na_2SO_4, filtered and evaporated. The resulting crude residue was purified by column chromatography (ethyl acetate/Hex, 15%) on silica gel to give the thiophene **8ag** (149 mg, 82% yield) a yellow solid.

mp. 110 - 111 °C; ^{1}H NMR (300 MHz, $CDCl_3$) δ 8.55 (d, *J* = 4.2 Hz, 1H), 7.94 (d, *J* = 3.8 Hz, 1H), 7.63 (td, *J* = 1.7 Hz, *J* = 7.8 Hz, 1H), 7.46 (d, *J* = 8.0 Hz, 1H), 7.02-7.06 (m, 1H), 4.30 (q, *J* = 7.1 Hz, 2H), 3.04(d, *J* = 5.1 Hz, 3H), 2.57 (s, 3H), 1.37 (t, *J* = 7.1, 3H); ^{13} C NMR (125 MHz, $CDCl_3$) δ 168.3, 166.8, 153.5, 149.3, 136.1, 135.4, 121.6, 120.2, 120.1, 105.4, 59.6, 33.1, 16.7, 14.5; HRMS (EI) calcd for $C_{14}H_{16}N_2O_2S$ 276.0932, found 276.0925.

Ethyl 2-(cyclohexylamino)-4-methyl-5-(pyridin-2-yl)thiophene-3-carboxylate (8ah)

To a stirred solution of potassium carbonate (98 mg, 0.708 mmol) in DMF (0.5 mL) was added ethyl acetoacetate (0.09 mL, 0.708 mmol). After stirring at rt for 2 h, cyclohexyl isothiocyanate (0.10 mL, 0.708 mmol) was added dropwise at 0 °C. Then, the mixture was stirred at 60 °C for 2 h before addition of 2-(bromomethyl)pyridine hydrobromide (179 mg, 0.708 mmol). The reaction mixture was stirred at 60 °C for 3 h and extracted with ethyl acetate. The organic layer was dried over anhydrous Na_2SO_4, filtered and evaporated. The resulting crude residue was purified by column chromatography (ethyl acetate/Hex, 5%) on silica gel to give the thiophene **8ah** (139 mg, 57% yield) a yellow liquid.

^{1}H NMR (300 MHz, $CDCl_3$) δ 8.55 (d, *J* = 4.7 Hz, 1H), 8.12 (d, *J* = 8.2 Hz, 1H), 7.64 (td, *J* = 1.7 Hz, *J* = 7.9 z, 1H), 7.47 (d, *J* = 8.1 Hz, 1H), 7.05 (dd, *J* = 5.1 Hz, *J* = 7.2 Hz, 1H), 4.30 (q, *J* = 7.1 Hz, 2H), 3.26-3.39 (m, 1H), 2.56 (s, 3H), 2.11 (s, 2H), 1.75 (s, 2H), 1.19-1.45 (m, 9H); ^{13} C NMR (125 MHz, $CDCl_3$) δ 166.9, 166.0, 153.5, 149.3, 136.0, 135.1, 121.6, 120.1, 119.5, 105.2, 59.5, 56.1, 33.2, 32.7, 25.5, 25.1, 24.7, 16.8, 14.5; HRMS (EI) calcd for $C_{19}H_{24}N_2O_2S$ 344.1558, found 344.1561.

Ethyl 2-(benzylamino)-4-methyl-5-(pyridin-2-yl)thiophene-3-carboxylate (8ai)

To a stirred solution of potassium carbonate (102 mg, 0.670 mmol) in DMF (0.5 mL) was added ethyl acetoacetate (0.08 mL, 0.670 mmol). After stirring at rt for 2 h, benzyl isothiocyanate (0.08 mL, 0.670 mmol) was added dropwise at 0 °C. Then, the mixture was stirred at 60 °C for 2 h before addition 2-(bromomethyl)pyridine hydrobromide (169 mg, 0.670 mmol). The reaction mixture was stirred at 60 °C for 3 h and extracted with ethyl acetate. The organic layer was dried over anhydrous Na_2SO_4, filtered and evaporated. The resulting crude residue was purified by column chromatography (ethyl acetate/Hex, 5%) on silica gel to give the thiophene **8ai** (145 mg, 61% yield) a yellow solid.

mp. 103 - 104 °C; ^{1}H NMR (300 MHz, $CDCl_3$) δ 8.53 (d, *J* = 4.5 Hz, 1H), 8.40 (s, 1H), 7.58-7.67 (m, 1H), 7.45 (d, *J* = 8.0. Hz, 1H), 7.36 (s, 2H), 7.33 (s, 1H), 7.25-7.32 (m, 1H), 7.04 (dd, *J* = 5.4 Hz, *J* = 6.8 Hz, 1H), 4.50 (d, *J* = 5.7 Hz, 2H), 4.30 (q, *J* = 7.1 Hz, 2H), 2.57 (s, 3H), 1.36 (t, *J* = 7.1, 3H); ^{13} C NMR (125 MHz, $CDCl_3$) δ 166.8, 166.7, 153.4, 149.3, 137.2, 136.1, 135.1, 129.0, 128.8, 128.6, 127.7, 127.5, 121.7, 120.3, 59.6, 50.9, 16.7, 14.5; HRMS (EI) calcd for $C_{20}H_{20}N_2O_2S$ 352.1245, found 352.1241.

Ethyl 4-isopropyl-2-((3-methoxyphenyl)amino)-5-(pyridin-2-yl)thiophene-3-carboxylate (8aj)

To a stirred solution of potassium carbonate (84 mg, 0.605 mmol) in DMF (0.5 mL) was added ethyl 4-methyl-3-oxopentanoate (0.10 mL, 0.605 mmol). After stirring at rt for 2 h, 3-methoxyophenyl isothiocyanate (0.08 mL, 0.605 mmol) was added dropwise at 0 °C. Then, the mixture was stirred at 60 °C for 2 h before addition of 2-(bromomethyl)pyridine hydrobromide (153 mg, 0.6053 mmol). The reaction mixture

was stirred at 60 °C for 3 h and extracted with ethyl acetate. The organic layer was dried over anhydrous Na_2SO_4, filtered and evaporated. The resulting crude residue was purified by column chromatography (ethyl acetate/Hex 10%) to give the thiophene **8aj** (164 mg, 68% yield) a yellow liquid.

^{1}H NMR (300 MHz, $CDCl_3$) δ 10.37 (s, 1H), 8.60 (d, *J* = 5.1 Hz, 1H), 7.69 (td, *J* = 1.6 Hz, *J* = 7.7 Hz, 1H), 7.45 (d, *J* = 7.9 Hz, 1H), 7.23 (d, *J* = 8.2 Hz, 1H), 7.16 (dd, *J* = 5.0 Hz, *J* = 7.4 Hz, 1H), 6.96 (dd, *J* = 1.2 Hz, *J* = 7.8 z, 1H), 6.89 (t, *J* = 2.0 Hz, 1H), 6.62 (dd, *J* = 2.0 Hz, *J* = 8.3 Hz, 1H), 4.42 (q, *J* = 7.1 Hz, 2H), 3.80 (s, 3H), 3.58-3.70 (m, 1H), 1.46 (t, *J* = 7.1, 3H), 1.37 (s, 3H), 1.37 (d, *J* = 7.1 Hz, 6H); ^{13} C NMR (125 MHz, $CDCl_3$) δ 166.9, 160.8, 160.6, 153.7, 149.5, 143.2, 142.0, 136.1, 130.2, 123.9, 121.4, 121.3, 112.0, 109.1, 108.1, 105.4, 60.3, 55.4, 29.1, 21.7, 14.4; HRMS (EI) calcd for $C_{22}H_{24}N_2O_3S$ 396.1508, found 396.1507.

Methyl 4-cyclopropyl-2-((3-methoxyphenyl)amino)-5-(pyridin-2-yl)thiophene-3-carboxylate (8ak)

To a stirred solution of potassium carbonate (84 mg, 0.605 mmol) in DMF (0.5 mL) was added methyl-3-cycloproyl-3-oxopropionate (0.07 mL, 0.6053 mmol). After stirring at rt for 2 h, 3-methoxyophenyl isothiocyanate (0.08 mL, 0.6053 mmol) was added at 0 °C. Then, the mixture was stirred for 60 °C for 2 h before addition of 2-(bromomethyl)pyridine hydrobromide (153 mg, 0.6053 mmol). The reaction mixture was stirred at 60 °C for 3 h and extracted with ethyl acetate. The organic layer was dried over anhydrous Na_2SO_4, filtered and evaporated. The resulting crude residue was purified by column chromatography (ethyl acetate/Hex 10%) to give the thiophene **8ak** (186 mg, 81% yield) a yellow liquid.

^{1}H NMR (300 MHz, $CDCl_3$) δ 10.24 (s, 1H), 8.52 (d, *J* = 4.6 Hz, 1H), 7.82 (d, *J* = 8.1 Hz, 1H), 7.63-7.69 (m, 1H), 7.25 (t, *J* = 8.1 Hz, 1H), 7.06-7.13 (m, 1H), 6.99 (dd, *J* = 1.5 Hz, *J* = 7.9 Hz, 1H), 6.88 (s, 1H), 6.62 (dd, *J* = 1.9 Hz, *J* = 8.2 Hz, 1H), 3.93 (s, 3H), 3.81 (s, 3H), 1.98-2.10 (m, 1H), 0.91-1.02 (m, 2H), 0.31-0.39 (m, 2H); ^{13}C NMR (125 MHz, $CDCl_3$) δ 167.4, 160.6, 159.8, 152.7, 148.9, 141.7, 138.8, 135.5, 130.3, 124.7, 123.3, 120.8, 111.7, 110.2, 109.1, 105.2, 55.4, 51.2, 12.0, 10.3; HRMS (EI) calcd for $C_{21}H_{20}N_2O_3S$ 380.1195, found 380.1193.

Ethyl 2-((3-methoxyphenyl)amino)-5-(pyridin-2-yl)-4-(trifluoromethyl)thiophene-3-carboxylate (8al)

To a stirred solution of potassium carbonate (84 mg, 0.605 mmol) in DMF (0.5 mL) was added ethyl 4,4,4-trifluoro-3-oxobutanoate (0.09 mL, 0.605 mmol). After stirring at rt for 2 h, 3-methoxyophenyl isothiocyanate (0.08 mL, 0.605 mmol) was adde dropwise at 0 °C. Then, the mixture was stirred at 60 °C for 2 h before addition of 2-(bromomethyl)pyridine hydrobromide (153 mg, 0.605 mmol). The reaction mixture was stirred at 60 °C for 3 h and extracted with ethyl acetate. The organic layer was dried over anhydrous Na_2SO_4, filtered and evaporated. The resulting crude residue was purified by column chromatography (ethyl acetate/Hex 5%) to give the thiophene **8al** (36 mg, 14% yield) a brown liquid.

^{1}H NMR (300 MHz, $CDCl_3$) δ 10.06 (s, 1H), 8.59 (d, *J* = 4.6 Hz, 1H), 7.71 (td, *J* = 1.4 Hz, *J* = 7.8 Hz, 1H), 7.54 (d, *J* = 7.9 Hz, 1H), 7.21-7.30 (m, 2H), 6.92 (d, *J* = 8.0 Hz, 1H), 6.84 (s, 1H), 6.67 (dd, *J* = 2.0 Hz, *J* = 8.3 Hz, 1H), 4.38 (q, *J* = 7.1 Hz, 2H), 3.80 (s, 3H), 1.40 (t, *J* = 7.1, 3H); ^{13}C NMR (125 MHz, $CDCl_3$) δ 165.4, 160.7, 159.9, 151.3, 149.1, 141.3, 136.2, 130.5, 129.7, 125.1, 122.9, 121.7 (q, *J* = 224.1 Hz, CF_3),

112.2, 109.9, 106.0, 105.8, 60.9, 55.4, 13.9; HRMS (EI) calcd for $C_{20}H_{17}F_3N_2O_3S$ 422.0912, found 422.0906.

2-((3-Methoxyphenyl)amino)-4-phenyl-5-(pyridin-2-yl)thiophene-3-carbonitrile (8am)

To a stirred solution of potassium carbonate (84 mg, 0.605 mmol) in DMF (0.5 mL) was added benzoylacetonitrile (88 mg, 0.605 mmol). After stirring at rt for 2 h, 3-methoxyophenyl isothiocyanate (0.08 mL, 0.605 mmol) was added dropwise at 0 °C. Then, the mixture was stirred at 60 °C for 2 h before addition of 2-(bromomethyl)pyridine hydrobromide (153 mg, 0.605 mmol). The reaction mixture was stirred at 60 °C for 3 h and extracted with ethyl acetate. The organic layer was dried over anhydrous Na_2SO_4, filtered and evaporated. The resulting crude residue was purified by column chromatography (ethyl acetate/Hex 20%) to give the thiophene **8am** (78 mg, 32% yield) a yellow solid.

mp. 217 - 218 °C; ^{1}H NMR (300 MHz, $CDCl_3$) δ 8.47 (d, *J* = 3.1 Hz, 1H), 7.46 (s, 5H), 7.24-7.33 (m, 2H), 7.20 (s, 1H), 6.94-7.05 (m, 2H), 6.87 (s, 1H), 6.80 (d, *J* = 8.0 Hz, 1H), 6.69 (d, *J* = 8.3 Hz, 1H), 3.81 (s, 3H); ^{13} C NMR (125 MHz, $CDCl_3$) δ 160.8, 159.7, 151.7, 149.2, 141.1, 137.3, 135.9, 134.0, 130.6, 129.2, 129.0, 128.9, 124.8, 121.4, 120.5, 115.4, 111.2, 109.8, 105.0, 93.7, 55.4; HRMS (EI) calcd for $C_{23}H_{17}N_3OS$ 383.1092, found 383.1093.

2-(((3-methoxyphenyl)amino)((pyridin-2-ylmethyl)thio)methylene)malononitrile (7an)

To a solution of potassium carbonate (84 mg, 0.6053 mmol) in DMF (0.5 mL) was added malononitrile (40 mg, 0.6053 mmol). After stirring at rt for 1 h, 3-

methoxyophenyl isothiocyanate (0.08 mL, 0.6053 mmol) was added dropwise at 0 °C. Then, the mixture was stirred at 60 °C for 2 h before addition of 2-(bromomethyl)pyridine hydrobromide (153 mg, 0.605 mmol). The reaction mixture was stirred at 60 °C for 3 h and extracted with ethyl acetate. The organic layer was dried over anhydrous Na_2SO_4, filtered and evaporated. The resulting crude residue was purified by column chromatography (ethyl acetate/Hex 20%) to give product *N,S*-acetal **7an** (169 mg, 86% Yield) as a yellow solid.

mp. 131 - 132 °C; ^{1}H NMR (500 MHz, $CDCl_3$) δ 12.95 (s, 1H), 8.66 (d, *J* = 4.3 Hz, 1H), 7.84 (td, *J* = 1.6 Hz, *J* = 7.7 Hz, 1H), 7.39 – 7.43 (m, 1H), 7.34 – 7.39 (m, 2H), 6.89 – 6.92 (m, 1H), 6.84 (dd, *J* = 2.3 Hz, *J* = 4.5, Hz, 2H), 4.30 (s, 2H), 3.87 (s, 3H); ^{13}C NMR (126 MHz, $CDCl_3$) δ 169.5, 160.5, 156.7, 148.9, 139.1, 139.0, 130.4, 123.9, 123.8, 116.4, 115.5, 113.6, 112.3, 109.4, 55.7, 53.1, 38.0; HRMS (EI) calcd for $C_{17}H_{14}N_4OS$ 322.0888, found 322.0887.

4-amino-2-((3-methoxyphenyl)amino)-5-(pyridin-2-yl)thiophene-3-carbonitrile (8an)

A solution of compound **7an** (50 mg, 0.1551 mmol) in DMF (0.3 mL) was stirred at 100 °C for 3 h and extracted with ethyl acetate. The organic layer was dried over anhydrous Na_2SO_4, filtered and evaporated. The desired thiophene **8an** was obtained in 50% yield (25 mg) as a yellow solid.

mp. 167 - 168 °C; ^{1}H NMR (300 MHz, CDCl3) δ 8.43 (d, *J* = 4.1 Hz, 1H), 7.53 (td, *J* = 1.8 Hz, *J* = 8.1 Hz, 1H), 7.30 (t, *J* = 8.1 Hz, 1H), 7.08 (s, 1H), 6.99 (d, *J* = 8.2 Hz, 1H), 6.82 – 6.94 (m, 3H), 6.70 (dd, *J* = 1.5 Hz, *J* = 8.3 Hz, 1H), 6.29 (br, 2H), 3.84 (s, 3H); ^{13}C NMR (126 MHz, $CDCl_3$) δ 160.7, 156.8, 154.6, 148.2, 144.7, 140.8, 136.4,

130.6, 117.9, 117.3, 114.4, 111.6, 109.8, 105.2, 95.4, 84.8, 55.5; HRMS (EI) calcd for $C_{17}H_{14}N_4OS$ 322.0888, found 322.0890.

3-((3-methoxyphenyl)amino)-6,6-dimethyl-1-(pyridin-2-yl)-6,7-dihydrobenzo[c]thiophen-4(5H)-one (8ao)

To a stirred solution of potassium carbonate (84 mg, 0.605 mmol) in DMF (0.5 mL) was added 5,5-dimethylcyclohexane-1,3-dione (85 mg, 0.605 mmol). After stirring at rt for 2 h, 3-methoxyphenyl isothiocyanate (0.08 mL, 0.605 mmol) was added dropwise at 0 °C. Then, the mixture was stirred at 60 ℃ for 2 h before addition of 2-(bromomethyl)pyridine hydrobromide (153 mg, 0.605 mmol). The reaction mixture was stirred at 60 °C for 3 h and extracted with ethyl acetate. The organic layer was dried over anhydrous Na_2SO_4, filtered, and evaporated. The resulting crude residue was purified by column chromatography (ethyl acetate/Hex, 5%) on silica gel to give the thiophene **8ao** (60 mg, 25% yield) as a yellow liquid.

^{1}H NMR (300 MHz, $CDCl_3$) δ 11.43 (s, 1H), 8.54 (d, *J* = 5.5 Hz, 1H), 7.65 (td, *J* = 1.8 Hz, *J* = 7.8 Hz, 1H), 7.43 (d, *J* = 8.1 Hz, 1H), 7.28 (t, *J* = 8.2 Hz, 1H), 6.98 – 7.10 (m, 2H), 6.89 (t, *J* = 2.2 Hz, 1H), 6.64 (dd, *J* = 2.0 Hz, *J* = 8.2 Hz, 1H), 3.82 (s, 3H), 2.85 (s, 2H), 2.41 (s, 2H), 1.09 (s, 6H); ^{13}C NMR (126 MHz, $CDCl_3$) δ 195.0, 160.6, 159.2, 152.7, 149.4, 141.0, 136.3, 135.7, 130.3, 120.64, 120.60, 120.5, 115.7, 111.0, 109.2, 104.6, 55.4, 51.5, 40.3, 33.9, 28.5; HRMS (EI) calcd for $C_{22}H_{23}N_2O_2S$ 378.1402, found 378.1403.

Ethyl 2-((3-methoxyphenyl)amino)-4-methyl-5-(pyridin-4-yl)thiophene-3-carboxylate (8c)

To a solution of potassium carbonate (84 mg, 0.605 mmol) in DMF (0.5 mL) was added ethyl acetoacetate (0.08 mL, 0.605 mmol). After stirring at rt for 2 h, 3-methoxyophenyl isothiocyanate (0.08 mL, 0.6053 mmol) was added at 0 °C. Then, the mixture was stirred at 60 °C for 2 h before addition of 4-(bromomethyl)pyridine hydrobromide (153 mg, 0.6053 mmol). The reaction mixture was stirred at 0 °C and extracted with ethyl acetate. The organic layer was dried over anhydrous Na_2SO_4, filtered and evaporated. The resulting crude residue was purified by column chromatography (ethyl acetate/Hex 20%) to give product (173 mg, 80% yield) as a yellow liquid.

mp. 127 - 128 °C; ^{1}H NMR (300 MHz, $CDCl_3$) δ 10.42 (s, 1H), 8.58 (d, *J* = 6.0 Hz, 2H), 7.23 – 7.30 (m, 3H), 6.85 – 6.93 (m, 2H), 6.64 (dd, *J* = 2.1 Hz, *J* = 8.2 Hz, 1H), 4.37 (q, *J* = 7.1 Hz, 2H), 3.82 (s, 3H), 2.46 (s, 3H), 1.41 (t, *J* = 7.1 Hz, 3H); ^{13}C NMR (126 MHz, $CDCl_3$) δ 166.8, 160.6, 159.6, 149.7, 142.2, 141.5, 134.2, 130.3, 123.6, 116.8, 111.5, 109.03, 190.00, 104.9, 60.3, 55.3, 16.3, 14.3; HRMS (EI) calcd for $C_{20}H_{20}N_2O_3S$ 368.1195, found 368.1195.

Ethyl 2-((3-methoxyphenyl)amino)-4-methyl-5-(6-methylpyridin-2-yl)thiophene-3-carboxylate (8d)

To a solution of potassium carbonate (84 mg, 0.6053 mmol) in DMF (0.5 mL) was added ethyl acetoacetate (0.08 mL, 0.6053 mmol). After stirring at rt for 2 h, 3-methoxyophenyl isothiocyanate (0.08 mL, 0.6053 mmol) was added dropwise at 0 °C. Then, the mixture was stirred at 60 °C for 2h before addition of 2-(bromomethyl)-6-methylpyridine (112 mg, 0.6053 mmol). The reaction mixture was stirred at 60 °C

for 3 h and extracted with ethyl acetate. The organic layer was dried over anhydrous Na_2SO_4, filtered and evaporated. The resulting crude residue was purified by column chromatography (ethyl acetate/Hex 20 %) to give product (79 mg, 34% Yield) as a brown solid.

mp. 93 - 94 °C; ^{1}H NMR (300 MHz, $CDCl_3$) δ 10.45 (s, 1H), 7.54 (t, *J* = 7.7 Hz, 1H), 7.22 – 7.30 (m, 2H), 6.90 – 7.00 (m, 3H), 6.59 – 6.65 (m, 1H), 4.37 (q, *J* = 7.1 Hz, 2H), 3.82 (s, 3H), 2.57 (s, 3H), 2.55 (s, 3H), 1.41 (t, *J* = 7.1 Hz, 3H); ^{13}C NMR (126 MHz, $CDCl_3$) δ 167.1, 160.6, 160.2, 158.3, 152.4, 141.8, 136.4, 133.5, 130.2, 121.5, 120.4, 119.6, 111.6, 109.1, 108.9, 105.0, 60.1, 55.4, 24.5, 16.6, 14.4; HRMS (EI) calcd for $C_{21}H_{22}N_2O_3S$ 382.1351, found 382.1352.

Ethyl 2-((3-methoxyphenyl)amino)-4-methyl-5-(5-nitrofuran-2-yl)thiophene-3-carboxylate (8f)

To a solution of potassium carbonate (84 mg, 0.6053 mmol) in DMF (0.5 mL) was added ethyl acetoacetate (0.08 mL, 0.6053 mmol). After stirring at rt for 2 h, 3-methoxyophenyl isothiocyanate (0.08 mL, 0.6053 mmol) was added dropwise at 0 °C. Then, the reaction mixture was stirred at 60 °C for 2 h before addition of 2-(bromomethyl)-5-nitrofuran (124 mg, 0.6053 mmol). The reaction mixture was stirred at 60 °C for 3 h and extracted with ethyl acetate. The organic layer was dried over anhydrous Na_2SO_4, filtered and evaporated. The resulting crude residue was purified by column chromatography (ethyl acetate/Hex 15%) to give product (82 mg, 33% yield) as a red liquid.

mp. 117 - 118 °C; ^{1}H NMR (300 MHz, $CDCl_3$) δ 10.53 (s, 1H), 7.24 – 7.44 (m, 2H), 6.91 (d, *J* = 6.8 Hz, 1H), 6.82 (s, 1H), 6.69 (d, *J* = 7.5 Hz, 1H), 6.45 (s, 1H), 4.36 (d, *J* = 5.0 Hz, 2H), 3.82 (s, 3H), 2.58 (s, 3H), 1.41 (s, 3H); ^{13}C NMR (126 MHz, $CDCl_3$) δ

166.5, 161.0, 160.7, 152.8, 150.5, 140.9, 139.2, 130.5, 114.8, 111.9, 109.9, 109.1, 108.4, 107.5, 105.7, 60.7, 55.4, 16.5, 14.3; HRMS (EI) calcd for $C_{19}H_{18}N_2O_6S$ 402.0886, found 402.0885.

Methyl 5-(4-(ethoxycarbonyl)-5-((3-methoxyphenyl)amino)-3-methylthiophen-2-yl)furan-2-carboxylate (8g)

To a solution of potassium carbonate (84 mg, 0.6053 mmol) in DMF (0.5 mL) was added ethyl acetoacetate (0.08 mL, 0.6053 mmol). After at rt for 2 h, 3-methoxyophenyl isothiocyanate (0.08 mL, 0.6053 mmol) was added dropwise at 0 °C. Then, the mixture was stirred at 60 °C for 2 h before addition of methyl 5-(chloromethyl)furan-2-carboxylate (105 mg, 0.6053 mmol). The reaction mixture was stirred at 60 °C for 3 h and extracted with ethyl acetate. The organic layer was dried over anhydrous Na_2SO_4, filtered and evaporated. The resulting crude residue was purified by column chromatography (ethyl acetate/Hex 7%) to give product (51 mg, 20% yield) as a brown solid.

mp. 111 - 112 °C; ^1H NMR (300 MHz, $CDCl_3$) δ 10.43 (s, 1H), 7.24 – 7.35 (m, 1H), 7.22 (d, *J* = 3.4 Hz, 1H), 6.93 (d, *J* = 7.9 Hz, 1H), 6.85 (s, 1H), 6.66 (d, *J* = 7.3 Hz, 1H), 6.41 (d, *J* = 3.4 Hz, 1H), 4.36 (q, *J* = 7.0 Hz, 2H), 3.89 (s, 3H), 3.82 (s, 3H), 2.54 (s, 3H), 1.41 (t, *J* = 7.1 Hz, 3H); ^{13}C NMR (126 MHz, $CDCl_3$) δ 166.8, 160.7, 159.9, 159.0, 152.8, 142.4, 141.4, 135.6, 130.4, 120.1, 111.7, 109.4, 109.1, 108.6, 108.2, 105.3, 60.4, 55.4, 51.8, 16.4, 14.4; HRMS (EI) calcd for $C_{21}H_{21}NO_6S$ 415.1090, found 415.1092.

Ethyl 2-((3-methoxyphenyl)amino)-4-methyl-5-(3-(trifluoromethyl)-1,2,4-oxadiazol-5-yl)thiophene-3-carboxylate (8i)

To a solution of potassium carbonate (42 mg, 0.3026 mmol) in DMF (0.5 mL) was added ethyl acetoacetate (0.04 mL, 0.3026 mmol). After stirring at rt for 2 h, 3-methoxyophenyl isothiocyanate (0.04 mL, 0.3026 mmol) was added dropwise at 0 °C. Then, the mixture was stirred at 60 °C for 2 h before addition of 5-(chloromethyl)-3-(trifluoromethyl)-1,2,4-oxadiazole (56 mg, 0.3026 mmol)stirring at 0 °C for 0.5 h and extracted with ethyl acetate. The organic layer was dried over anhydrous Na_2SO_4, filtered and evaporated. The resulting crude residue was purified by column chromatography (ethyl acetate/Hex 20%) to give product (63 mg, 47% yield) as a yellow solid.

mp. 119 - 120 °C; ^{1}H NMR (300 MHz, $CDCl_3$) δ 10.72 (s, 1H), 7.32 (t, *J* = 8.2 Hz, 1H), 6.95 (dd, *J* = 1.8 Hz, *J* = 7.9 Hz, 1H), 6.87 (t, *J* = 2.2 Hz, 1H), 6.74 (dd, *J* = 2.1 Hz, *J* = 8.3 Hz, 1H), 4.40 (q, *J* = 7.1 Hz, 2H), 3.84 (s, 3H), 2.83 (s, 3H), 1.44 (t, *J* = 7.1 Hz, 3H); ^{13}C NMR (126 MHz, $CDCl_3$) δ 174.0, 166.4, 163.8, 161.2 (q, J = 39.63 Hz, C), 160.7, 147.6, 140.3, 130.6, 118.1 (q, J = 272.79, CF_3), 112.4, 110.6, 109.5, 106.2, 101.1, 60.9, 55.4, 16.8, 14.2; HRMS (EI) calcd for $C_{18}H_{16}F_3N_3O_4S$ 427.0814, found 427.0813.

Ethyl 2-((3-methoxyphenyl)amino)-4-methyl-5-(1-methyl-1H-imidazol-2-yl)thiophene-3-carboxylate (8k)

To a stirred solution of potassium carbonate (84 mg, 0.605 mmol) in DMF (0.5 mL) was added ethyl acetoacetate (0.08 mL, 0.605 mmol). After stirring at rt for 2 h, 3-methoxyphenyl isothiocyanate (0.08 mL, 0.605 mmol) was added dropwise at 0 °C. Then, the mixture was stirred at 60 °C for 2 h before addition of 2-(chloromethyl)-1-

methyl-1H-imidazole (79 mg, 0.605 mmol). The reaction mixture was stirred at 60 °C for 3 h and extracted with ethyl acetate. The organic layer was dried over anhydrous Na_2SO_4, filtered, and evaporated. The resulting crude residue was purified by column chromatography (ethyl acetate/Hex, 5%) on silica gel to give the thiophene (18 mg, 8% yield) as a yellow liquid.

^{1}H NMR (300 MHz, $CDCl_3$) δ 10.40 (s, 1H), 7.22 (d, *J* = 8.1 Hz, 1H), 7.13 (d, *J* = 1.2 Hz, 1H), 6.99 (d, *J* = 1.2 Hz, 1H), 6.85 – 6.95 (m, 2H), 6.59 – 6.65 (m, 1H), 4.36 (q, *J* = 7.1 Hz, 2H), 3.80 (s, 3H), 3.58 (s, 3H), 2.25 (s, 3H), 1.40 (t, *J* = 7.1 Hz, 3H); ^{13}C NMR (126 MHz, $CDCl_3$) δ 166.8, 160.6, 160.2, 141.7, 141.1, 137.3, 130.3, 129.0, 121.6, 111.3, 109.2, 107.5, 107.4, 104.5, 60.2, 55.4, 33.6, 16.9, 14.4; HRMS (EI) calcd for $C_{19}H_{21}N_3O_3S$ 371.1304, found 371.1305.

Ethyl 2-((3-methoxyphenyl)amino)-4-methyl-5-(4-nitrophenyl)thiophene-3-carboxylate (8p)

To a solution of potassium carbonate (84 mg, 0.605 mmol) in DMF (0.5 mL) was added ethyl acetoacetate (0.08 mL, 0.605 mmol). After stirring at rt for 2 h, 3-methoxyophenyl isothiocyanate (0.08 mL, 0.605 mmol) was added dropwise at 0 °C. Then, the mixture was stirred at 60 °C for 2 h before addition of 4-nitrobenzyl bromide (131 mg, 0.605 mmol). The reaction mixture was stirred at 60 °C for 3 h and extracted with ethyl acetate. The organic layer was dried over anhydrous Na_2SO_4, filtered and evaporated. The resulting crude residue was purified by column chromatography (ethyl acetate/Hex 20%) to give product (104 mg, 42% yield) as an orange solid.

mp. 163 - 164 °C; ^{1}H NMR (300 MHz, $CDCl_3$) δ 10.42 (s, 1H), 8.24 (d, *J* = 8.7 Hz, 2H), 7.53 (d, *J* = 8.7 Hz, 2H), 7.30 (d, *J* = 8.1 Hz, 1H), 6.86 – 6.95 (m, 2H), 6.66 (dd,

J = 2.1 Hz, *J* = 8.3 Hz, 1H), 4.39 (q, *J* = 7.1 Hz, 2H), 3.83 (s, 3H), 2.45 (s, 3H), 1.42 (t, *J* = 7.1 Hz, 3H); ^{13}C NMR (126 MHz, $CDCl_3$) δ 166.8, 160.6, 159.8, 146.0, 141.5, 141.2, 134.2, 130.3, 129.7, 123.8, 117.4, 111.6, 109.08, 109.02, 105.1, 60.3, 55.3, 16.4, 14.3; HRMS (EI) calcd for $C_{21}H_{20}N_2O_5S$ 412.1093, found 412.1074.

5-((benzylthio)((3-methoxyphenyl)amino)methylene)-2,2-dimethyl-1,3-dioxane-4,6-dione (9a)

To a stirred solution of potassium carbonate (84 mg, 0.605 mmol) in DMF (0.5 mL) was added 2,2-dimethyl-1,3-dioxane-4,6-dione (87 mg, 0.605 mmol). After stirring at rt for 2 h, 3-methoxyphenyl isothiocyanate (0.08 mL, 0.605 mmol) was added dropwise at 0 °C. Then, the mixture was stirred at 60 °C for 2 h before addition of benzyl bromide (0.07 mL, 0.605 mmol). The reaction mixture was stirred at 60 °C for 3 h and extracted with ethyl acetate. The organic layer was dried over anhydrous Na_2SO_4, filtered, and evaporated. The resulting crude residue was purified by column chromatography (ethyl acetate/Hex, 5%) on silica gel to give **9a** (118 mg, 49% yield) as a yellow liquid.

^{1}H NMR (300 MHz, $CDCl_3$) δ 12.81 (s, 1H), 7.34 (t, *J* = 8.1 Hz, 1H), 7.25 (d, *J* = 1.7 Hz, 2H), 7.15 – 7.19 (m, 2H), 6.90 (dd, *J* = 2.0 Hz, J = 8.3 Hz, 2H), 6.84 (d, *J* = 2.0 Hz, 1H), 4.02 (s, 2H), 3.81 (s, 3H), 1.71 (s, 6H); ^{13}C NMR (126 MHz, $CDCl_3$) δ 175.40, 175.39, 160.5, 138.4, 134.9, 130.4, 129.3, 128.9, 128.2, 117.6, 114.1, 111.0, 103.3, 87.4, 55.7, 40.2, 26.6; HRMS (EI) calcd for $C_{21}H_{21}NO_5S$ 399.1140, found 399.1157.

5-(((3-methoxyphenyl)amino)((pyridin-2-ylmethyl)thio)methylene)-2,2-dimethyl-1,3-dioxane-4,6-dione (9b)

To a stirred solution of potassium carbonate (84 mg, 0.605 mmol) in DMF (0.5 mL) was added 2,2-dimethyl-1,3-dioxane-4,6-dione (87 mg, 0.605 mmol). After stirring at rt for 2 h, 3-methoxyphenyl isothiocyanate (0.08 mL, 0.605 mmol) was added dropwise at 0 °C. Then, the mixture was stirred at 60 °C for 2 h before addition of 2-(bromomethyl)pyridine hydrobromide (153 mg, 0.605 mmol). The reaction mixture was stirred at 60 °C for 3 h and extracted with ethyl acetate. The organic layer was dried over anhydrous Na_2SO_4, filtered, and evaporated. The resulting crude residue was purified by column chromatography (ethyl acetate/Hex, 5%) on silica gel to give **9b** (108 mg, 44% yield) as a yellow liquid.

^{1}H NMR (300 MHz, $CDCl_3$) δ 12.85 (s, 1H), 8.50 (d, *J* = 4.7 Hz, 1H), 7.62 (td, *J* = 1.4 Hz, J = 7.7 Hz, 1H), 7.25 – 7.34 (m, 2H), 7.15 – 7.23 (m, 3H), 6.81 – 6.90 (m, 4H), 4.15 (s, 2H), 3.80 (s, 3H), 1.73 (s, 6H); ^{13}C NMR (126 MHz, $CDCl_3$) δ 175.5, 164.1, 160.5, 155.4, 149.7, 138.8, 137.2, 130.4, 123.6, 122.9, 117.3, 113.8, 110.7, 103.3, 87.4, 55.6, 41.5, 26.6; HRMS (EI) calcd for $C_{20}H_{20}N_2O_5S$ 400.1093, found 400.1084.

5-(((3-methoxyphenyl)amino)((pyridin-3-ylmethyl)thio)methylene)-2,2-dimethyl-1,3-dioxane-4,6-dione (9c)

To a stirred solution of potassium carbonate (84 mg, 0.605 mmol) in DMF (0.5 mL) was added 2,2-dimethyl-1,3-dioxane-4,6-dione (87 mg, 0.605 mmol). After stirring at rt for 2 h, 3-methoxyphenyl isothiocyanate (0.08 mL, 0.605 mmol) was added dropwise at 0 °C. Then, the mixture was stirred at 60 °C for 2 h before addition of 3-(bromomethyl)pyridine hydrobromide (153 mg, 0.605 mmol). The reaction mixture was stirred at 60 °C for 3 h and extracted with ethyl acetate. The organic layer was

dried over anhydrous Na_2SO_4, filtered, and evaporated. The resulting crude residue was purified by column chromatography (ethyl acetate/Hex, 5%) on silica gel to give **9c** (71 mg, 29% yield) as a yellow liquid.

^{1}H NMR (300 MHz, $CDCl_3$) δ 12.84 (s, 1H), 8.49 (d, *J* = 3.8 Hz, 1H), 8.39 (s, 1H), 7.55 (d, *J* = 7.8 Hz, 1H), 7.36 (t, *J* = 8.1 Hz, 1H), 7.22 (dd, *J* = 4.8 Hz, *J* = 7.8 Hz, 1H), 6.88 – 6.94 (m, 2H), 6.84 (s, 1H), 4.01 (s, 2H), 3.82 (s, 3H), 1.72 (s, 6H); ^{13}C NMR (126 MHz, $CDCl_3$) δ 174.4, 164.1, 160.6, 150.3, 149.5, 138.3, 136.7, 131.0, 130.5, 123.7, 117.5, 114.2, 111.0, 103.5, 87.7, 55.7, 37.1, 26.6; HRMS (EI) calcd for $C_{20}H_{20}N_2O_5S$ 400.1093, found 400.1095.

5-(Methoxy((3-methoxyphenyl)amino)methylene)-2,2-dimethyl-1,3-dioxane-4,6-dione (9ba)

A solution of 5-((benzylthio)((3-methoxyphenyl)amino)methylene)-2,2-dimethyl-1,3-dioxane-4,6-dione **9b** (20 mg, 0.050 mmol) in MeOH (1 mL) was stirred at 60 °C for 3 h. Then, the mixture was extracted with ethyl acetate. The organic layer was dried over anhydrous Na_2SO_4, filtered, and evaporated. The resulting crude residue was purified by column chromatography (ethyl acetate/Hex, 20%) on silica gel to give the desired *N,O*-acetal **9ba** (5 mg, 33% yield) as a yellow liquid. 30% (6 mg, 0.015 mmol) of the starting material was recovered.

^{1}H NMR (300 MHz, $CDCl_3$) δ 11.91 (s, 1H), 7.30 (t, *J* = 8.1 Hz, 1H), 6.92 (d, *J* = 7.4 Hz, 2H), 6.78 – 6.87 (m, 1H), 4.16 (s, 3H), 3.82 (s, 3H), 1.77 (s, 6H); ^{13}C NMR (126 MHz, $CDCl_3$) δ 171.5, 164.2, 160.2, 136.0, 130.1, 115.5, 112.7, 109.2, 103.2, 76.0, 62.9, 55.5, 29.7, 26.3; HRMS (EI) calcd for $C_{15}H_{17}NO_6$ 307.1056, found 307.1055.

^{1}H and ^{13}C NMR of compound **8aa**

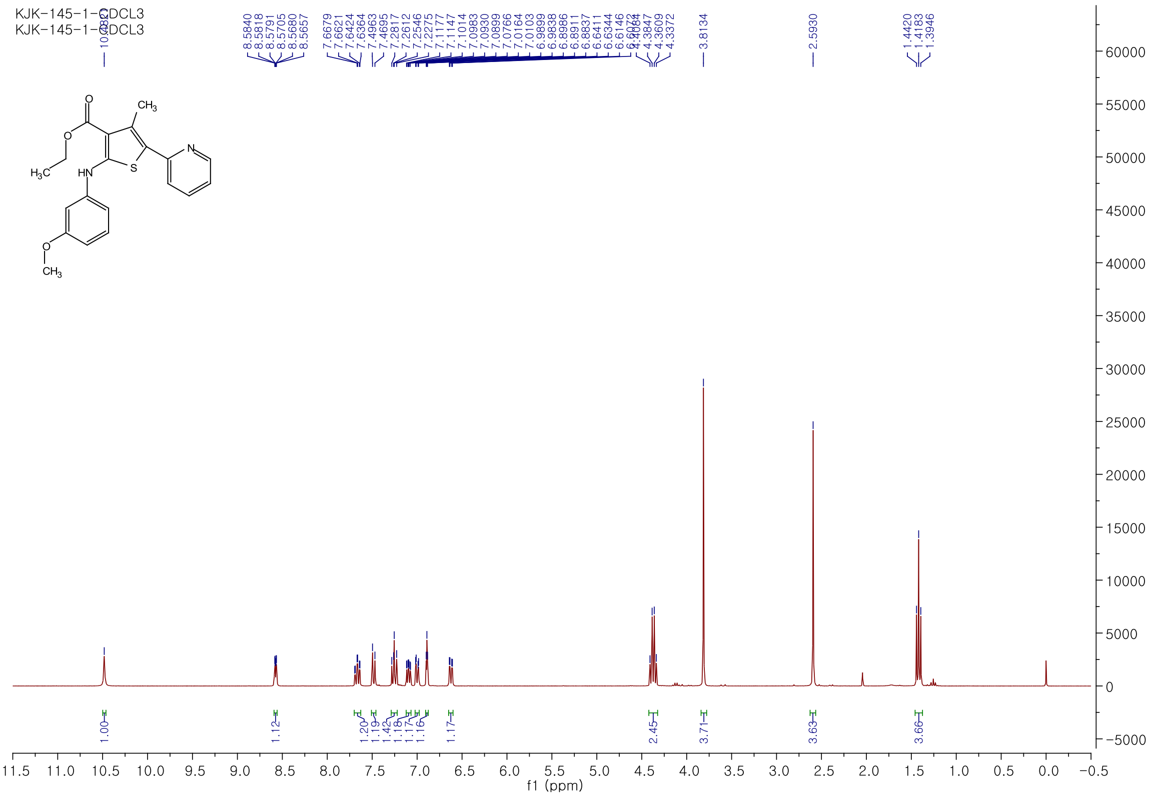

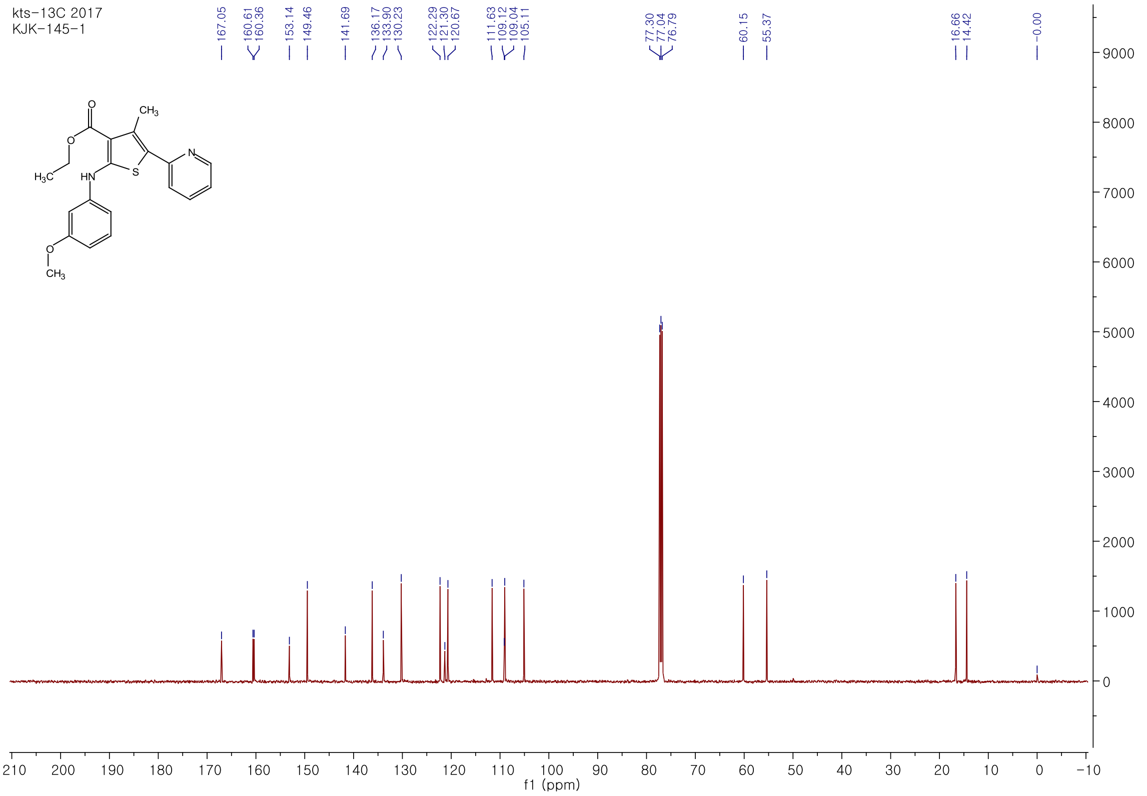

^{1}H and ^{13}C NMR of compound **8ab**

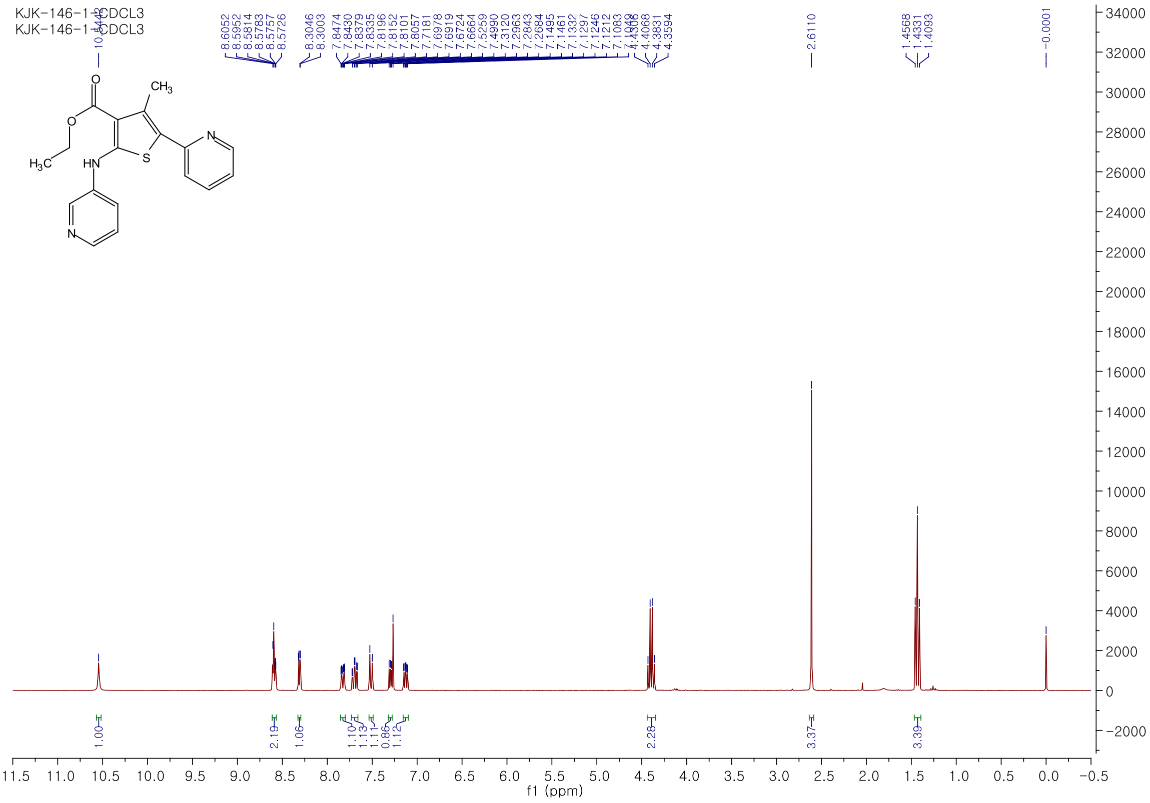

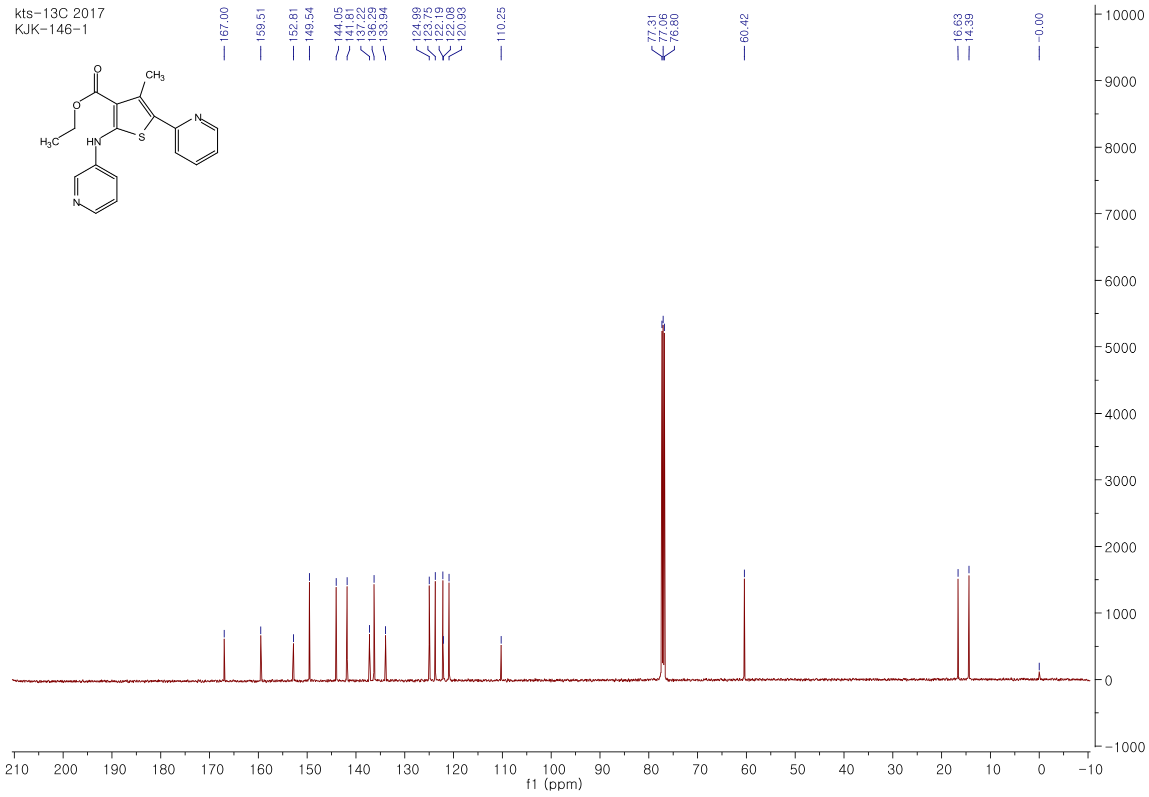

^{1}H and ^{13}C NMR of compound **8ac**

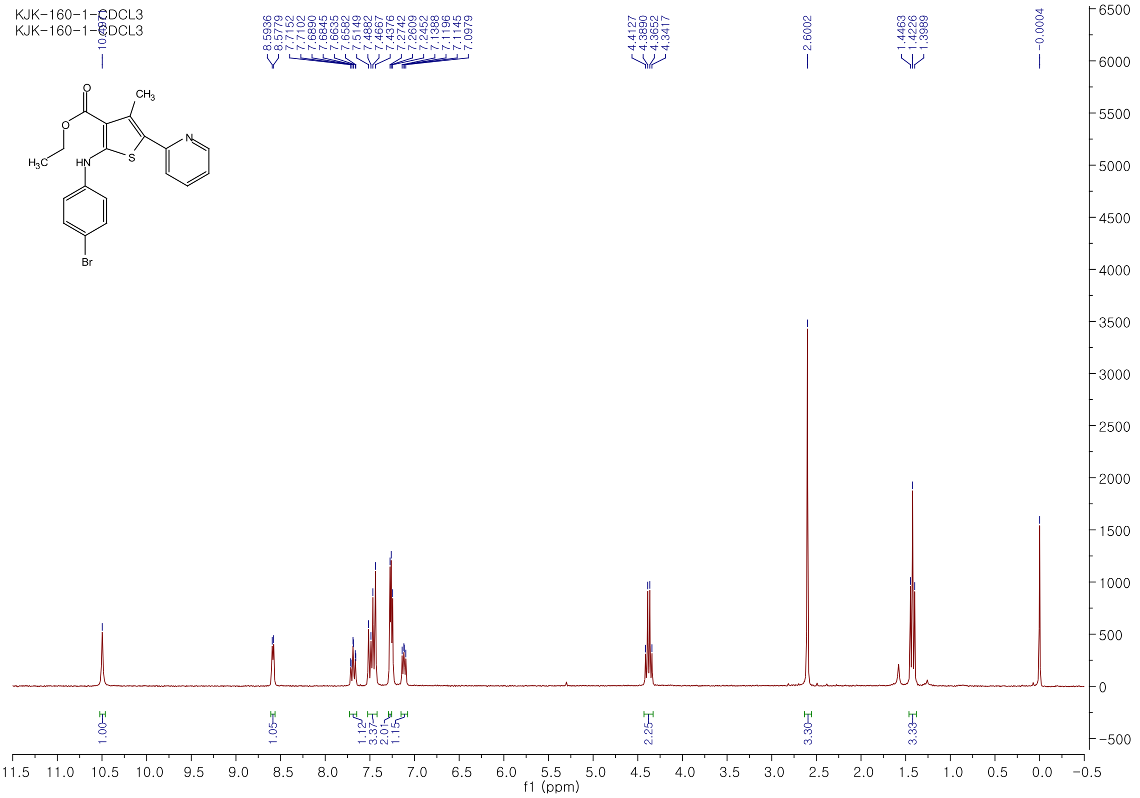

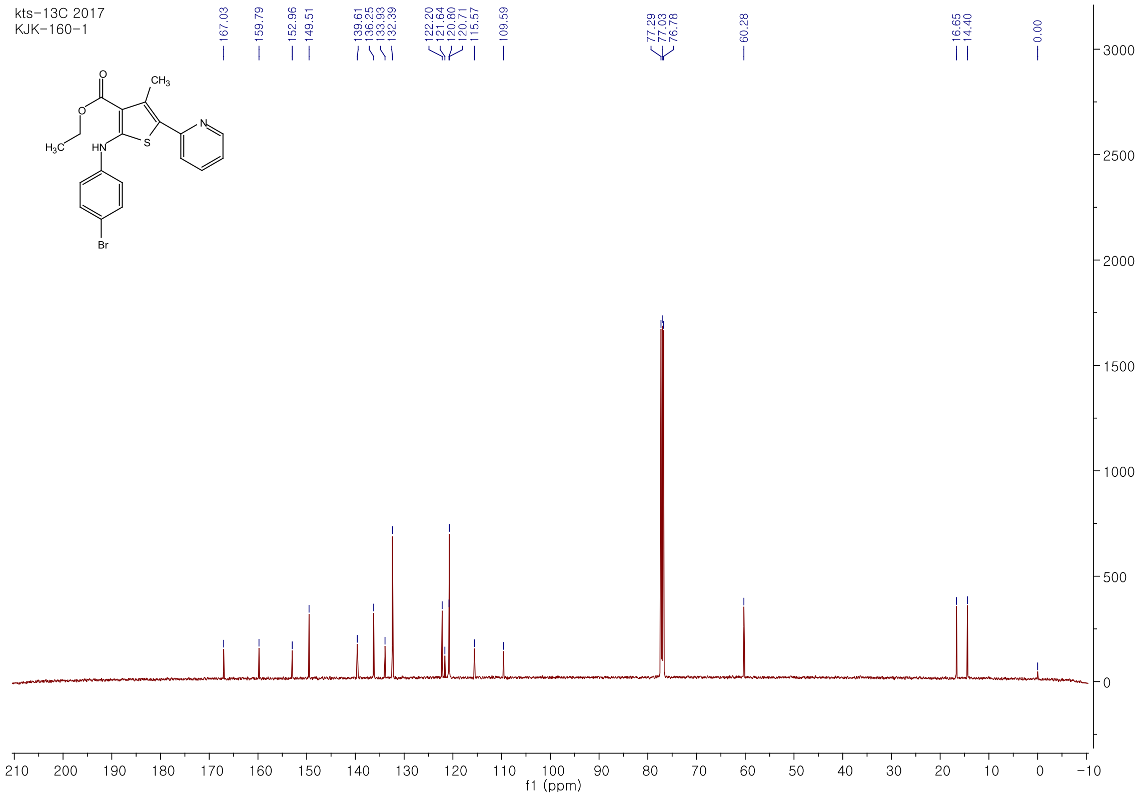

^{1}H and ^{13}C NMR of compound **8ad**

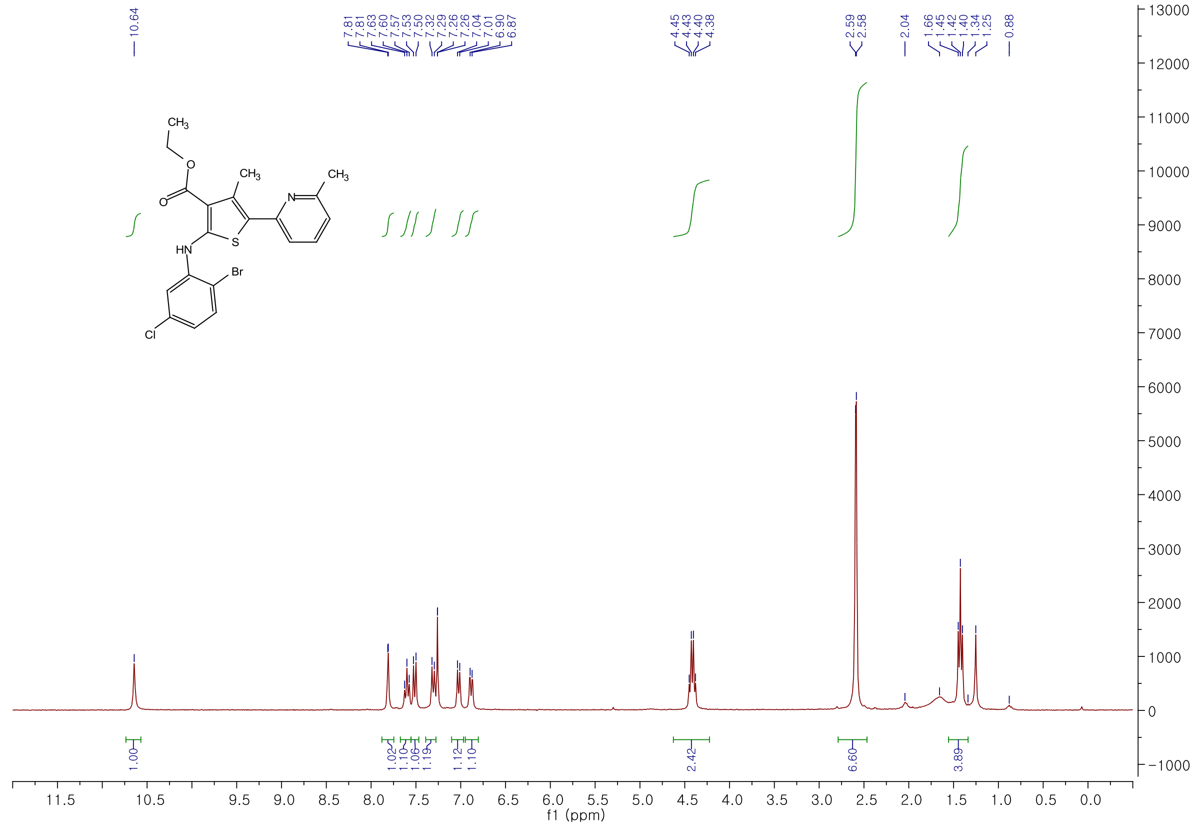

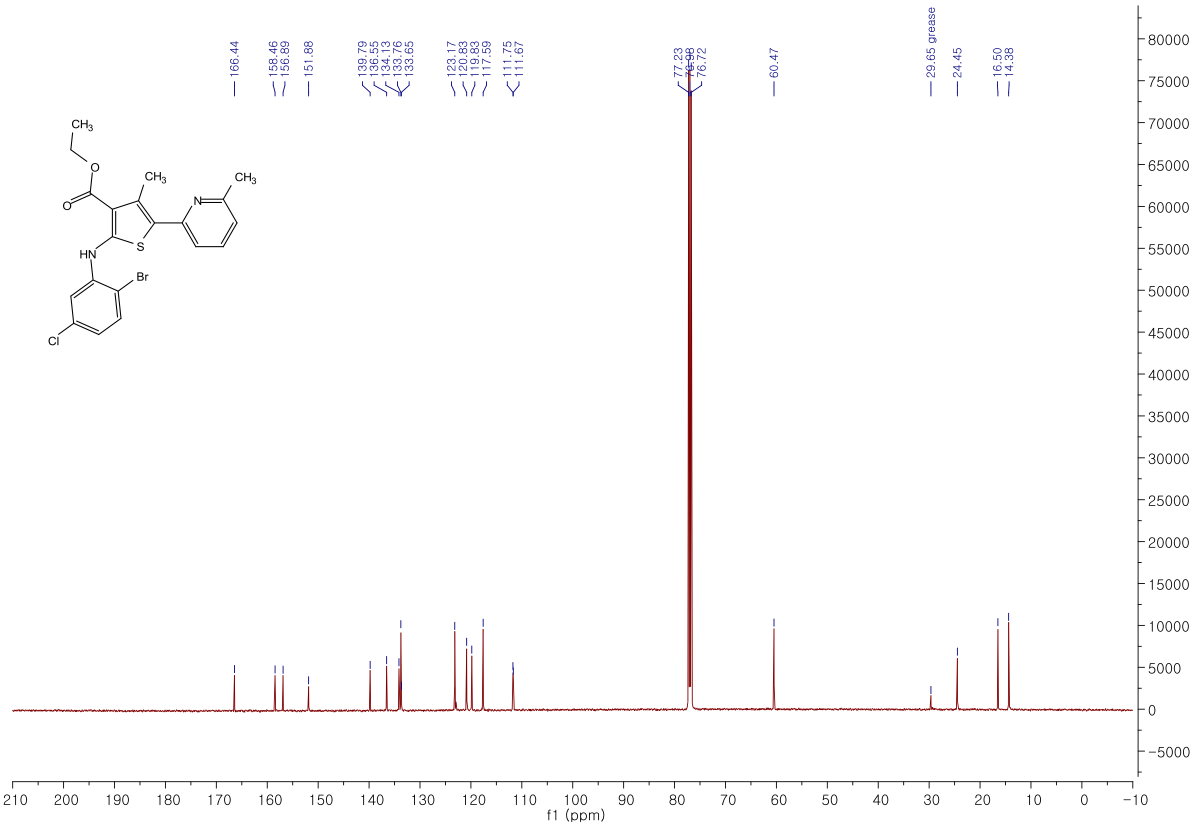

^{1}H and ^{13}C NMR of compound **8ae**

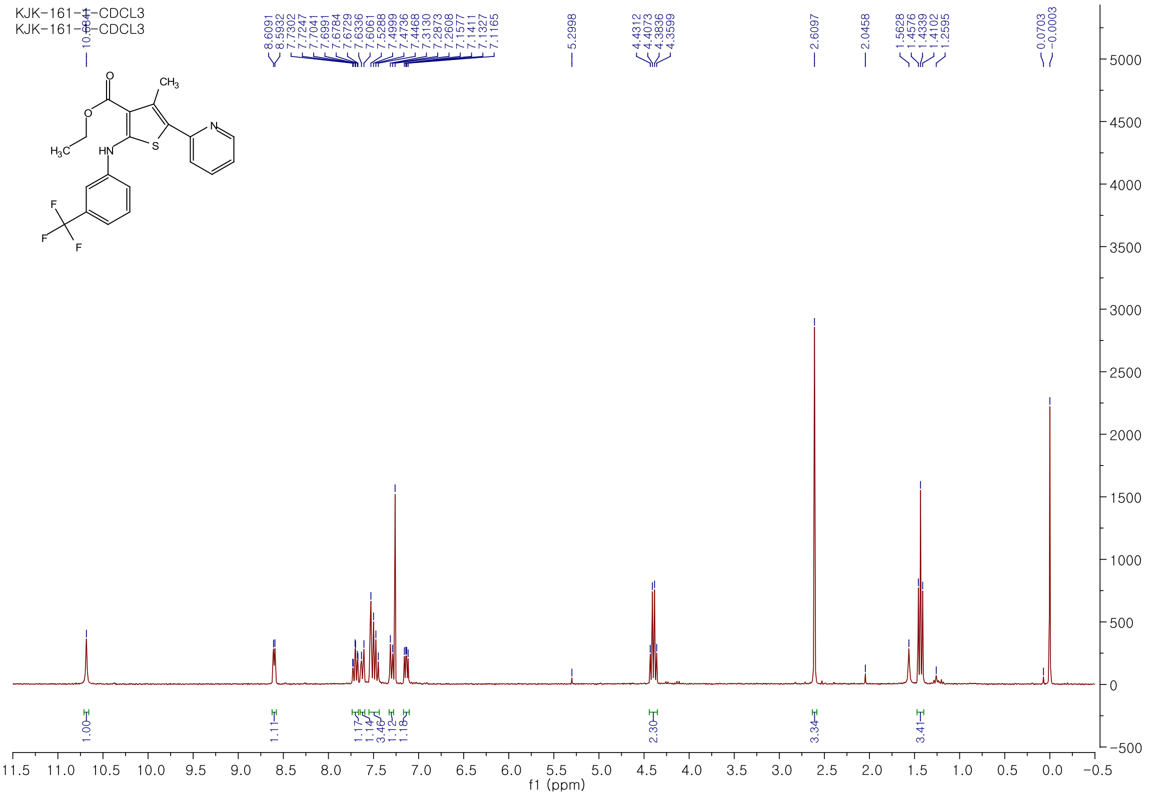

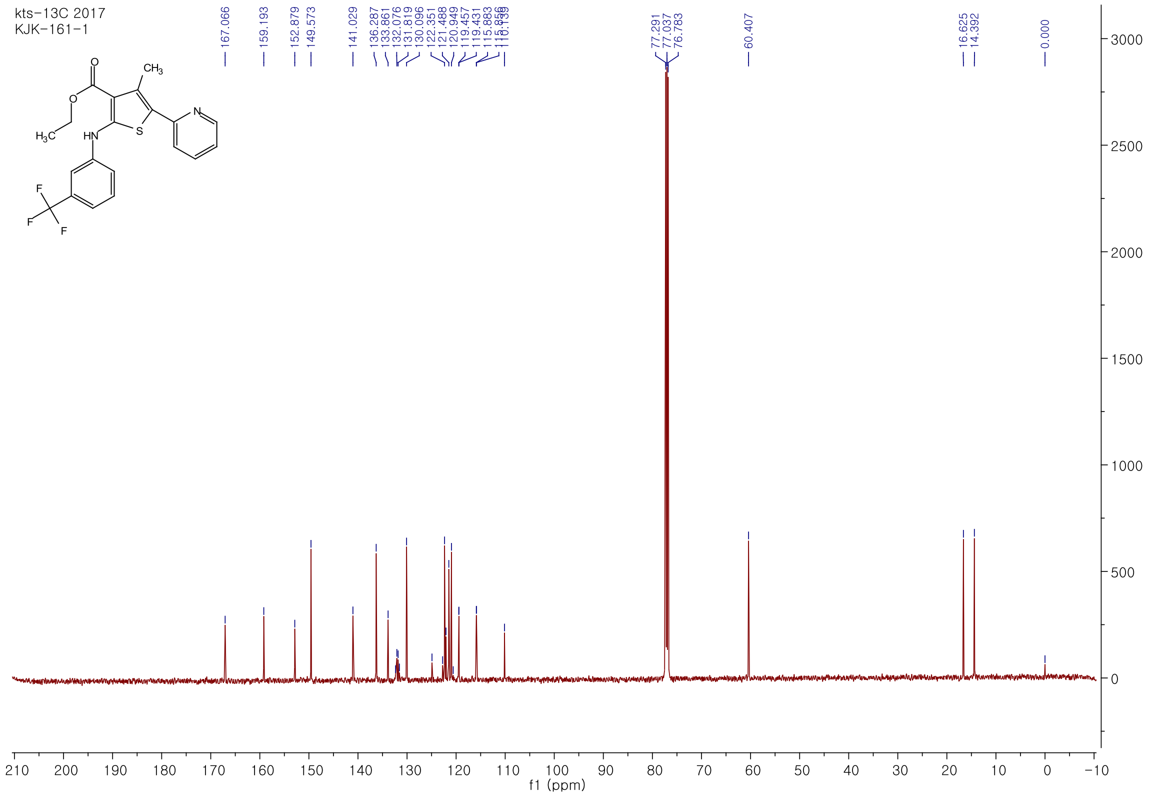

^{1}H and ^{13}C NMR of compound **8af**

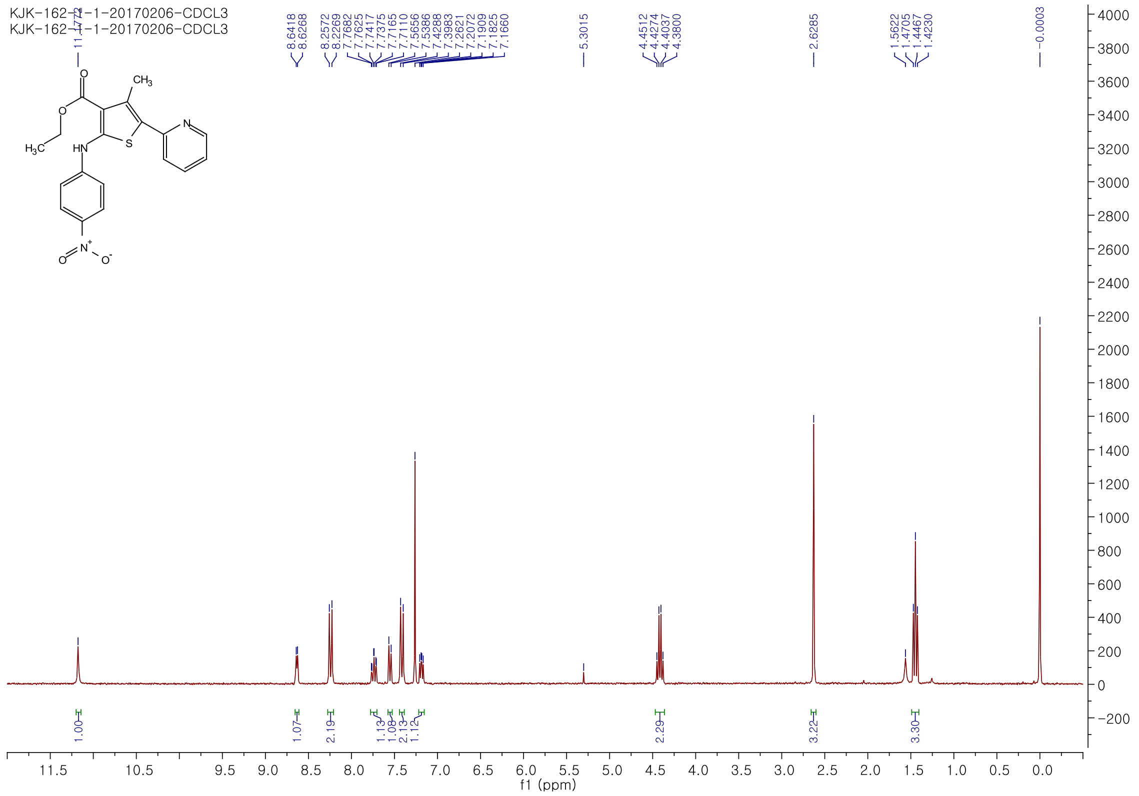

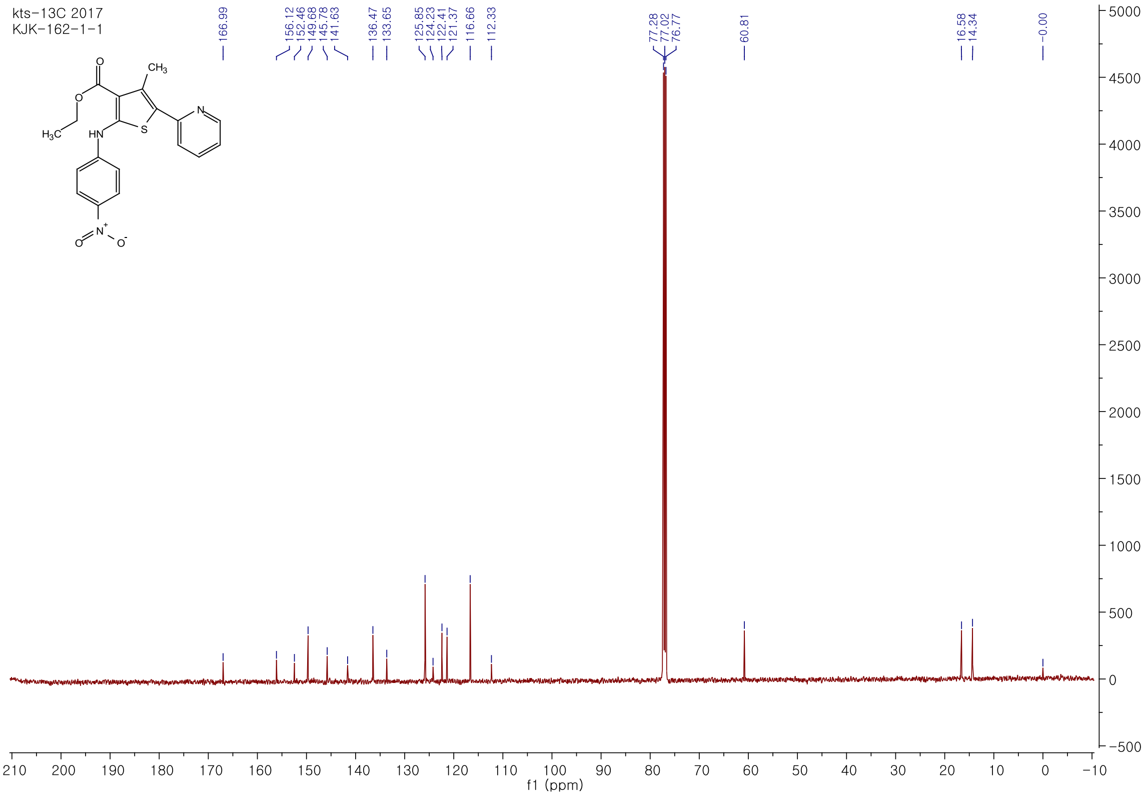

^{1}H and ^{13}C NMR of compound **8ag**

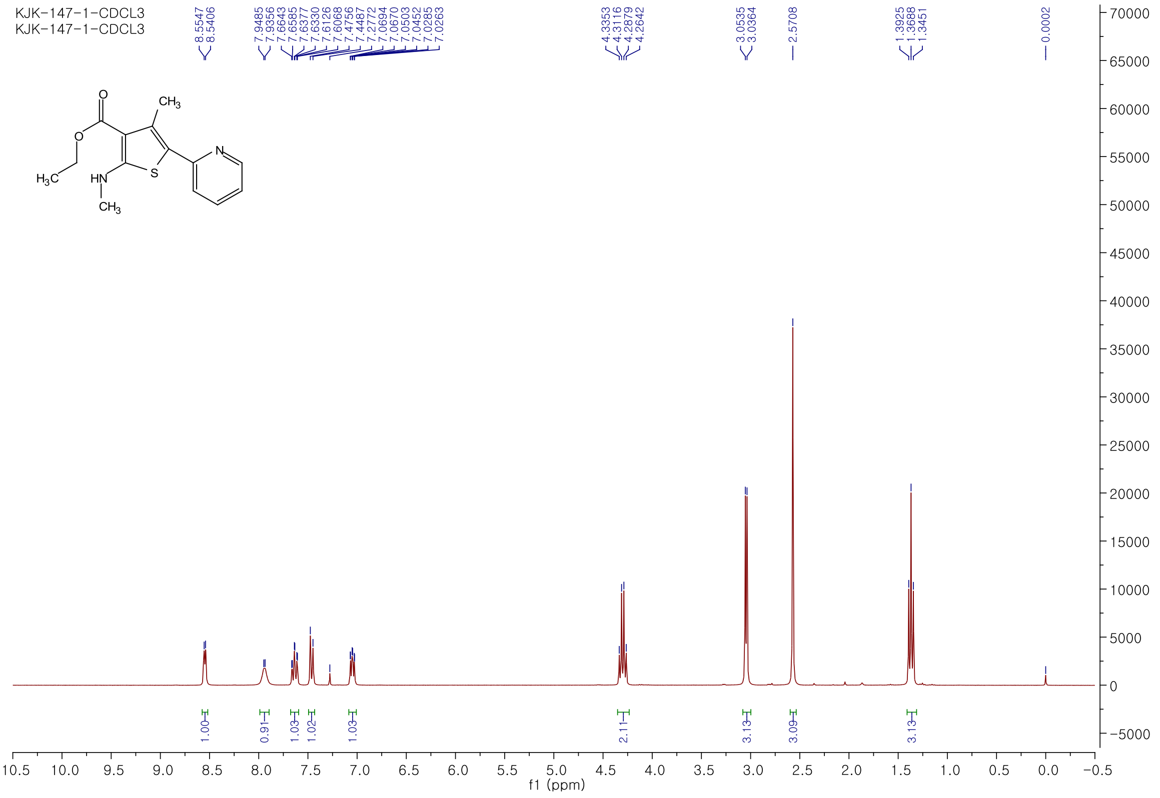

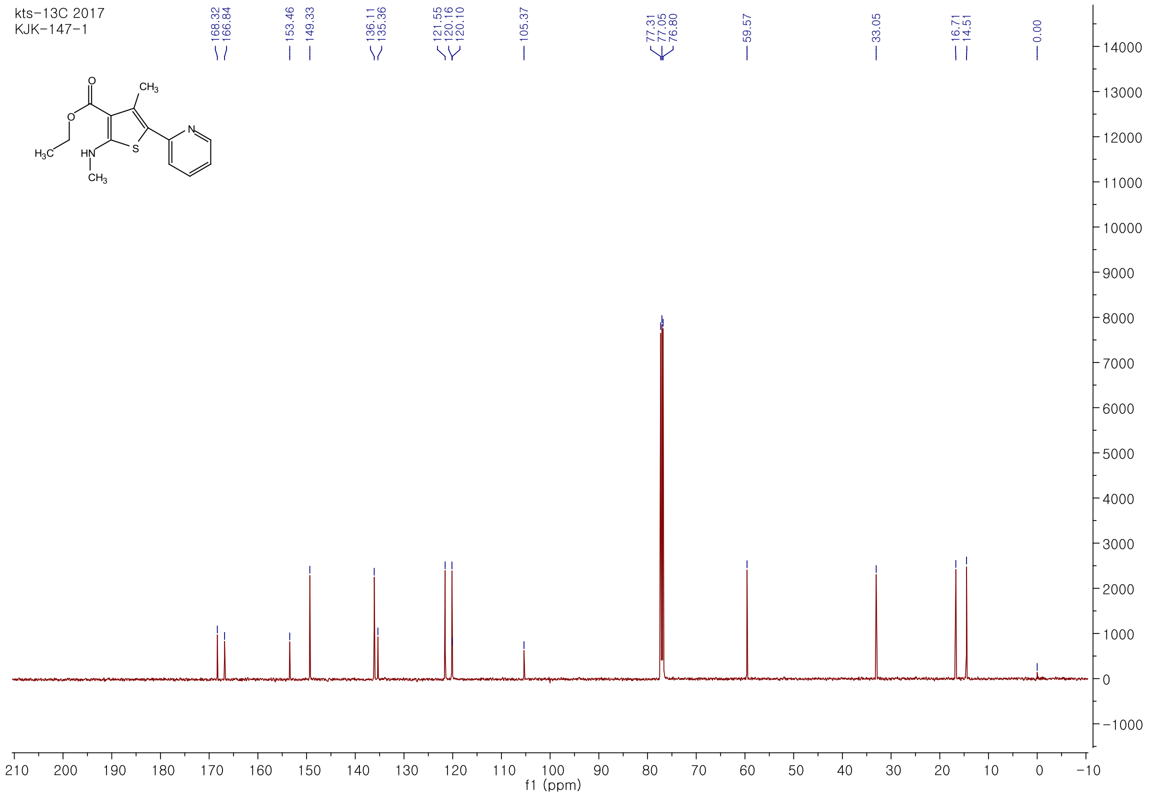

^1H and ^{13}C NMR of compound **8ah**

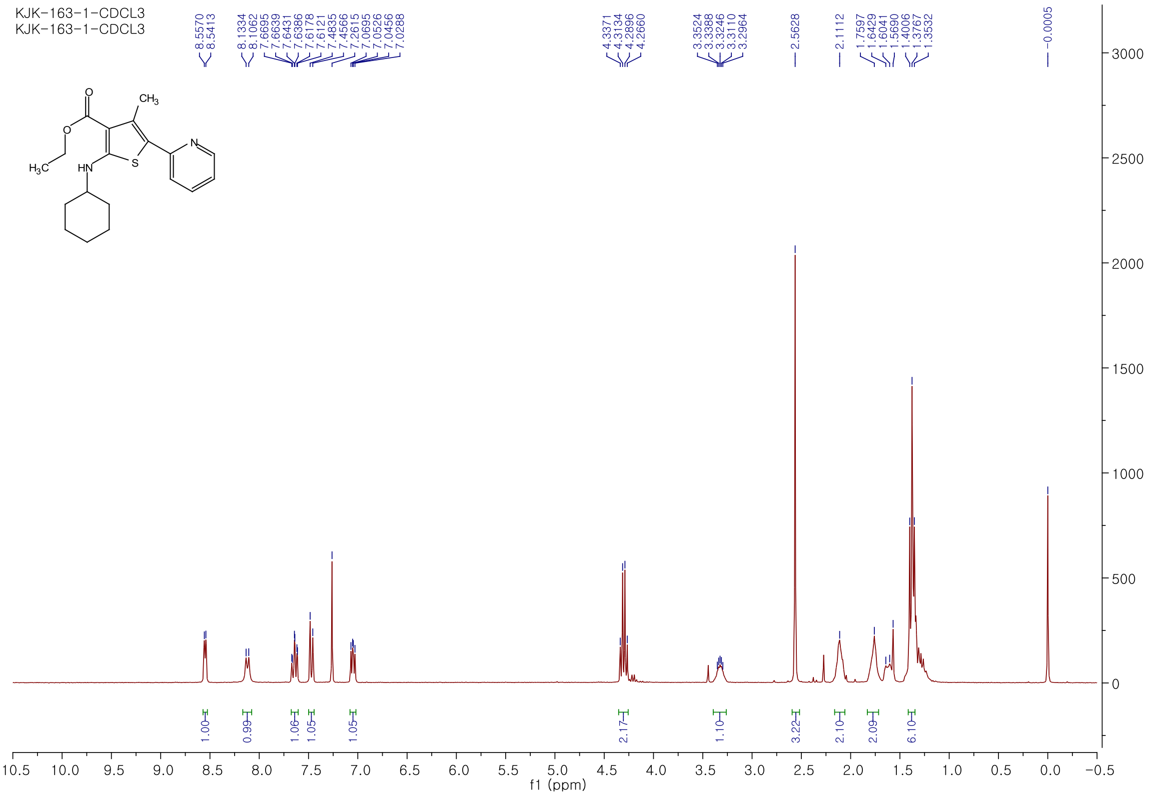

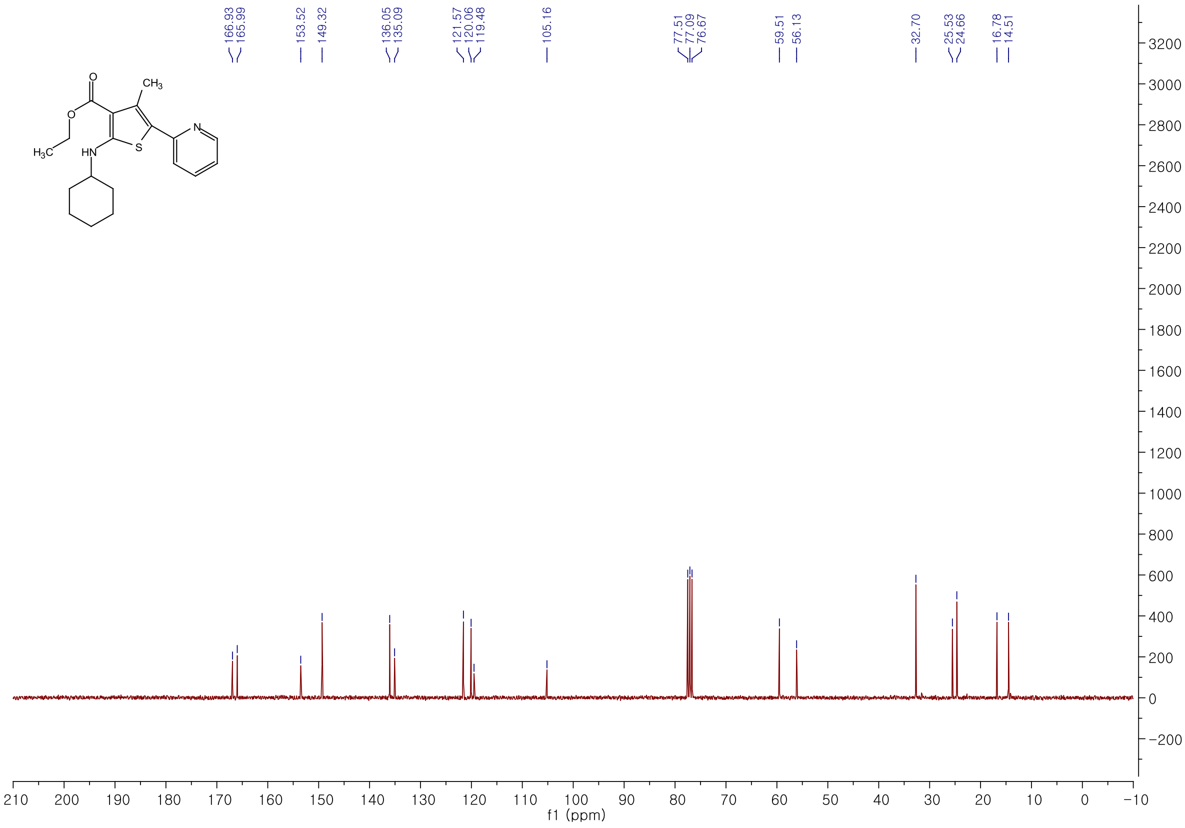

^{1}H and ^{13}C NMR of compound **8ai**

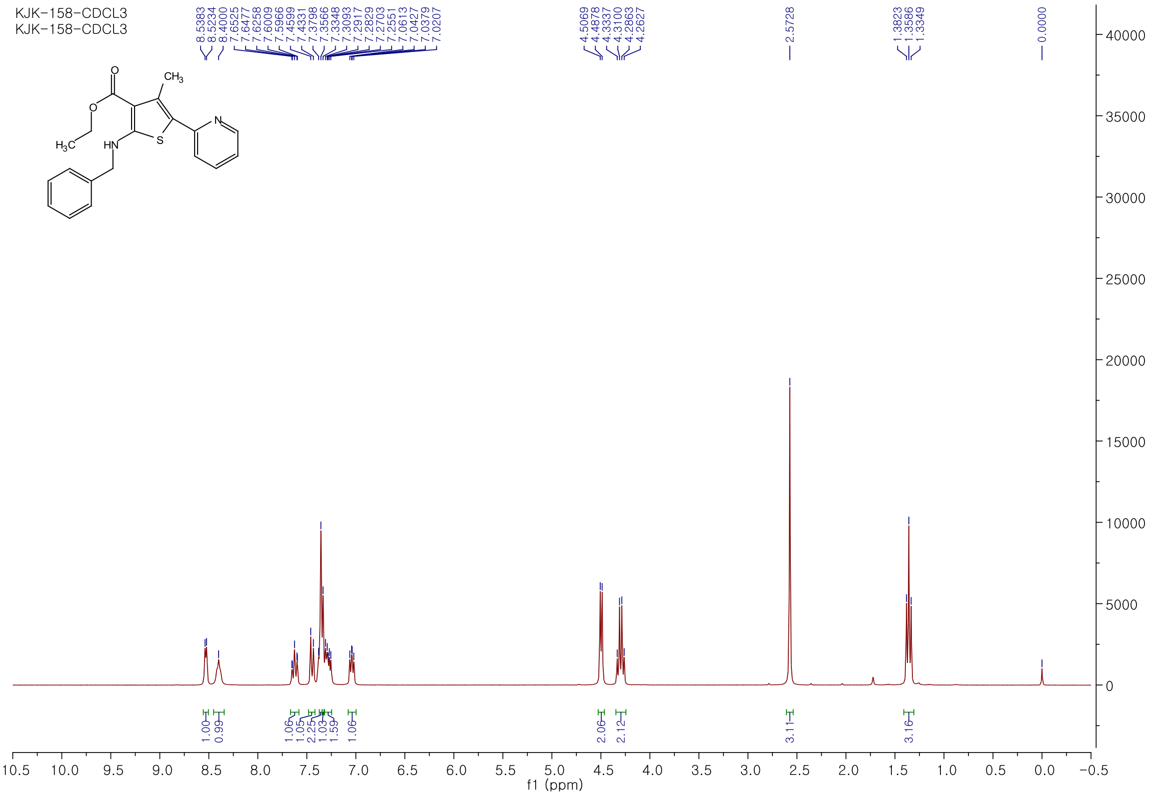

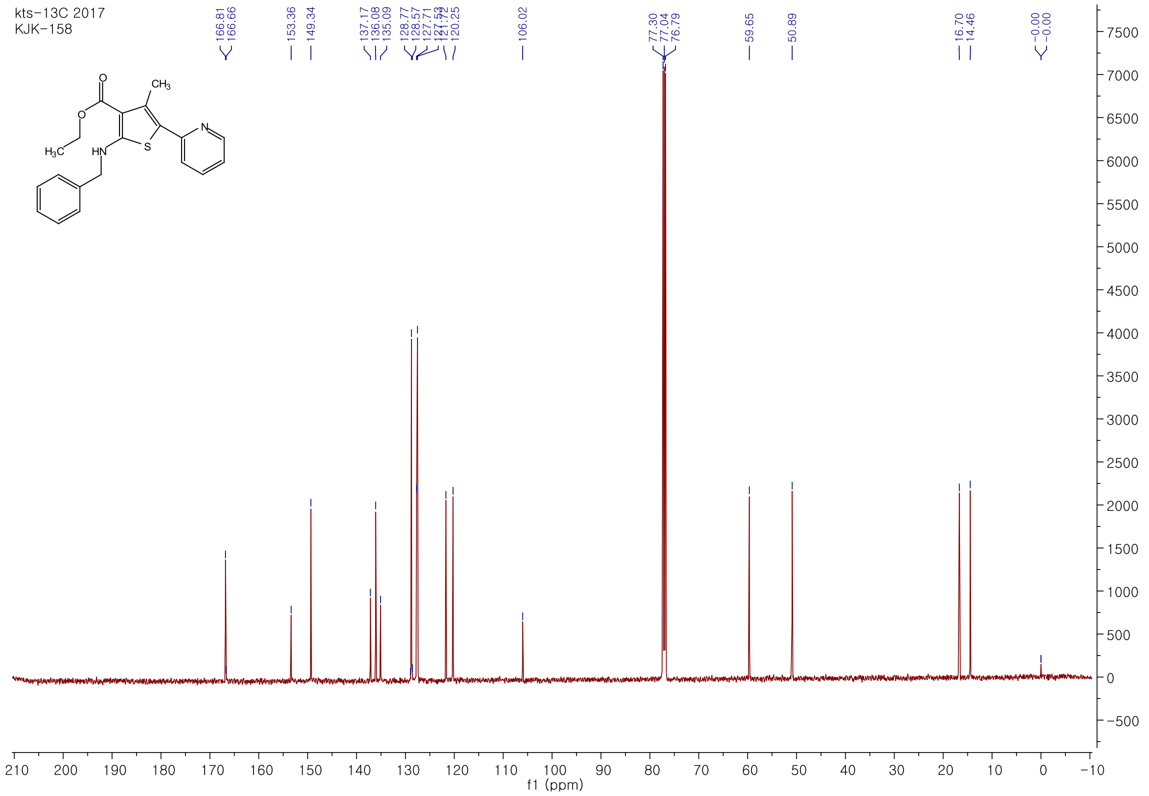

^{1}H and ^{13}C NMR of compound **8aj**

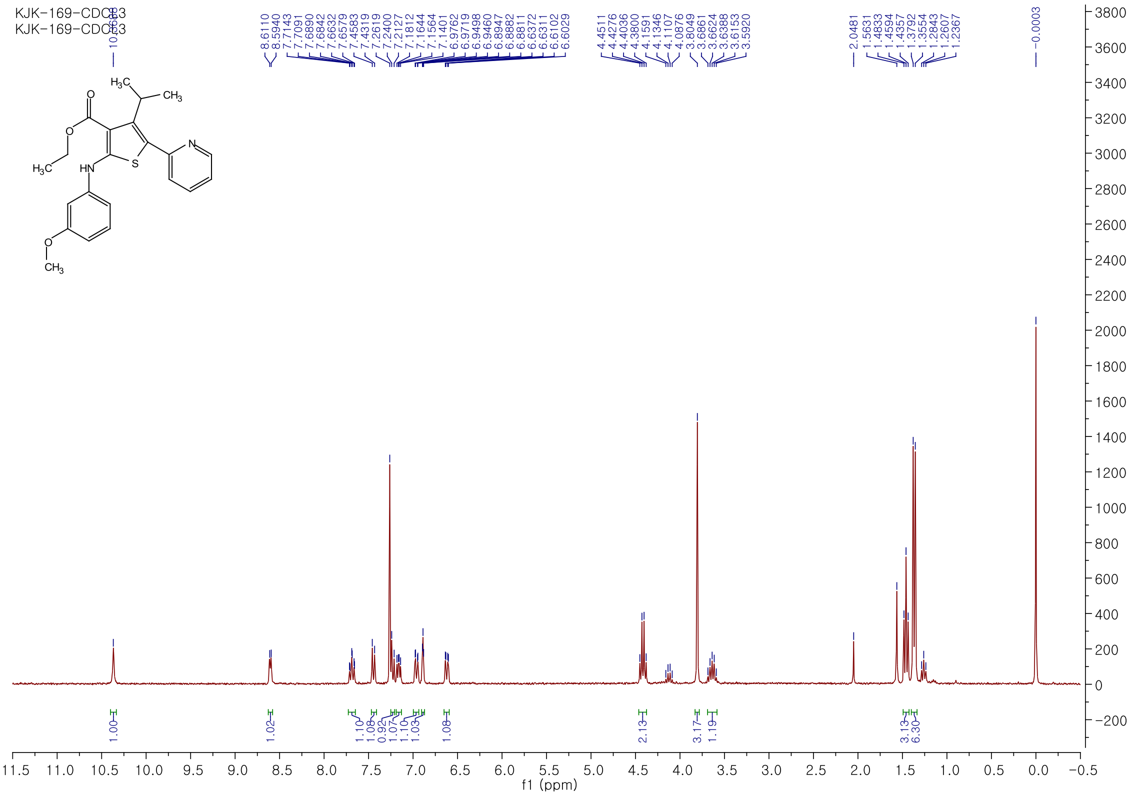

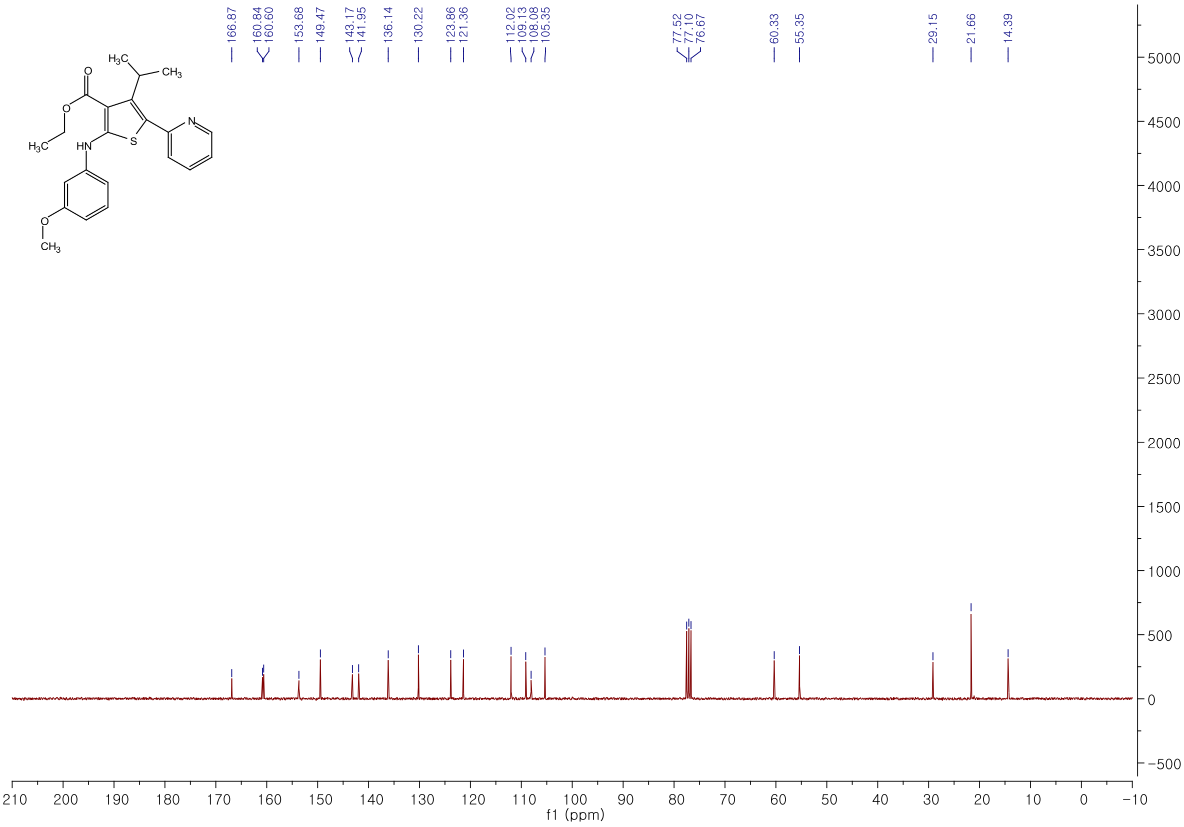

^{1}H and ^{13}C NMR of compound **8ak**

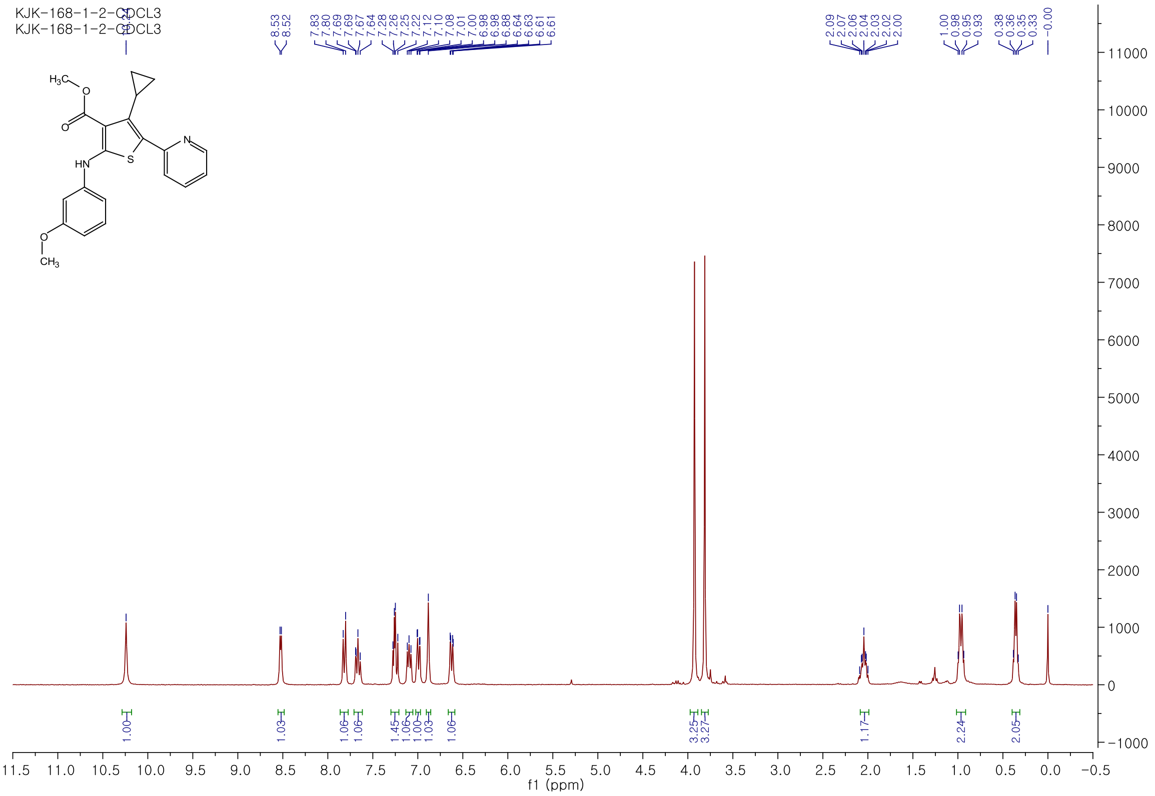

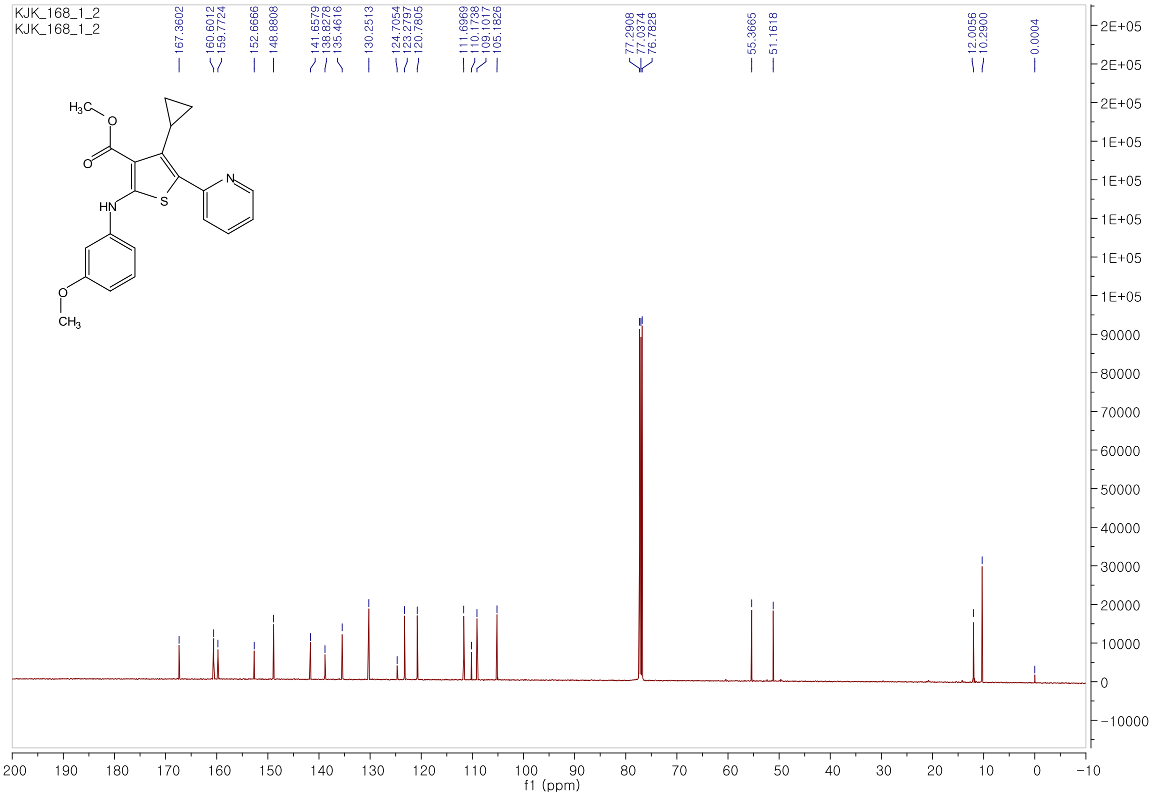

^{1}H and ^{13}C NMR of compound **8al**

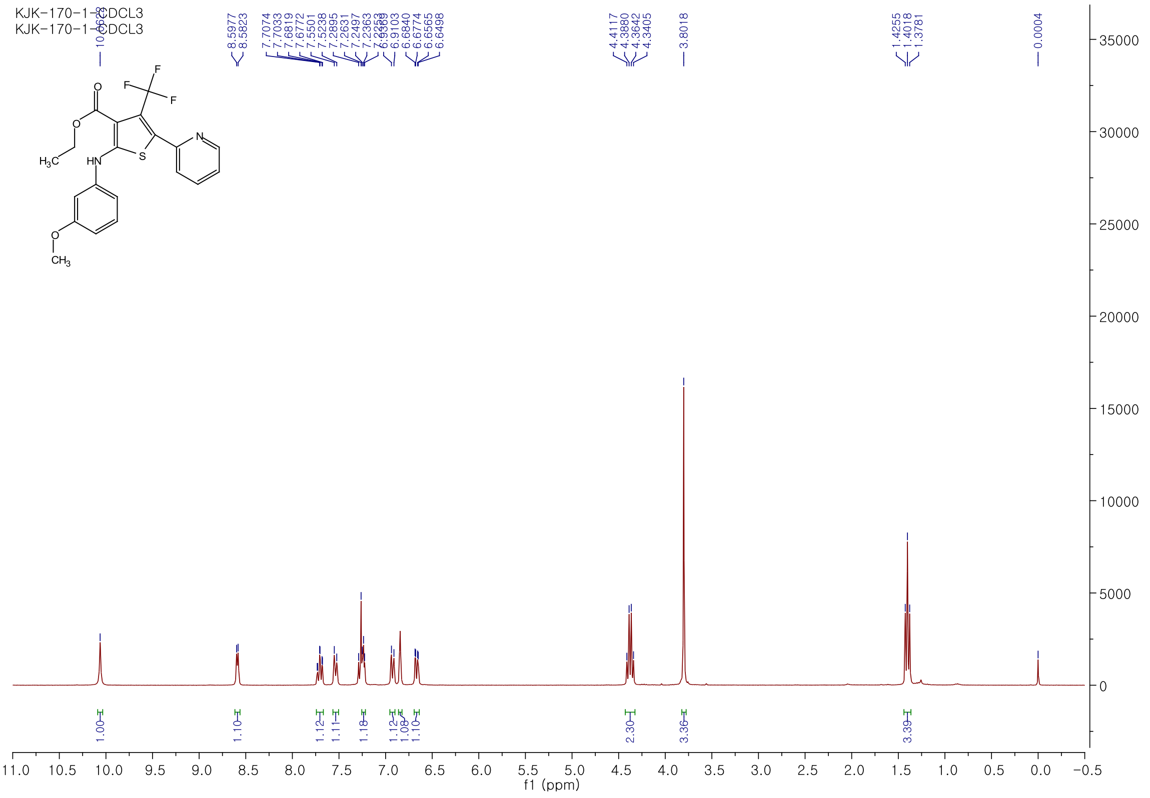

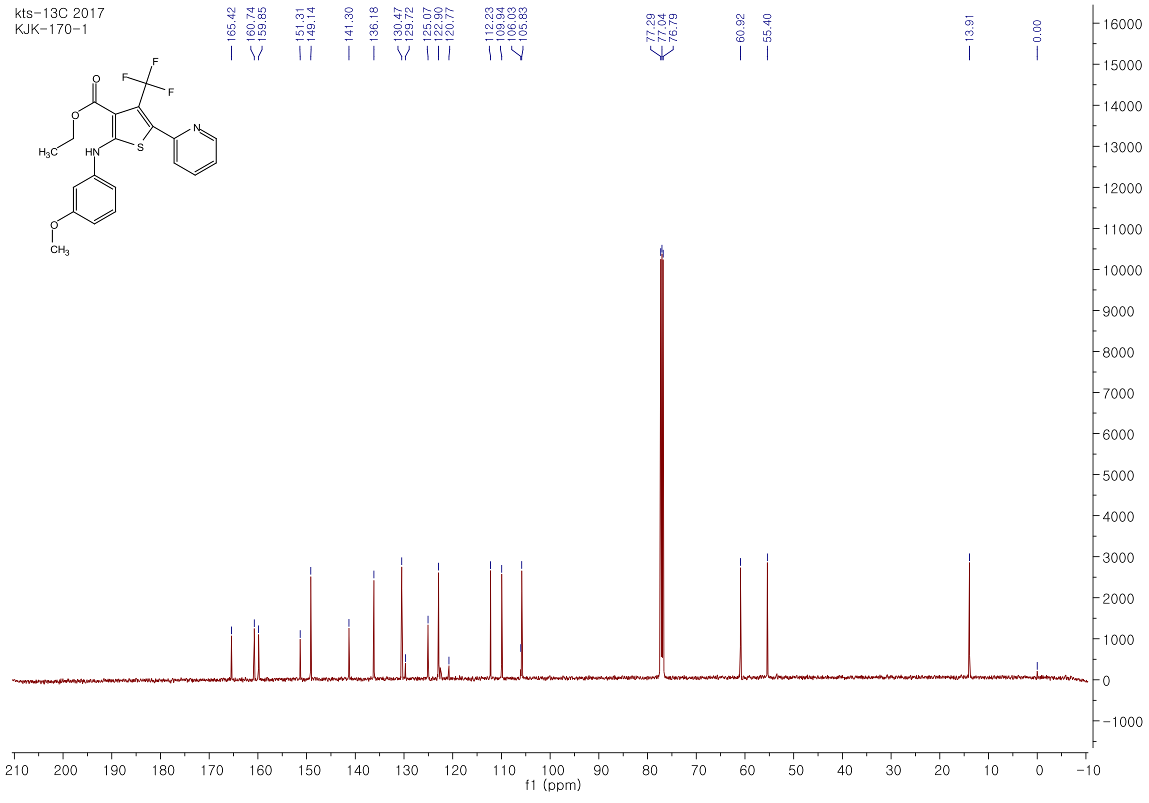

^{1}H and ^{13}C NMR of compound **8am**

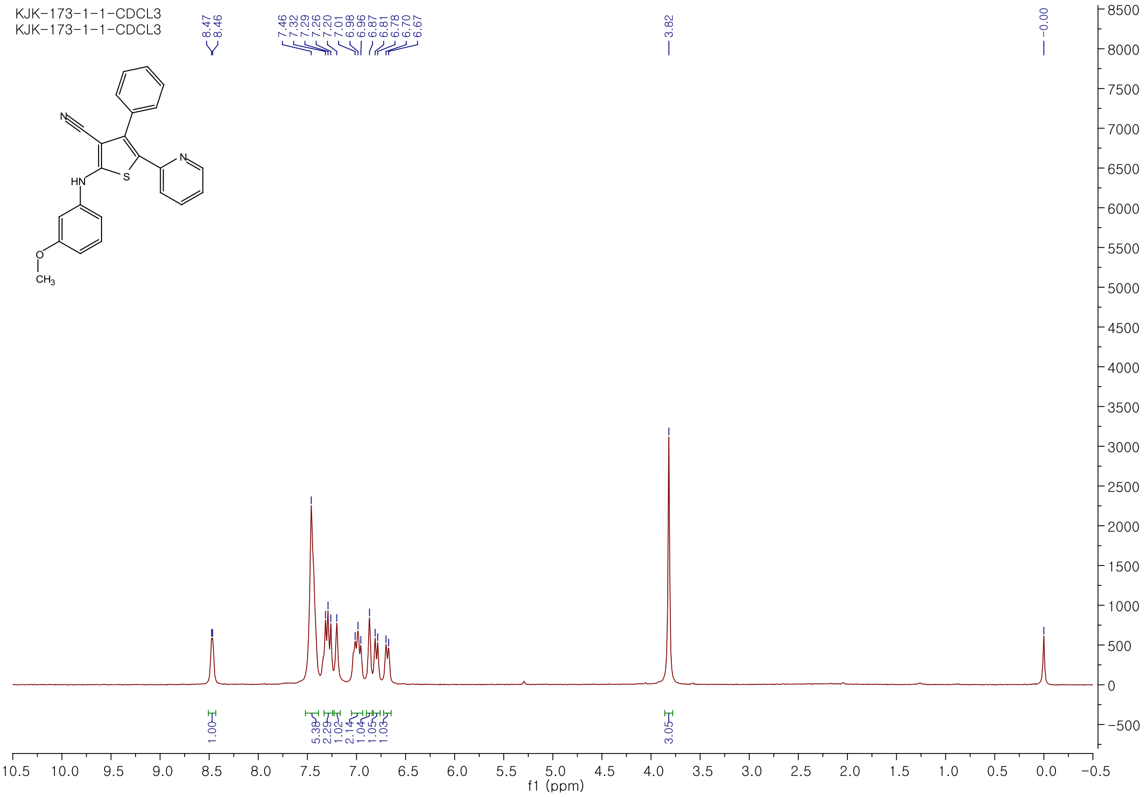

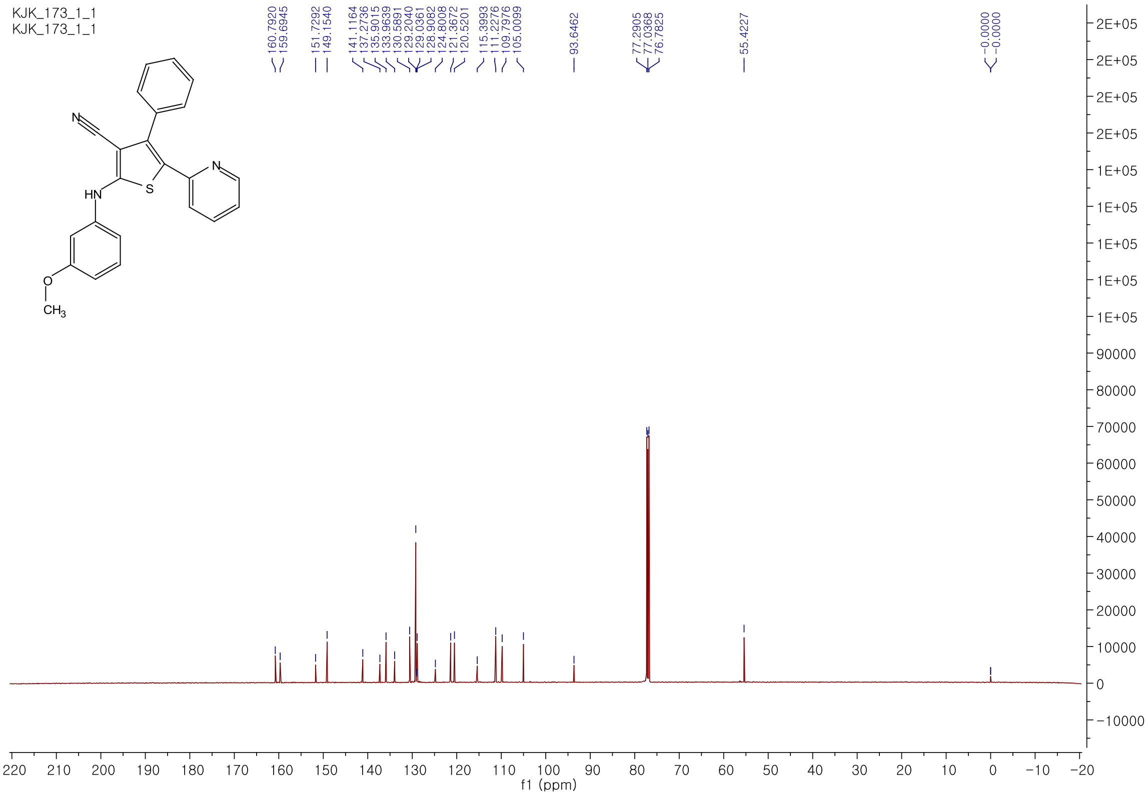

^{1}H and ^{13}C NMR of compound **8an**

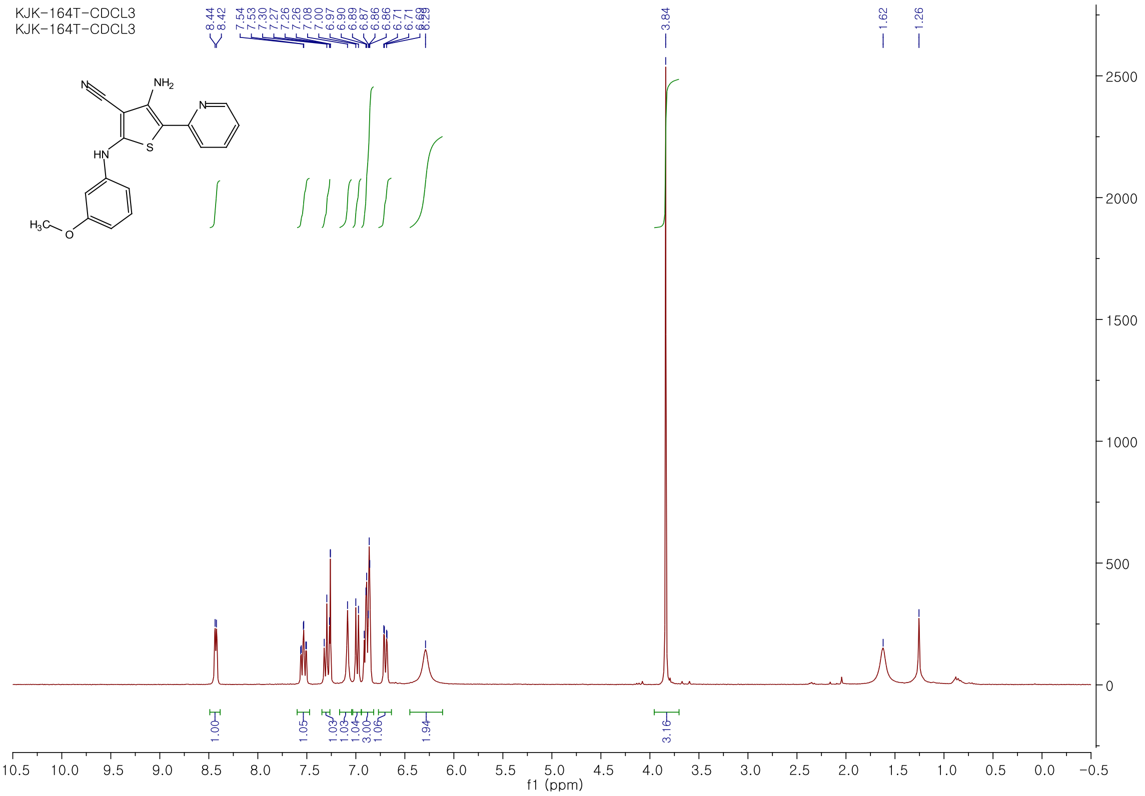

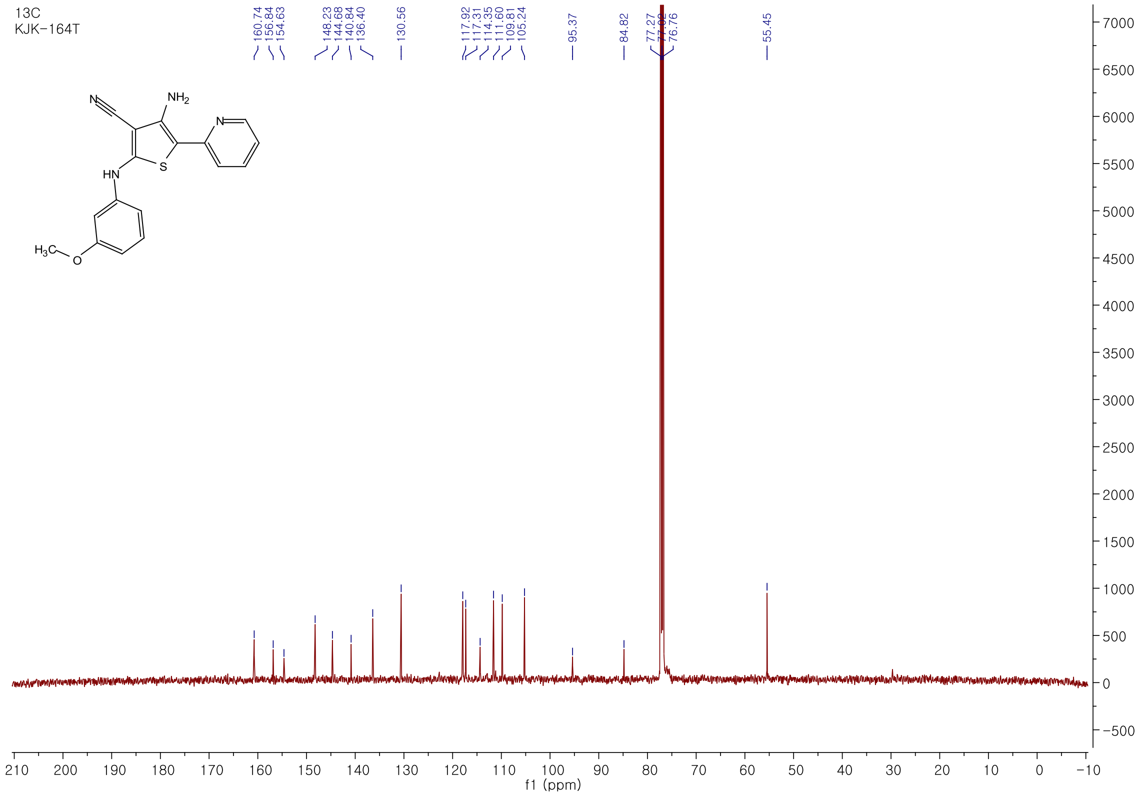

^{1}H and ^{13}C NMR of compound **7ao**

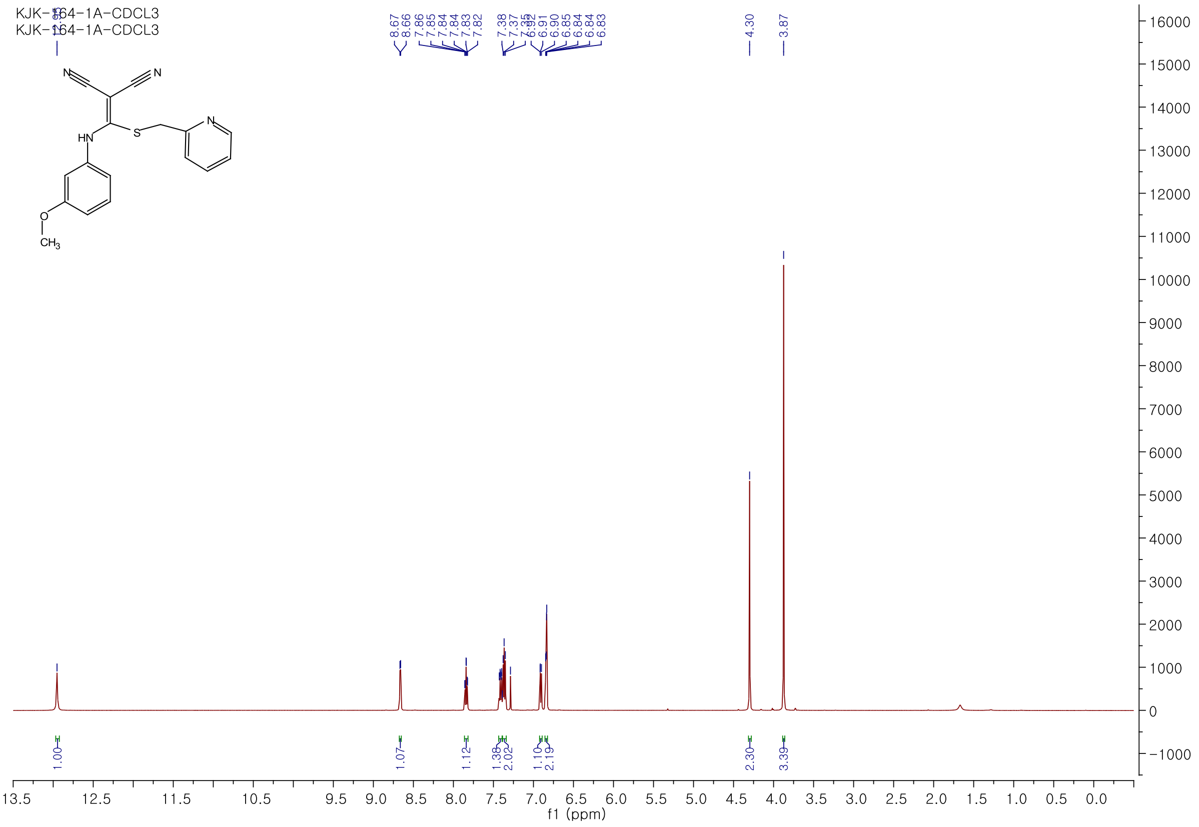

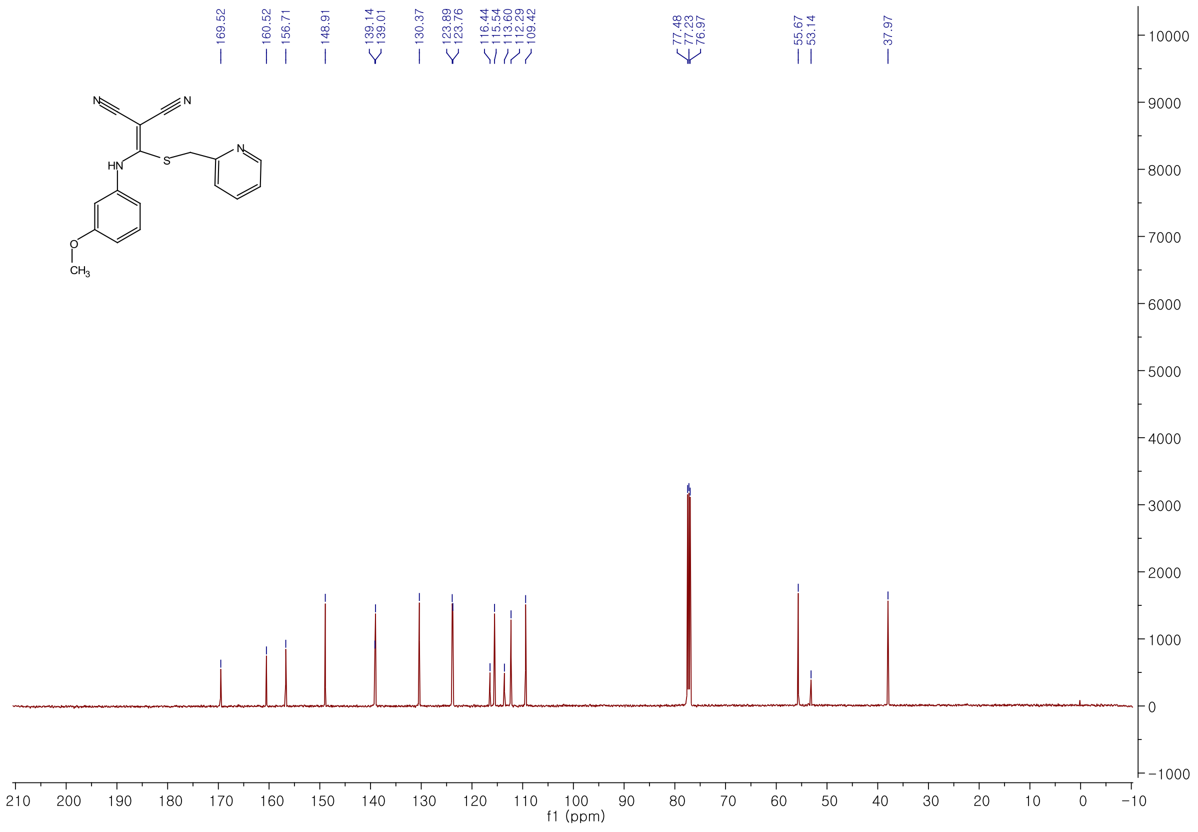

^{1}H and ^{13}C NMR of compound **8ao**

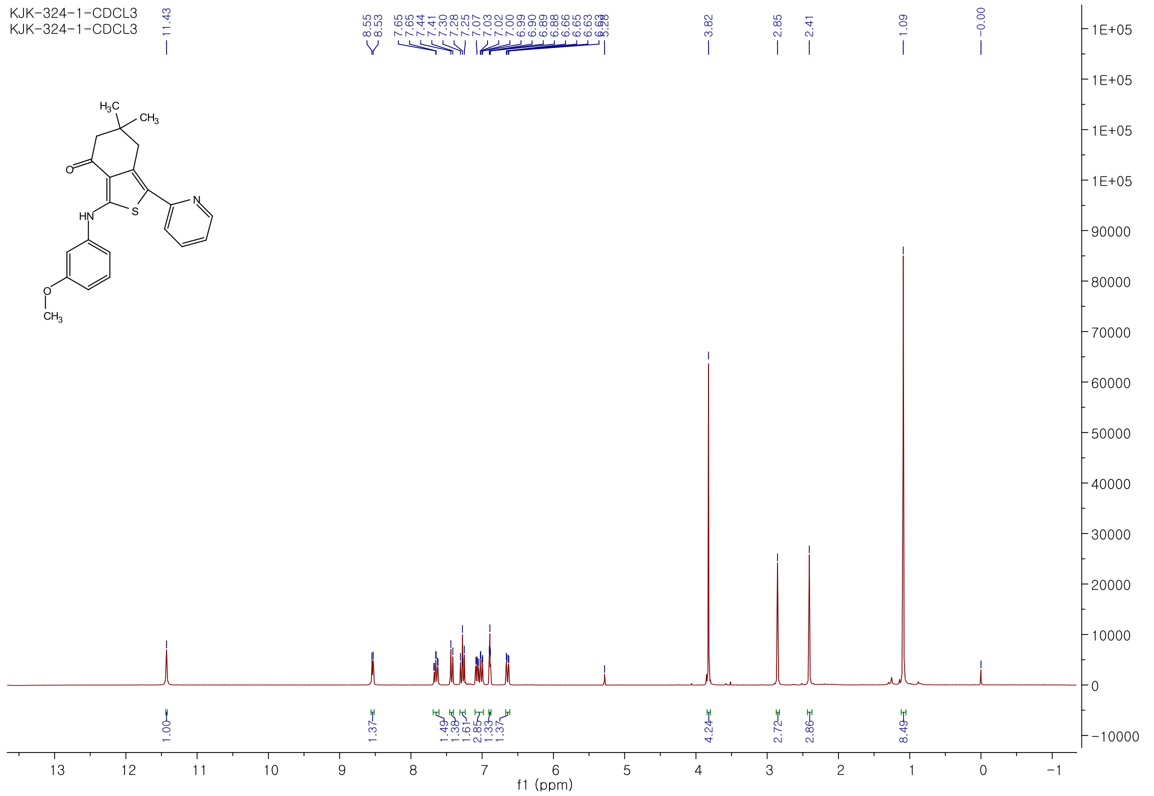

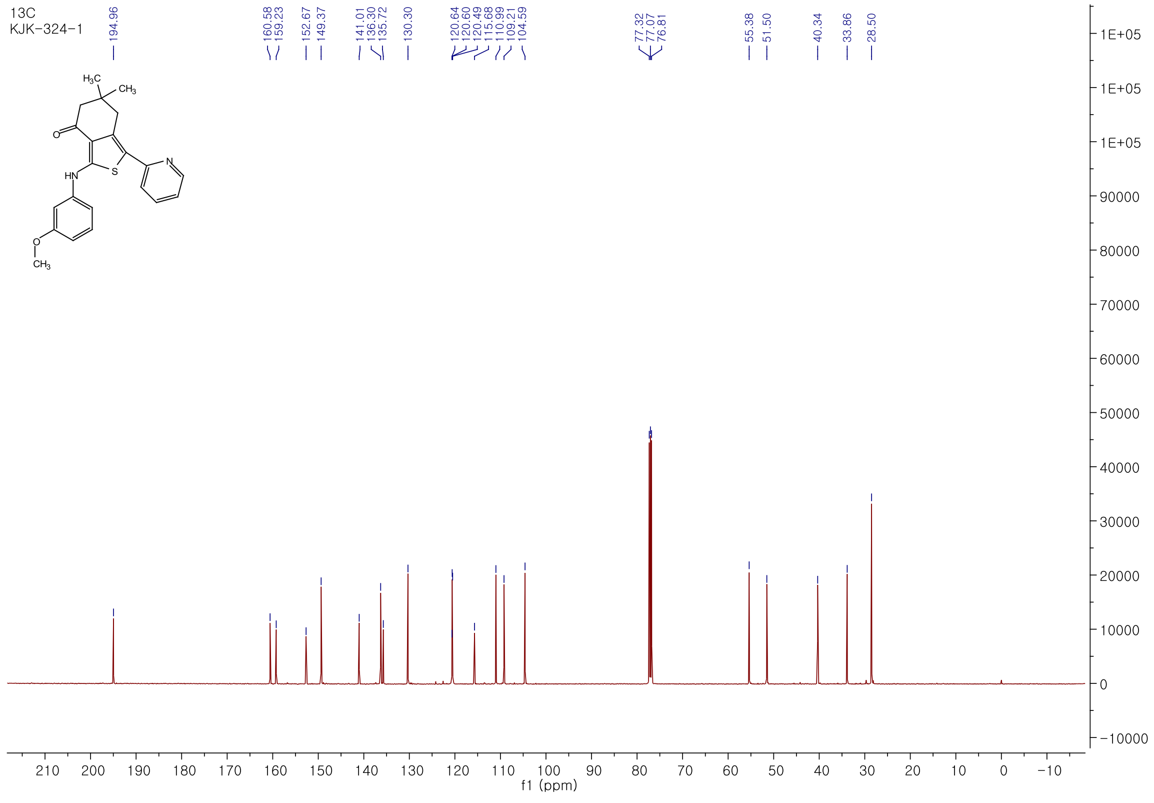

^{1}H and ^{13}C NMR of compound **8c**

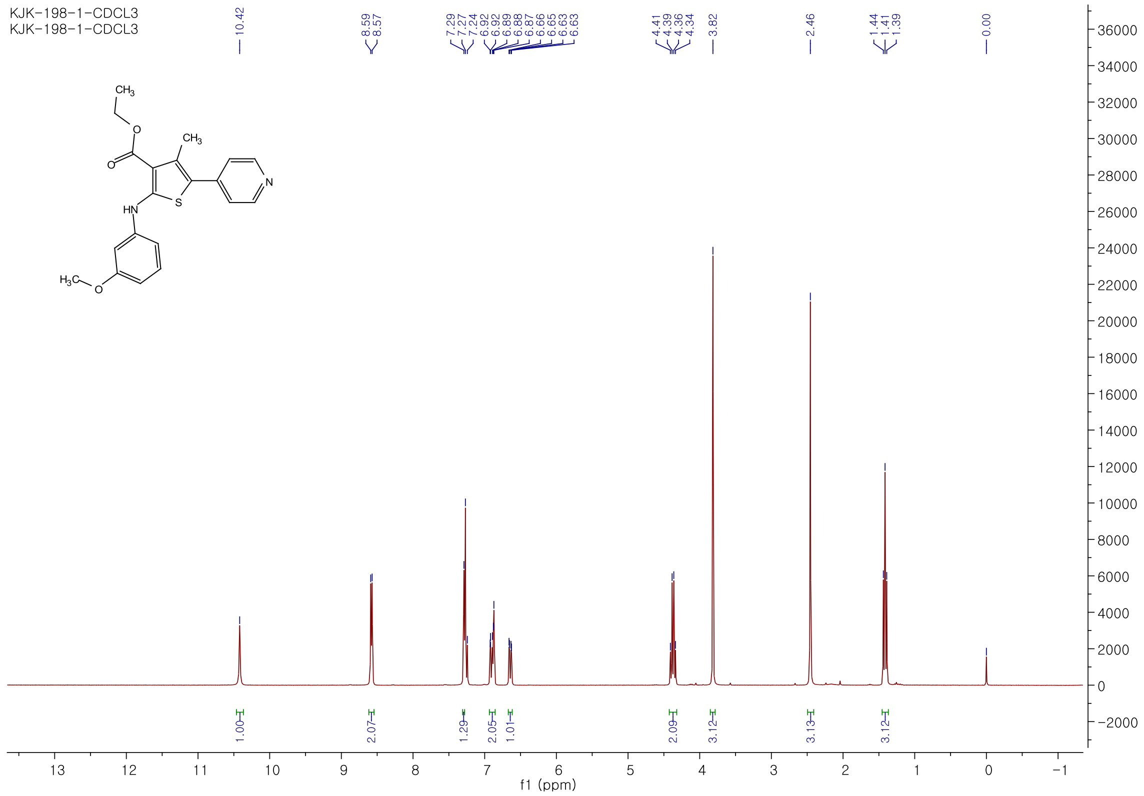

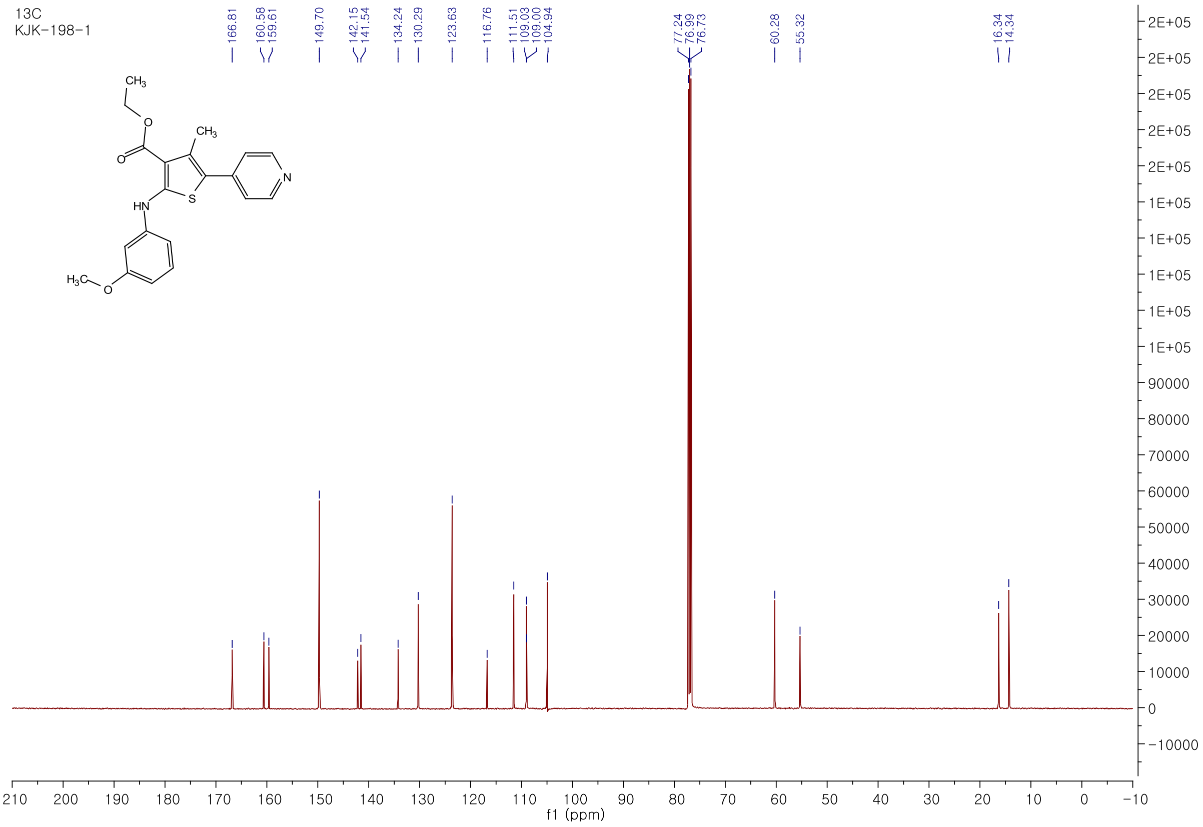

^{1}H and ^{13}C NMR of compound **8d**

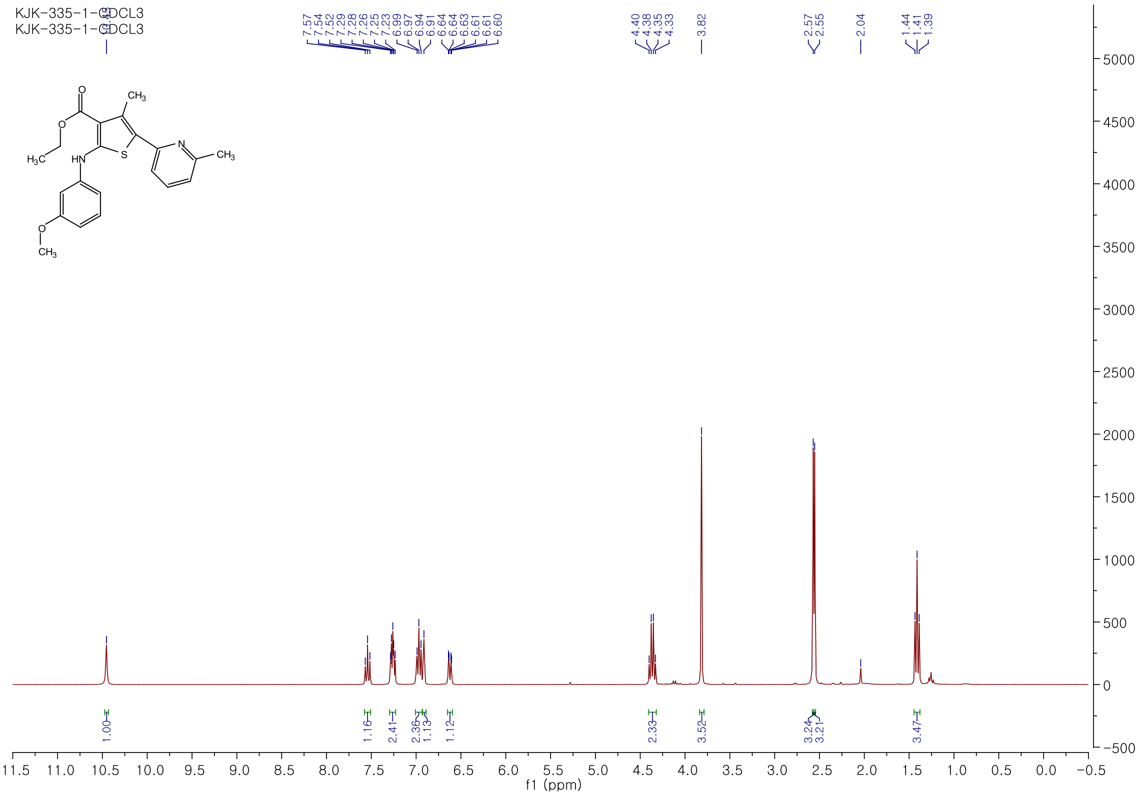

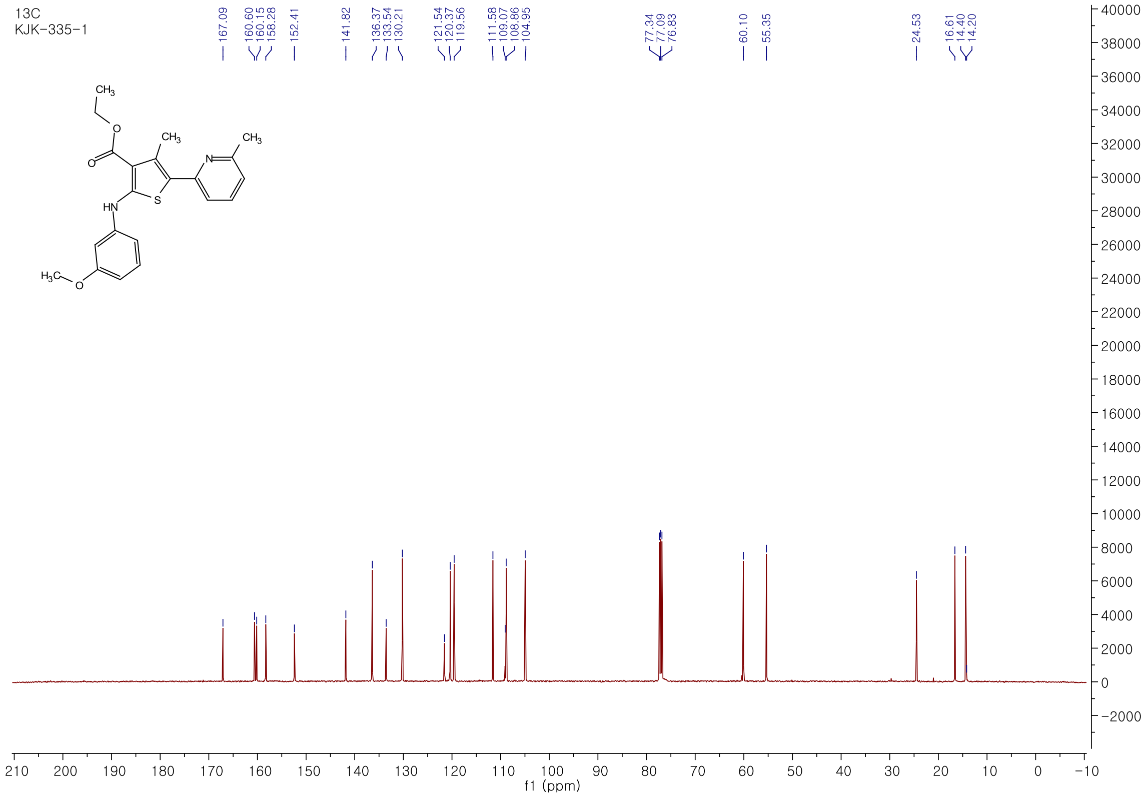

^{1}H and ^{13}C NMR of compound **8f**

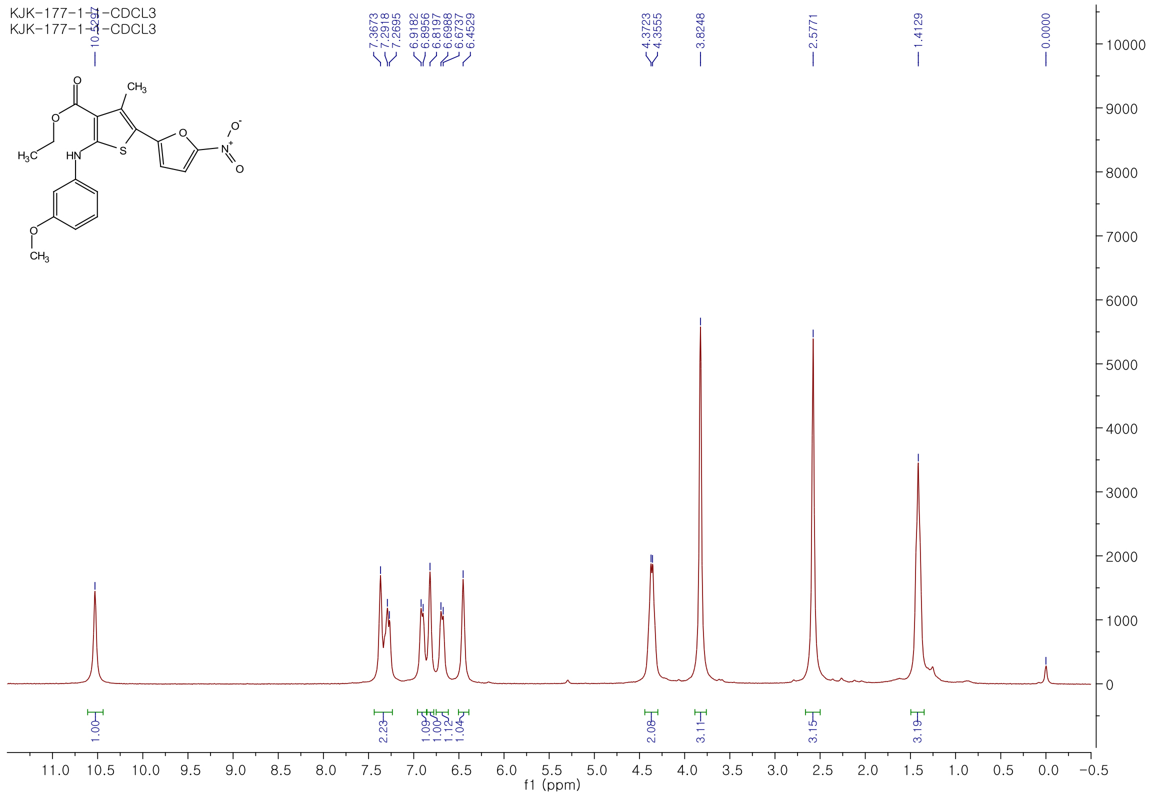

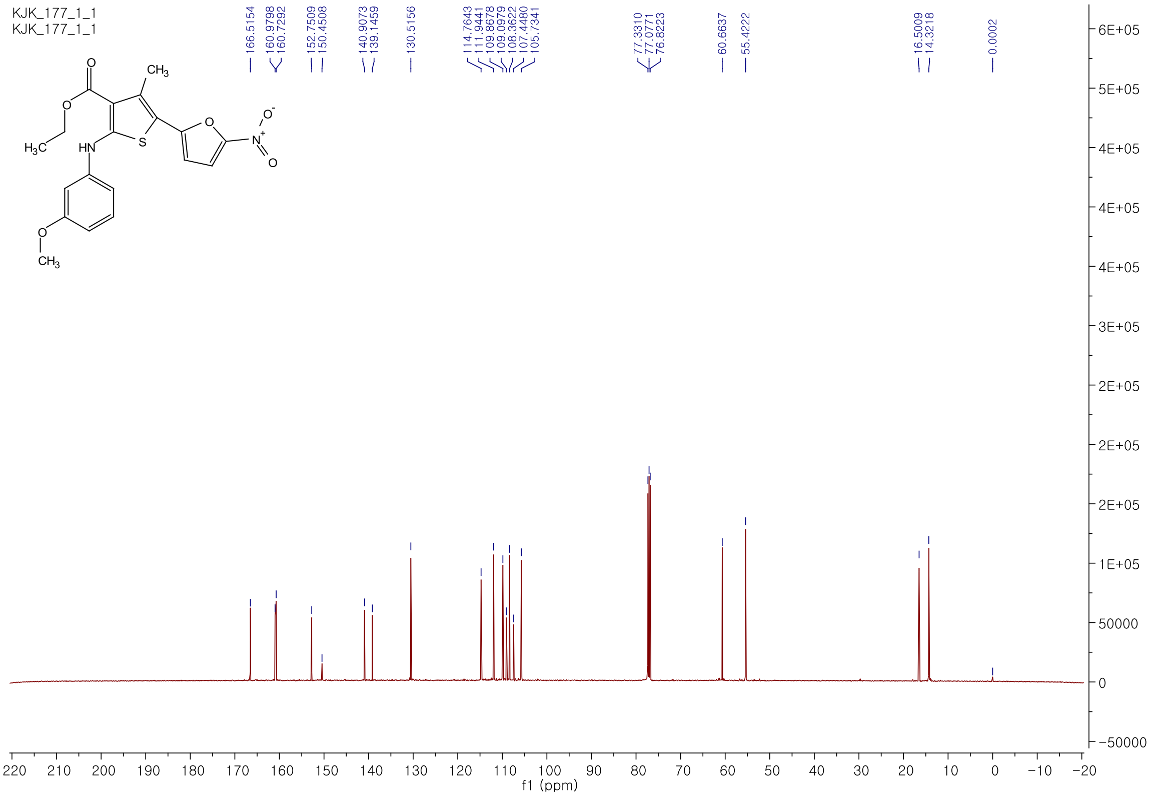

^{1}H and ^{13}C NMR of compound **8g**

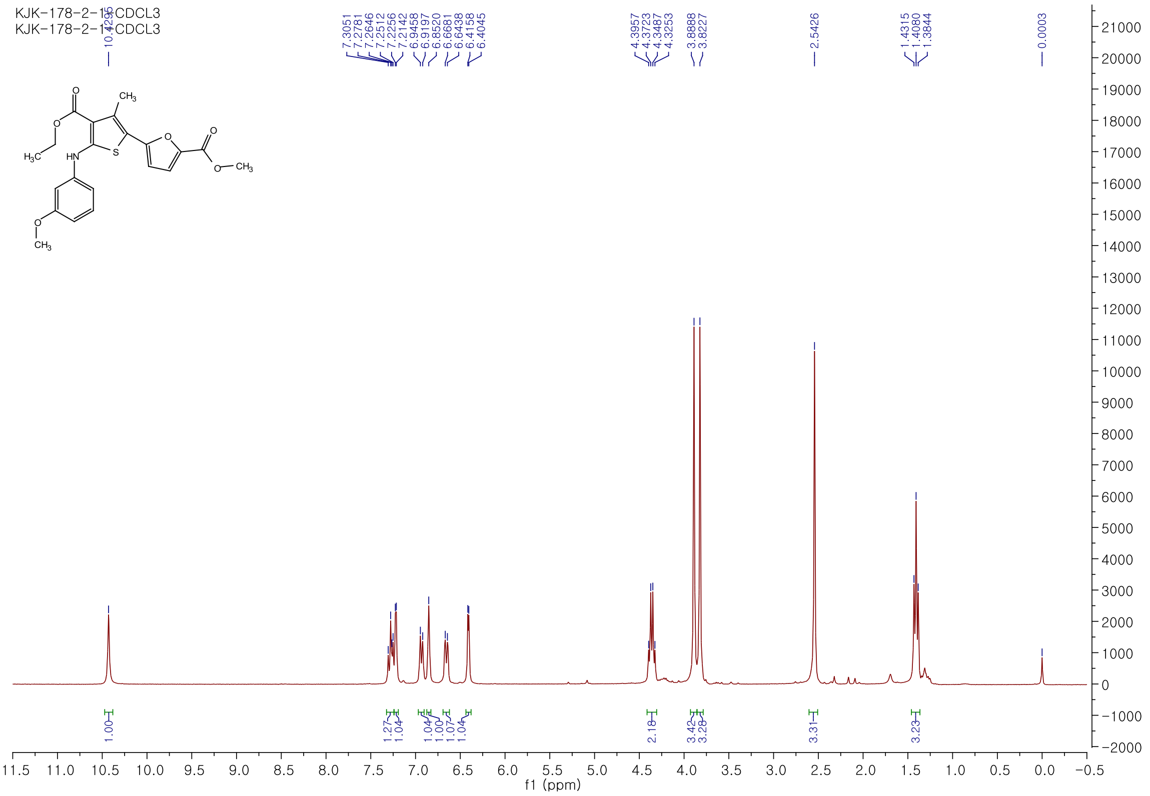

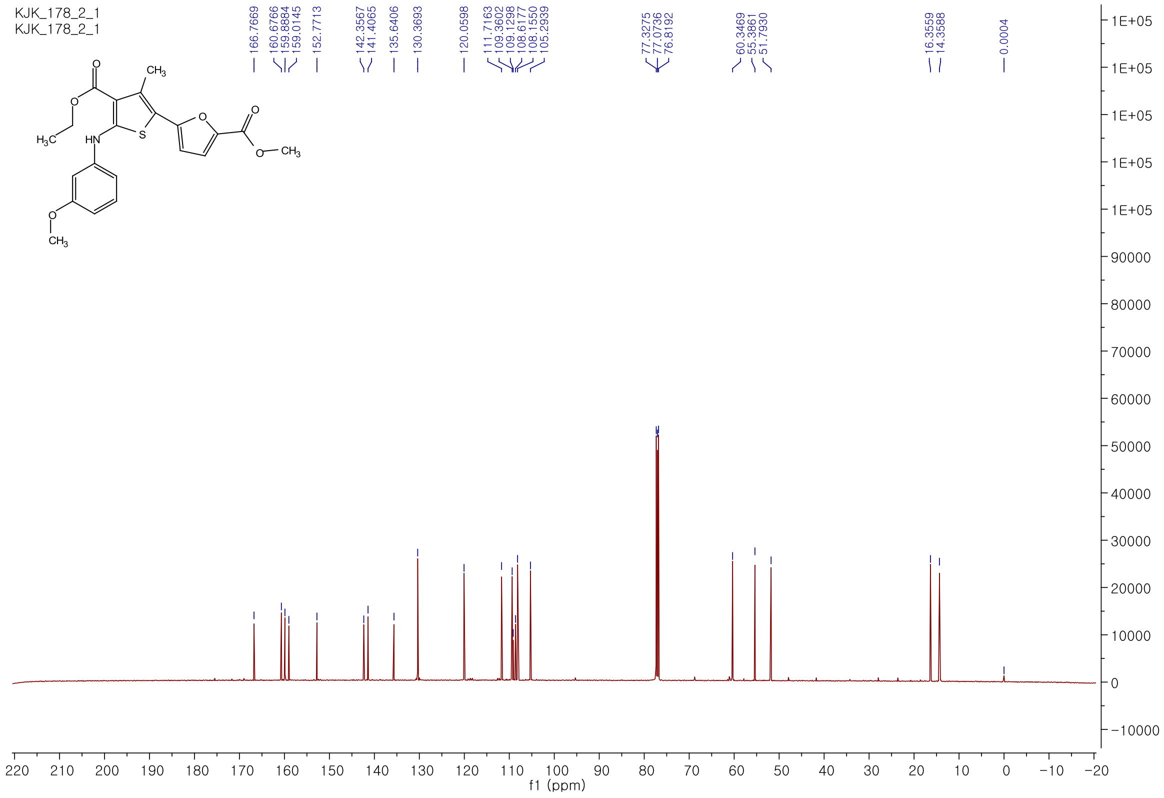

^{1}H and ^{13}C NMR of compound **8i**

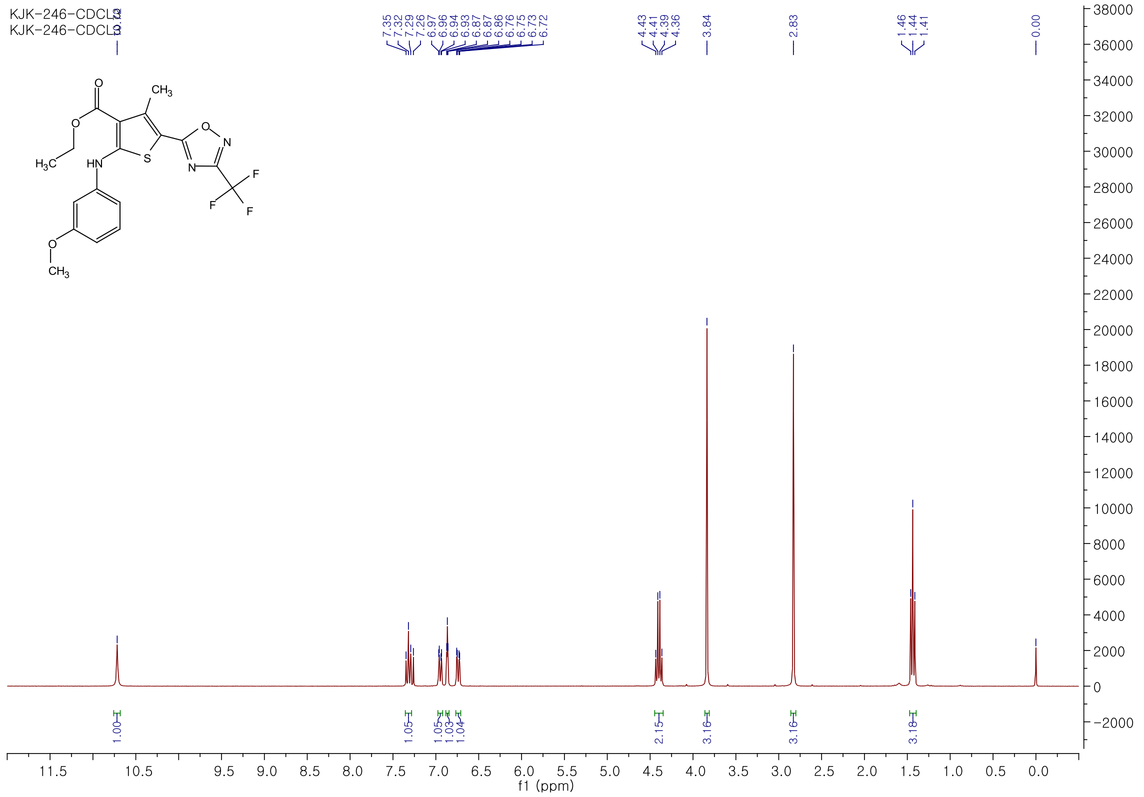

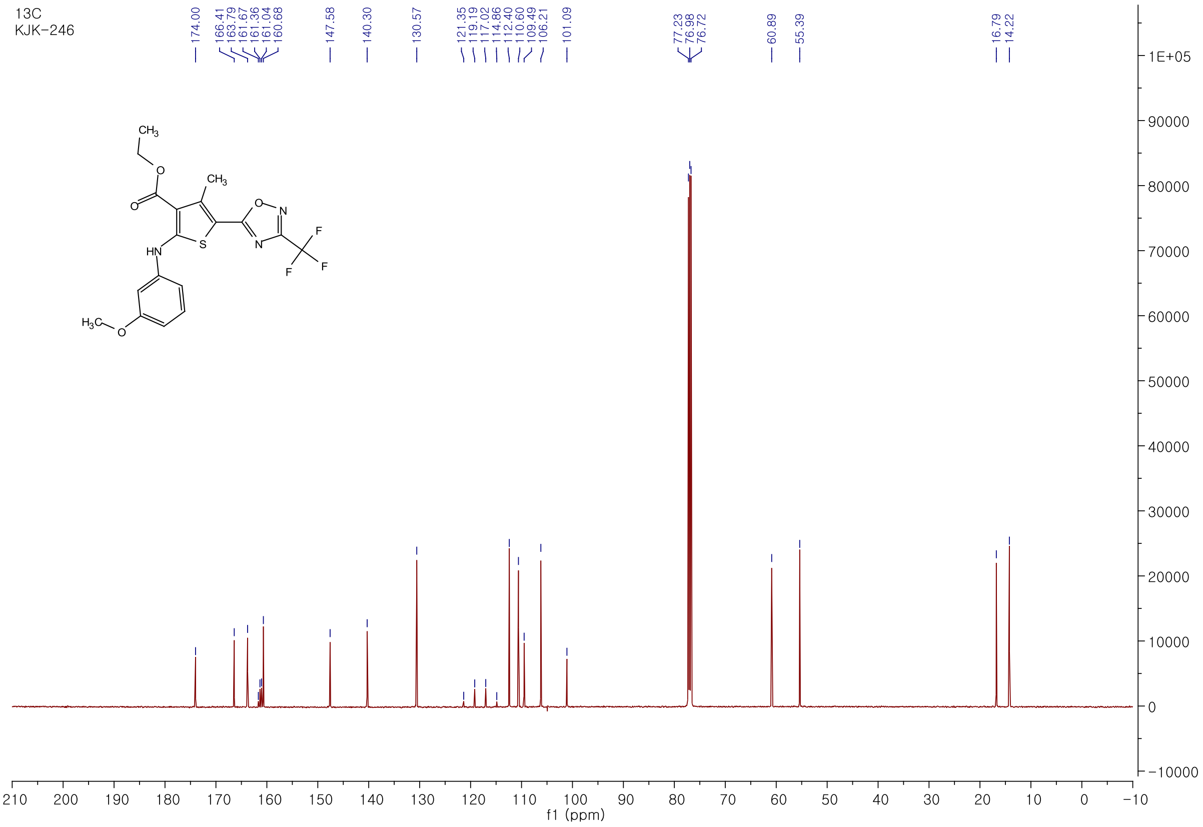

^{1}H and ^{13}C NMR of compound **8k**

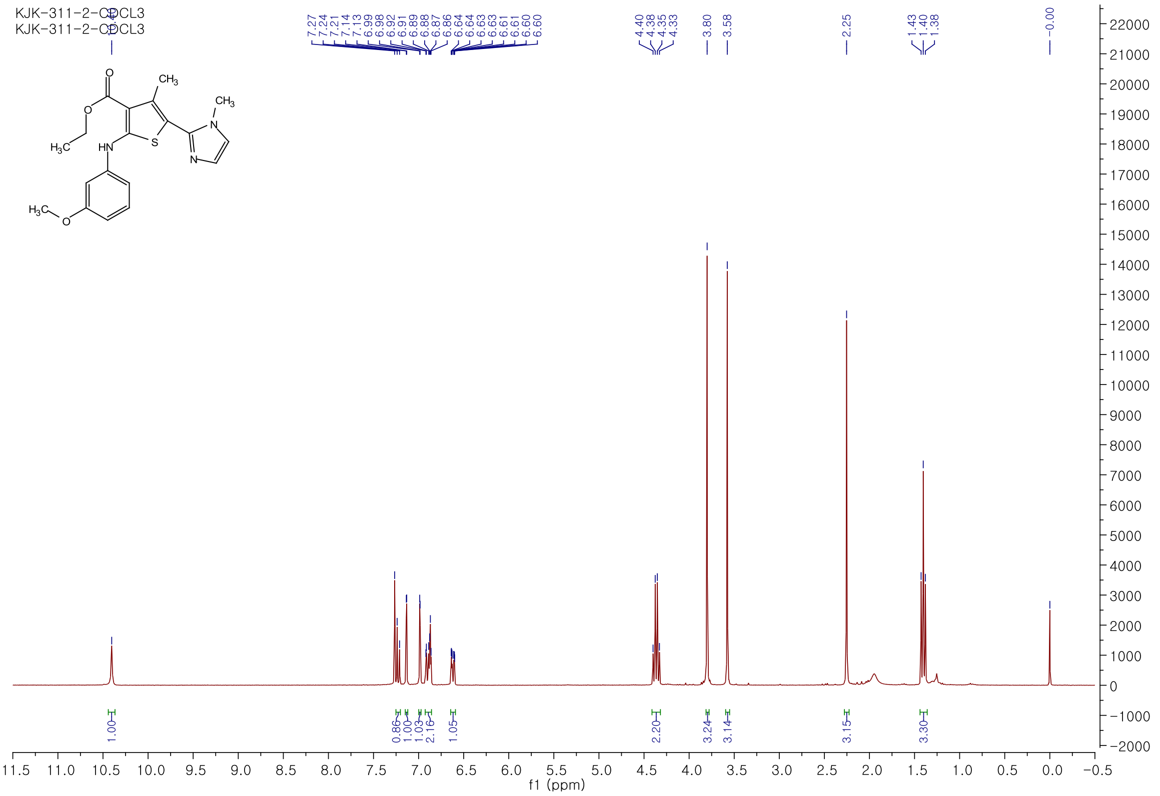

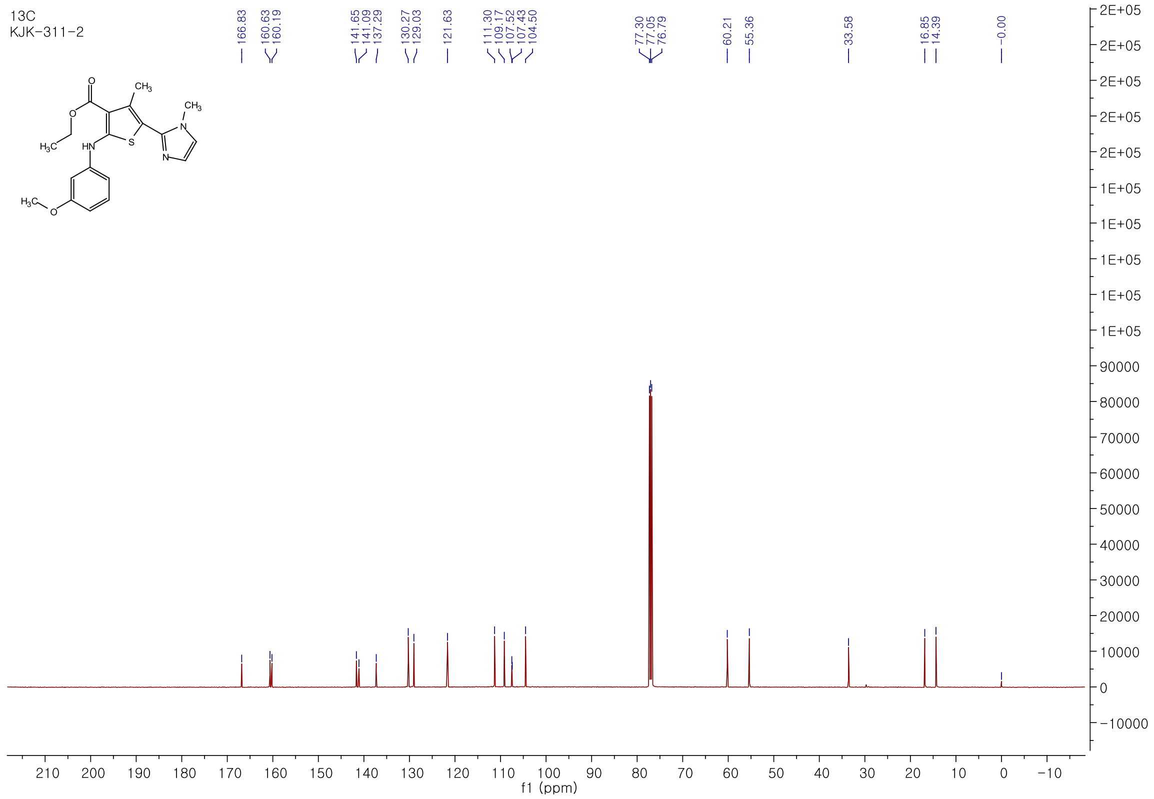

^{1}H and ^{13}C NMR of compound **8p**

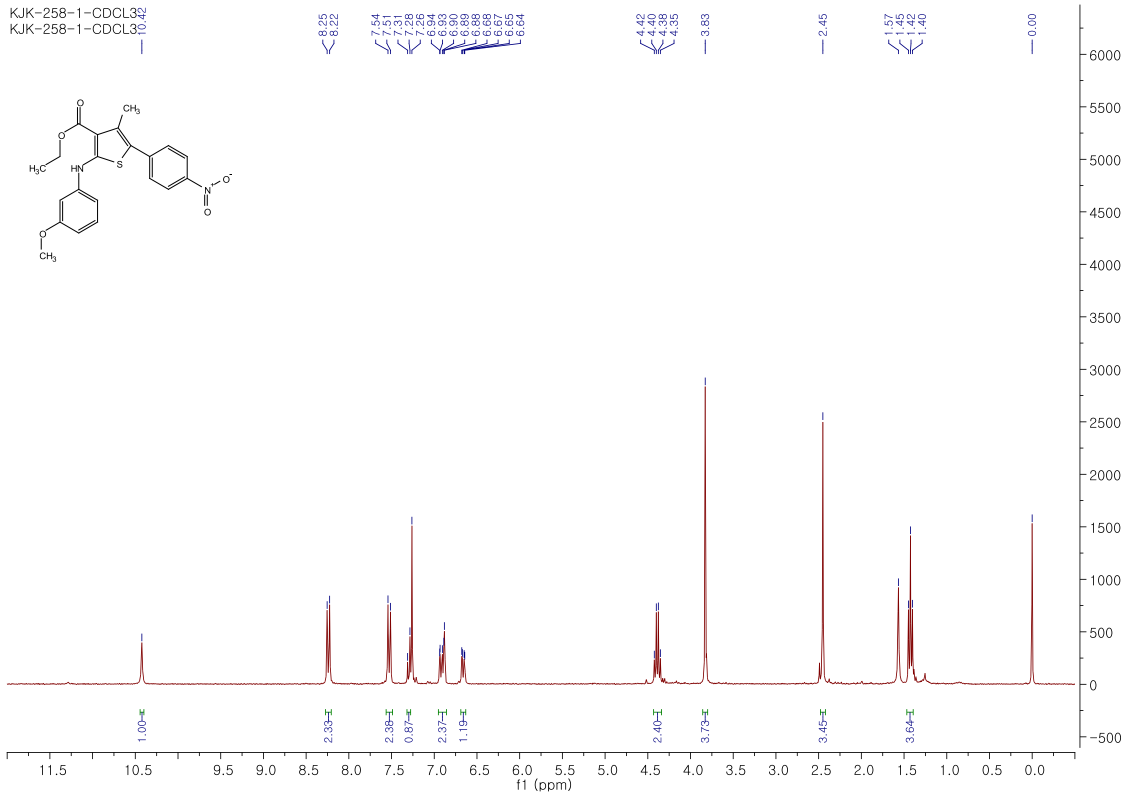

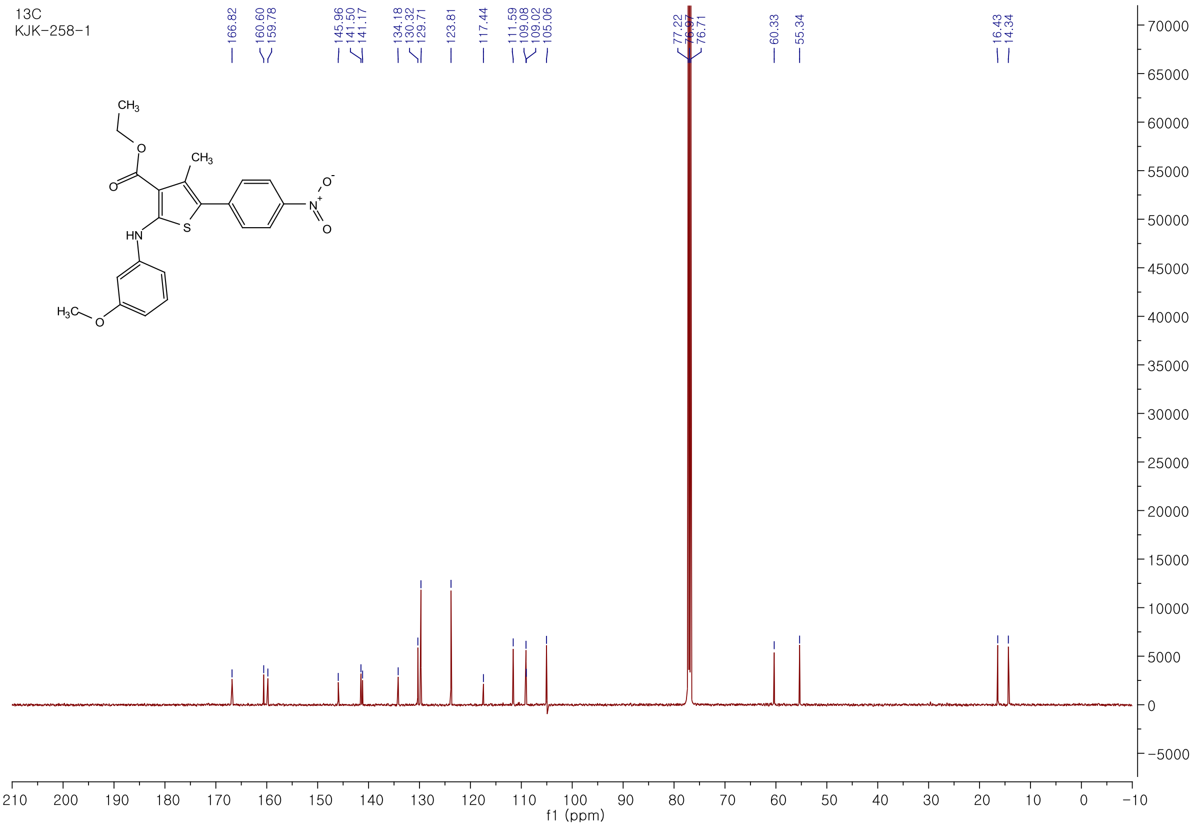

^{1}H and ^{13}C NMR of compound **9a**

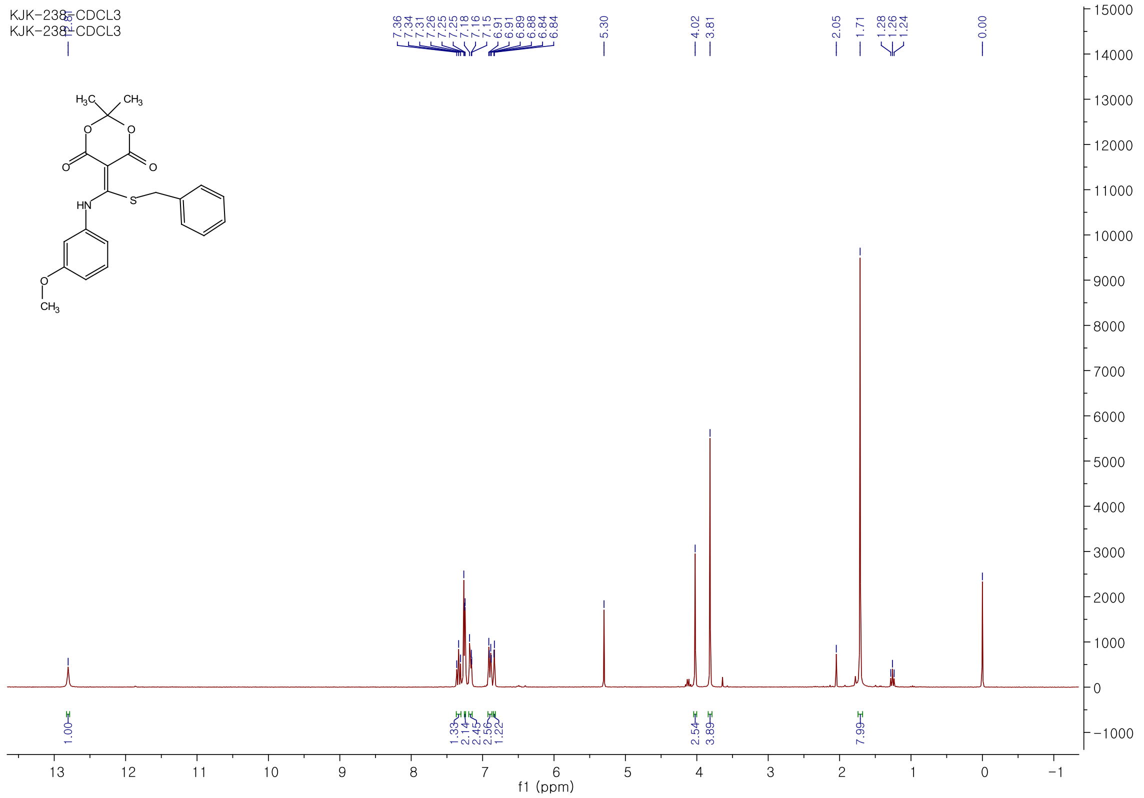

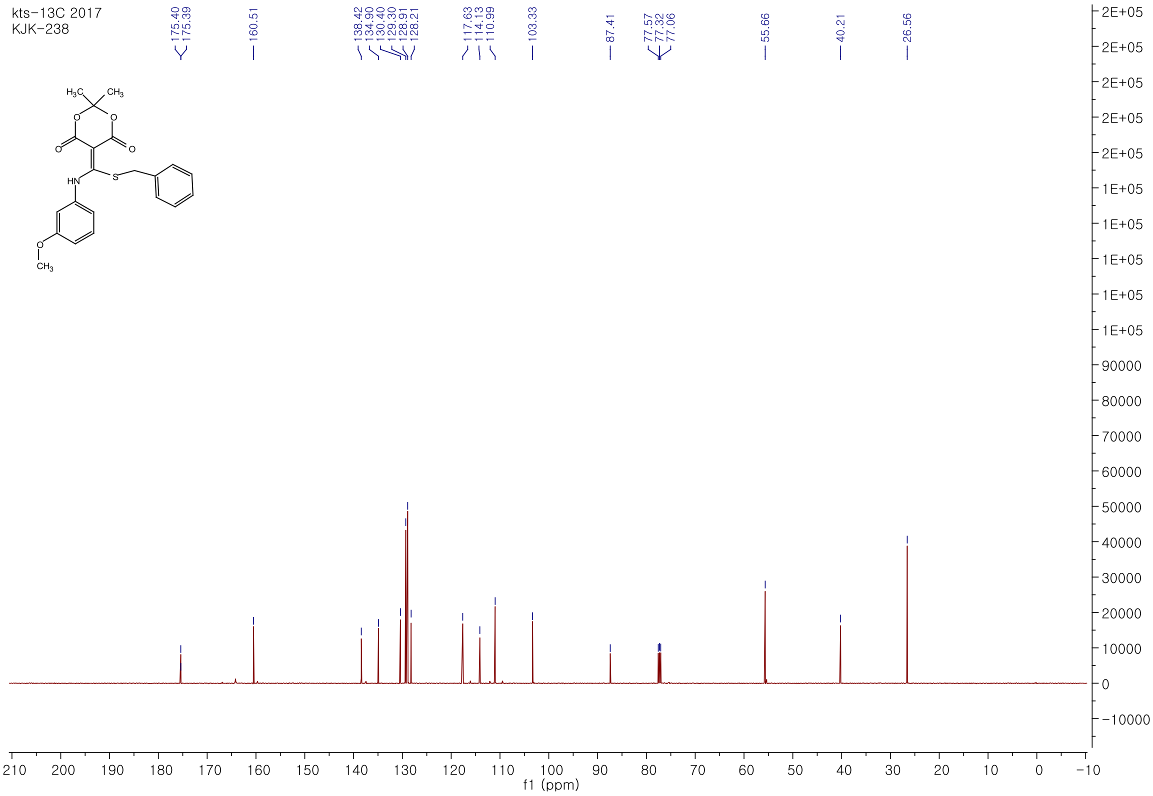

^{1}H and ^{13}C NMR of compound **9b**

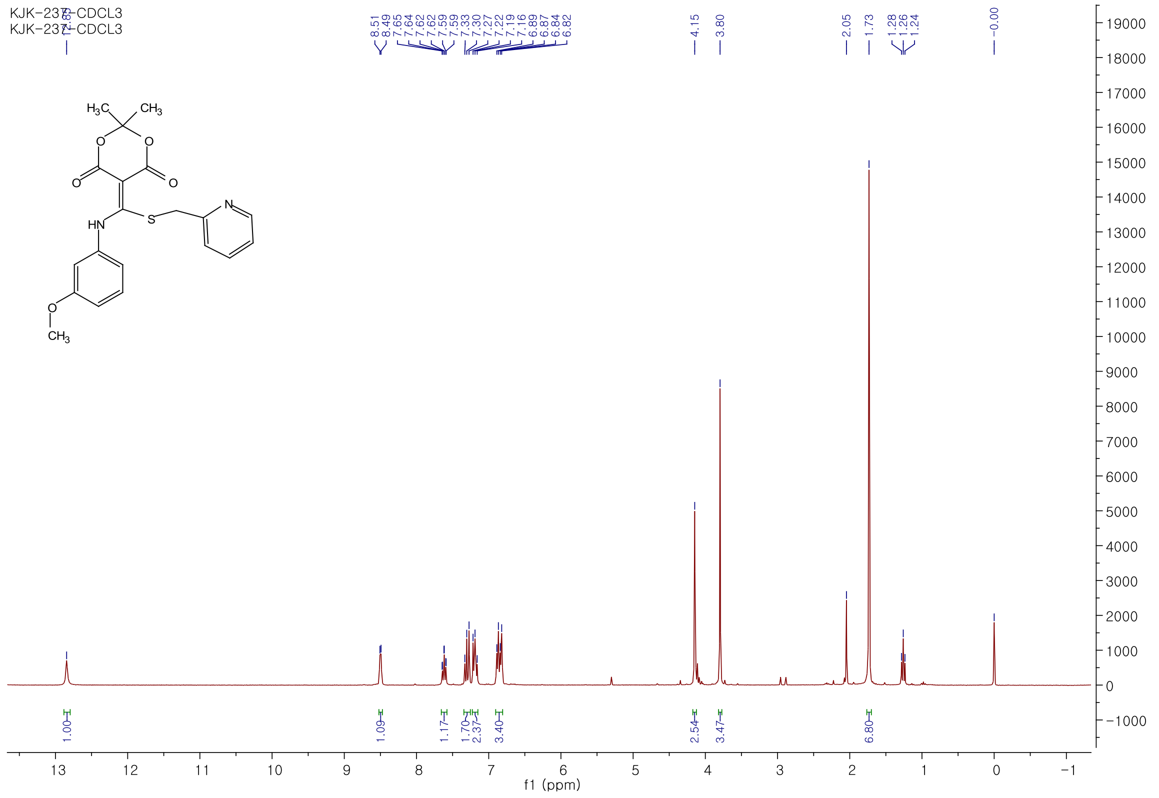

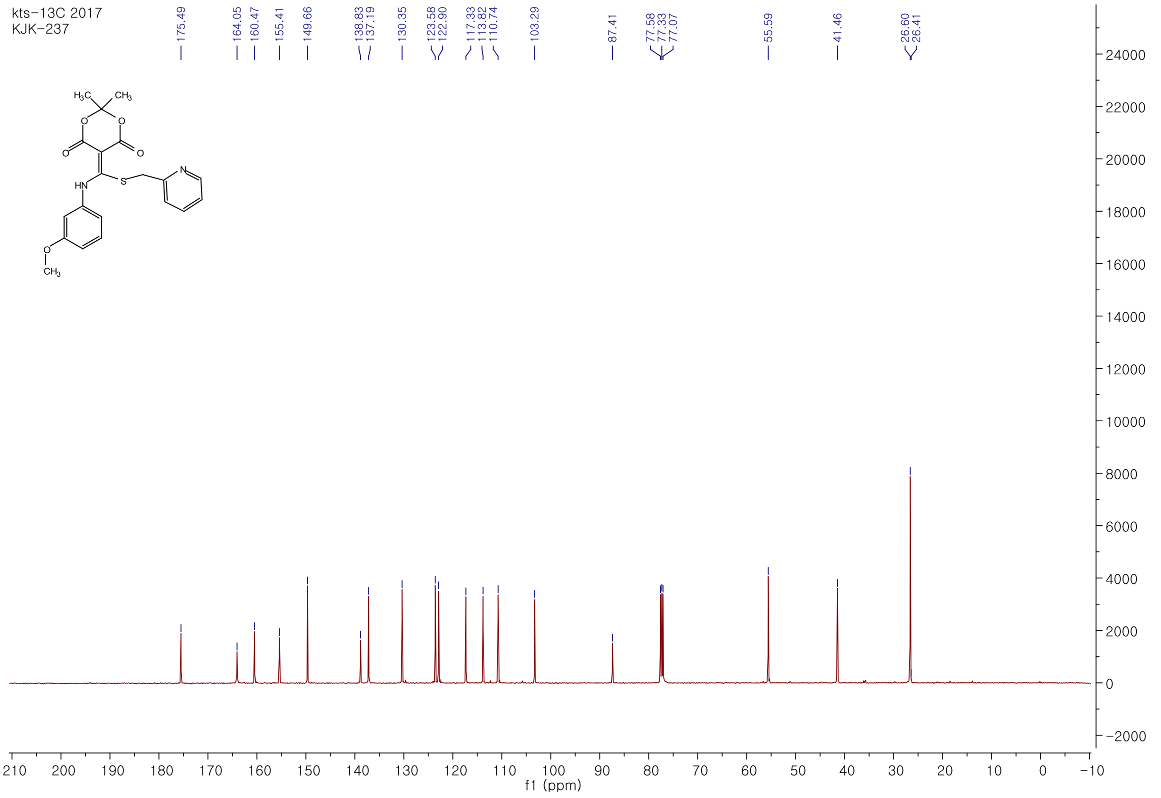

^{1}H and ^{13}C NMR of compound **9c**

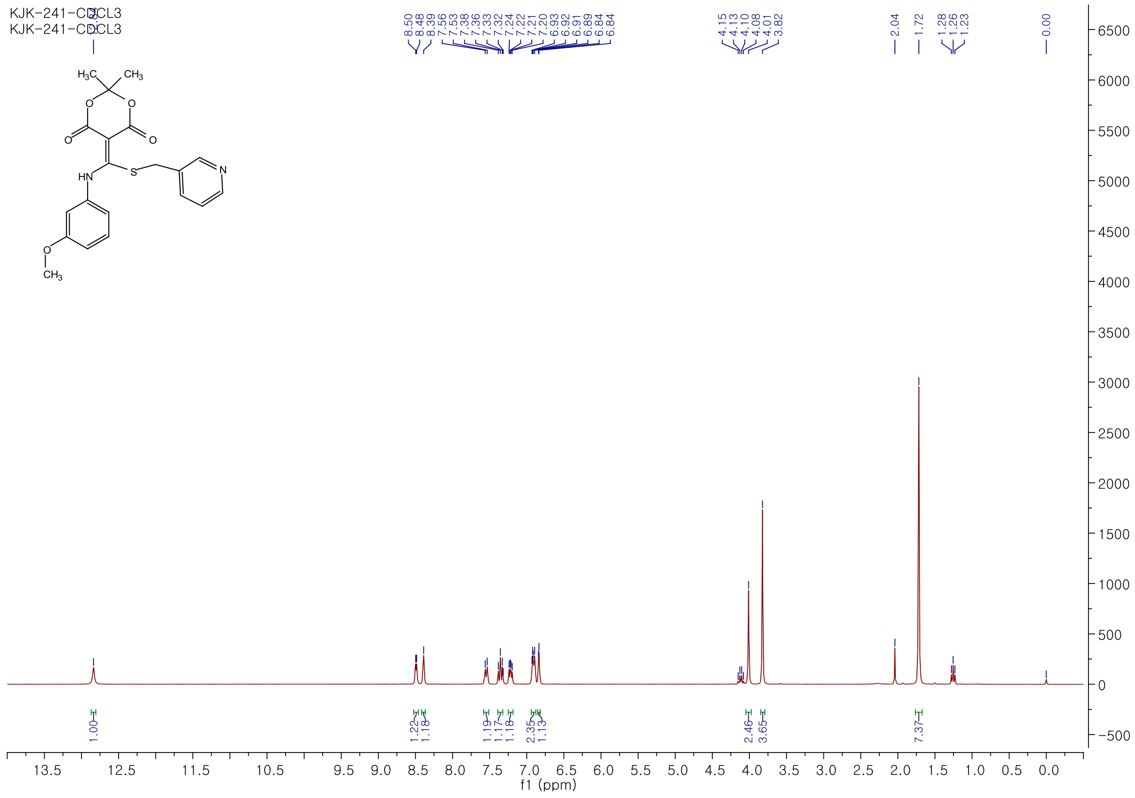

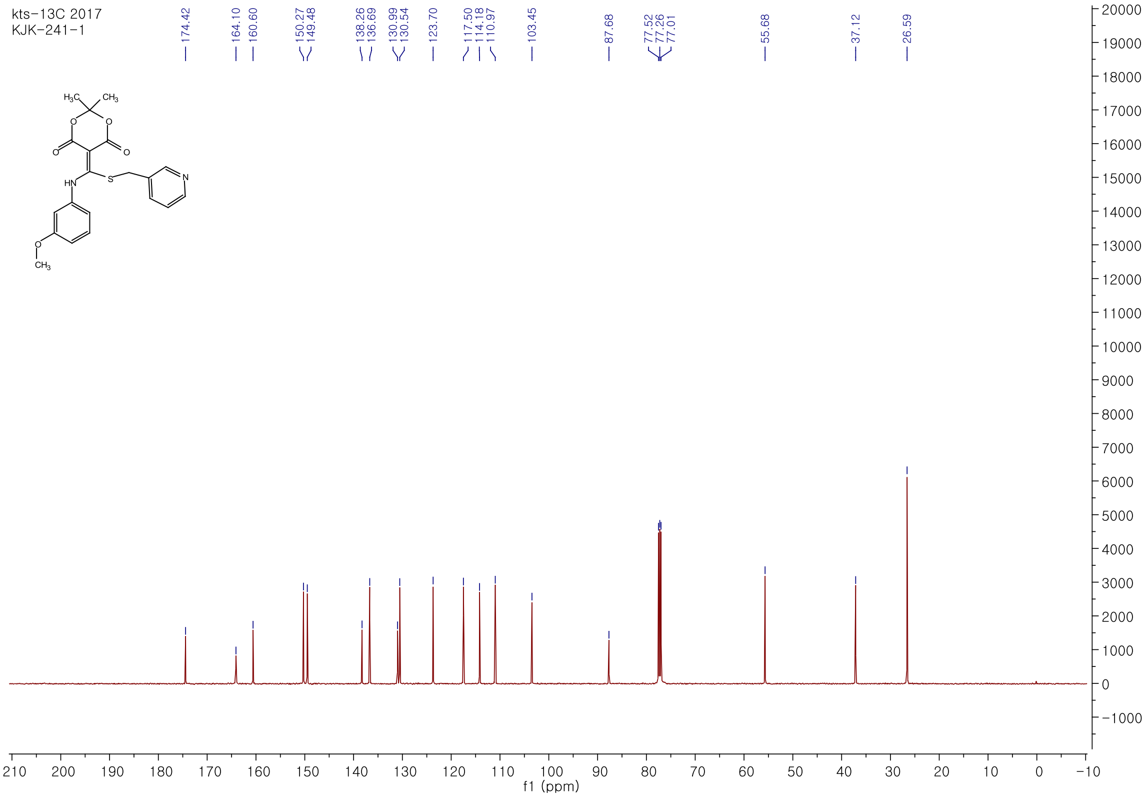

^1H and ^{13}C NMR of compound **9ba**

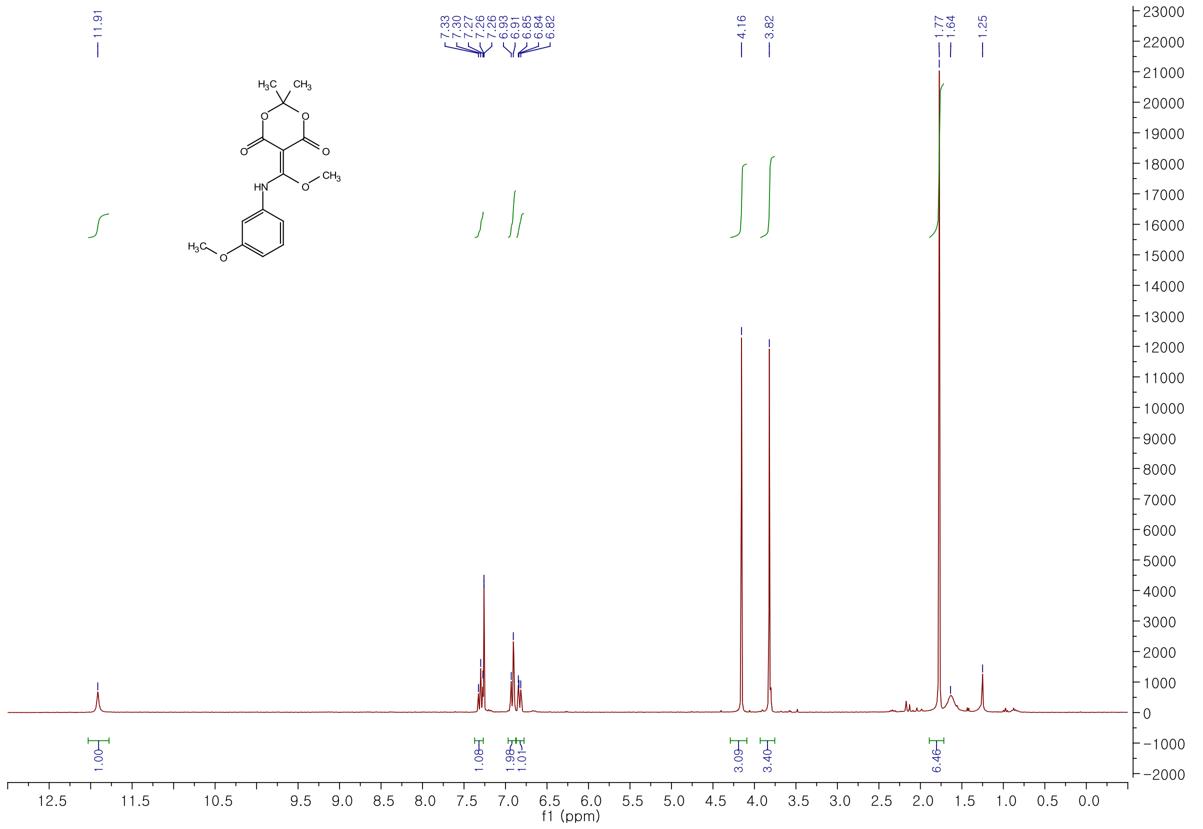

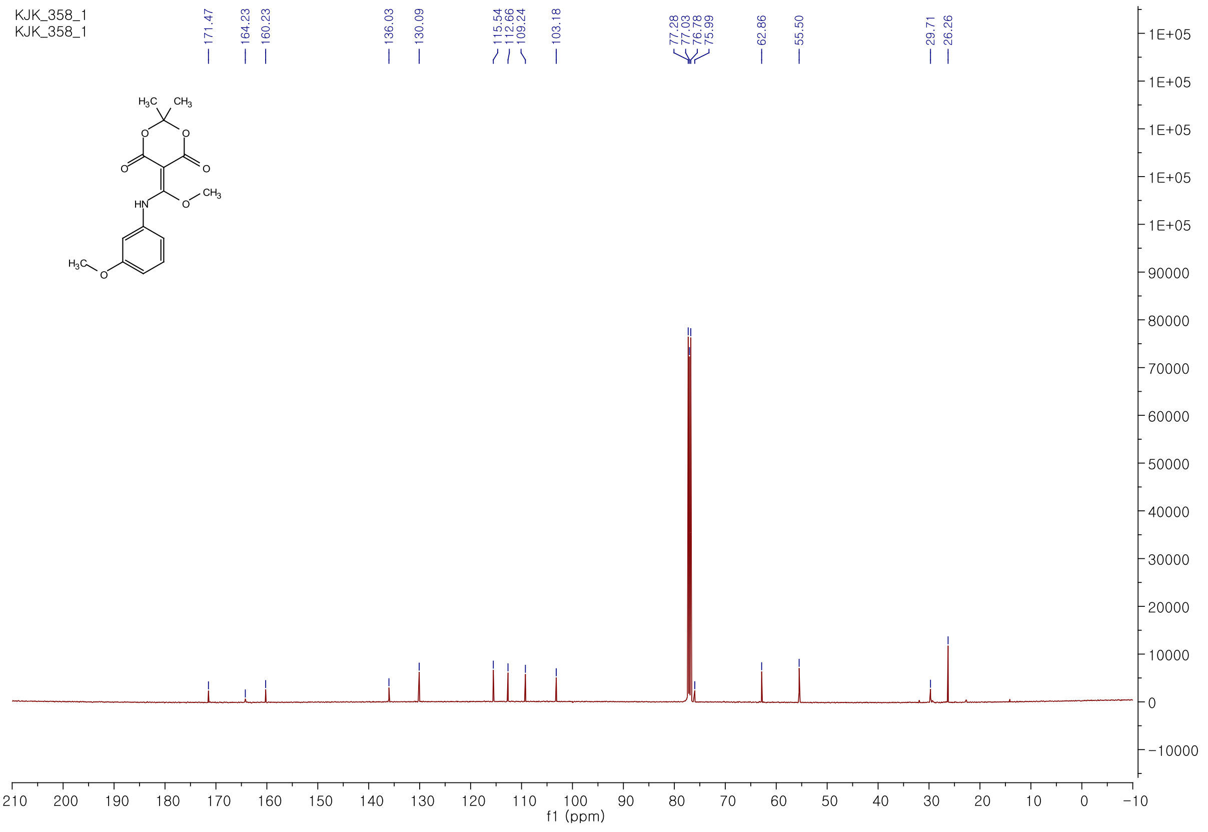

^{1}H NMR Studies of *N,S*-acetals **7an** and **7ao**

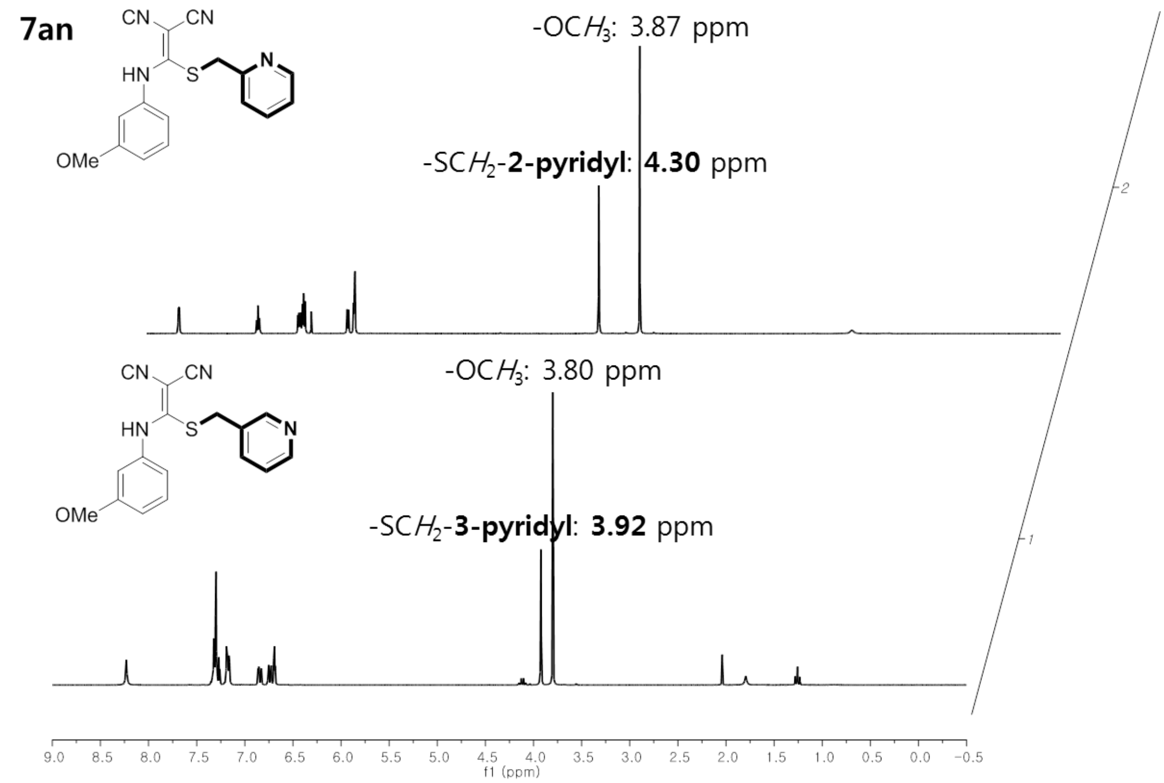

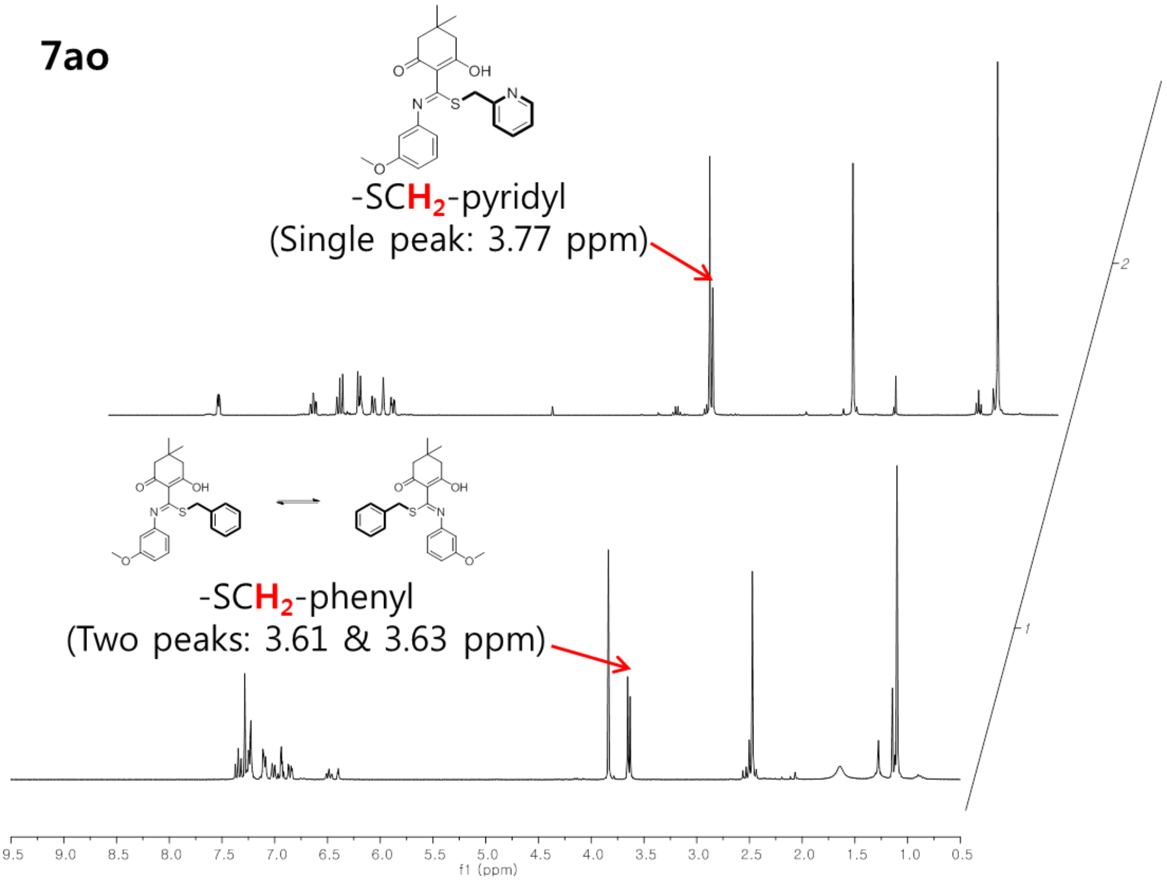

The time dependent ^{1}H NMR studies of the intramolecular aldol condensation of sulfur ylide-like intermediates **7aa** to **8aa** in *N,N*-dimethylformamide-d_7 at room temperature

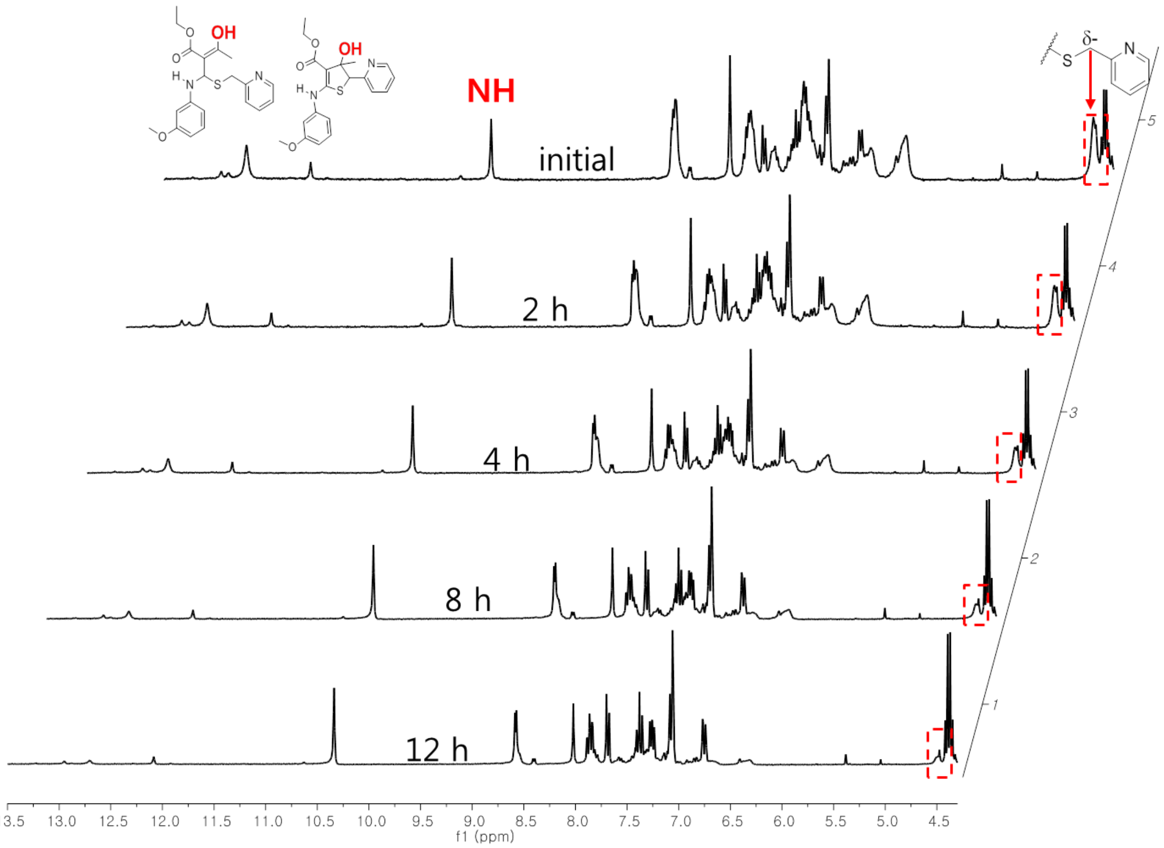

X-ray data of **8ad**

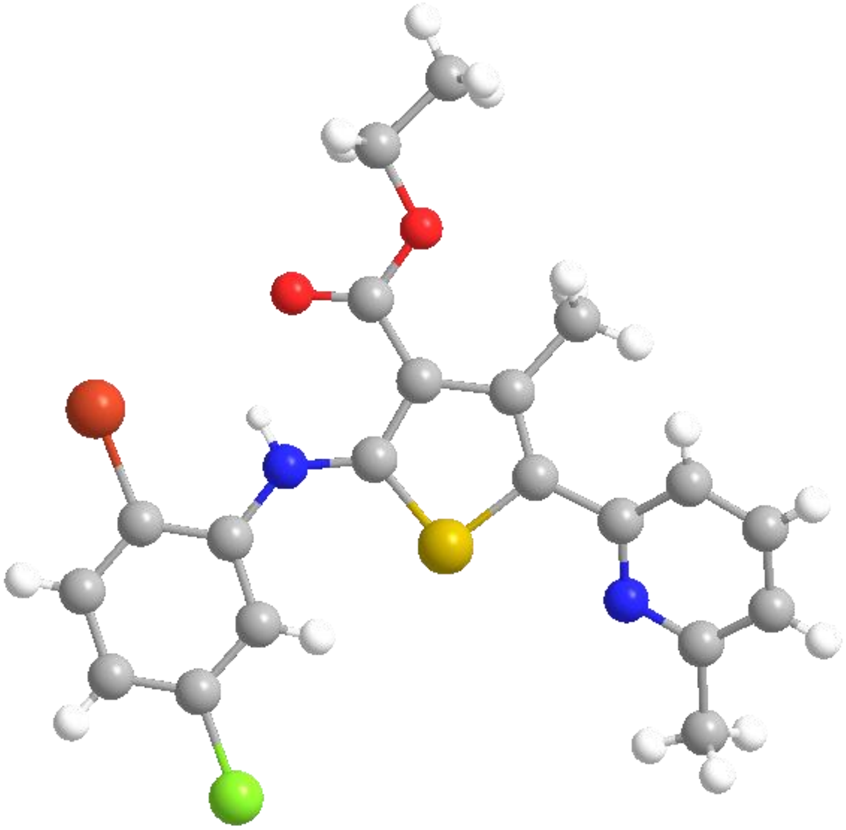

Table 1. Crystal data and structure refinement for SJP-IK0133

Identification code	20161114	
Empirical formula	C20 H18 Br Cl N2 O2 S	
Formula weight	465.78	
Temperature	296(1) K	
Wavelength	0.71073 Å	
Crystal system	Monoclinic	
Space group	P2(1)/c	
Unit cell dimensions	a = 7.7040(2) Å	α= 90°.
	b = 40.1958(9) Å	β= 111.536(2)°.
	c = 6.8191(2) Å	γ = 90°.
Volume	1964.24(9) Å^3	

Z	4
Density (calculated)	1.575 Mg/m^3
Absorption coefficient	2.353 mm^{-1}
F(000)	944
Crystal size	0.28 x 0.14 x 0.04 mm^3
Theta range for data collection	1.01 to 28.26°
Index ranges	0<=h<=10, -53<=k<=0, -9<=l<=8
Reflections collected	4788
Independent reflections	4788 [R(int) = 0.0000]
Completeness to theta = 28.26°	98.6 %
Absorption correction	Multi-scan
Max. and min. transmission	0.9118 and 0.5587
Refinement method	Full-matrix least-squares on F^2
Data / restraints / parameters	4788 / 0 / 244
Goodness-of-fit on F^2	1.063
Final R indices [I>2sigma(I)]	R1 = 0.0561, wR2 = 0.1245
R indices (all data)	R1 = 0.0902, wR2 = 0.1374
Largest diff. peak and hole	0.821 and -0.256 e.Å^{-3}

Table 2. Atomic coordinates (x 10^4) and equivalent isotropic displacement parameters (Å^{2}x 10^3) for SJP-IK0133. U(eq) is defined as one third of the trace of the orthogonalized U^{ij} tensor.

	x	y	z	U(eq)
Br(1)	2861(1)	2247(1)	8735(1)	55(1)
Cl(1)	7053(2)	898(1)	12587(2)	67(1)
S(1)	3024(2)	969(1)	4892(2)	48(1)
O(1)	613(5)	2014(1)	3449(5)	67(1)
O(2)	-665(4)	1836(1)	141(4)	52(1)
N(1)	2781(5)	1598(1)	6372(5)	46(1)
N(2)	2296(5)	322(1)	2787(6)	55(1)
C(1)	4048(6)	1838(1)	9817(6)	43(1)
C(2)	5091(6)	1822(1)	11939(6)	54(1)
C(3)	6036(6)	1532(1)	12816(7)	56(1)
C(4)	5891(6)	1264(1)	11538(6)	47(1)
C(5)	4834(6)	1275(1)	9397(6)	46(1)

C(6)	3878(5)	1565(1)	8492(6)	40(1)
C(7)	2298(5)	1378(1)	4711(6)	41(1)
C(8)	1144(5)	1456(1)	2657(6)	41(1)
C(9)	877(5)	1180(1)	1239(6)	39(1)
C(10)	1836(6)	905(1)	2210(6)	43(1)
C(11)	372(6)	1792(1)	2172(6)	44(1)
C(12)	-1550(6)	2157(1)	-431(7)	53(1)
C(13)	-2713(7)	2137(1)	-2751(8)	62(1)
C(14)	-409(6)	1184(1)	-1052(6)	53(1)
C(15)	2045(6)	571(1)	1422(7)	49(1)
C(16)	2088(7)	523(1)	-577(8)	65(1)
C(17)	2323(8)	208(2)	-1174(9)	82(2)
C(18)	2539(8)	-50(1)	223(11)	88(2)
C(19)	2539(6)	15(1)	2193(9)	67(1)
C(20)	2803(8)	-259(1)	3774(10)	97(2)

Table 3. Bond lengths [Å] and angles [°] for SJP-IK0133.

Br(1)-C(1)	1.893(4)
Cl(1)-C(4)	1.734(4)
S(1)-C(7)	1.729(4)
S(1)-C(10)	1.738(4)
O(1)-C(11)	1.215(4)
O(2)-C(11)	1.333(4)
O(2)-C(12)	1.443(5)
N(1)-C(7)	1.374(5)
N(1)-C(6)	1.387(5)
N(1)-H(1A)	0.8600
N(2)-C(19)	1.333(5)
N(2)-C(15)	1.334(5)
C(1)-C(2)	1.375(5)
C(1)-C(6)	1.399(5)
C(2)-C(3)	1.386(6)
C(2)-H(2B)	0.9300
C(3)-C(4)	1.365(6)

C(3)-H(3A)	0.9300
C(4)-C(5)	1.387(5)
C(5)-C(6)	1.397(5)
C(5)-H(5A)	0.9300
C(7)-C(8)	1.391(5)
C(8)-C(9)	1.436(5)
C(8)-C(11)	1.463(5)
C(9)-C(10)	1.359(5)
C(9)-C(14)	1.512(5)
C(10)-C(15)	1.476(5)
C(12)-C(13)	1.508(6)
C(12)-H(12A)	0.9700
C(12)-H(12B)	0.9700
C(13)-H(13A)	0.9600
C(13)-H(13B)	0.9600
C(13)-H(13C)	0.9600
C(14)-H(14A)	0.9600
C(14)-H(14B)	0.9600
C(14)-H(14C)	0.9600
C(15)-C(16)	1.389(6)
C(16)-C(17)	1.362(7)
C(16)-H(16A)	0.9300
C(17)-C(18)	1.378(8)
C(17)-H(17A)	0.9300
C(18)-C(19)	1.368(8)
C(18)-H(18A)	0.9300
C(19)-C(20)	1.502(7)
C(20)-H(20A)	0.9600
C(20)-H(20B)	0.9600
C(20)-H(20C)	0.9600
C(7)-S(1)-C(10)	91.92(17)
C(11)-O(2)-C(12)	116.5(3)
C(7)-N(1)-C(6)	132.5(3)
C(7)-N(1)-H(1A)	113.7
C(6)-N(1)-H(1A)	113.7
C(19)-N(2)-C(15)	119.0(4)

C(2)-C(1)-C(6)	121.5(4)
C(2)-C(1)-Br(1)	117.7(3)
C(6)-C(1)-Br(1)	120.8(3)
C(1)-C(2)-C(3)	120.4(4)
C(1)-C(2)-H(2B)	119.8
C(3)-C(2)-H(2B)	119.8
C(4)-C(3)-C(2)	118.7(4)
C(4)-C(3)-H(3A)	120.6
C(2)-C(3)-H(3A)	120.6
C(3)-C(4)-C(5)	121.8(4)
C(3)-C(4)-Cl(1)	119.8(3)
C(5)-C(4)-Cl(1)	118.4(3)
C(4)-C(5)-C(6)	120.1(4)
C(4)-C(5)-H(5A)	119.9
C(6)-C(5)-H(5A)	119.9
N(1)-C(6)-C(5)	124.1(3)
N(1)-C(6)-C(1)	118.4(3)
C(5)-C(6)-C(1)	117.5(3)
N(1)-C(7)-C(8)	124.5(3)
N(1)-C(7)-S(1)	124.7(3)
C(8)-C(7)-S(1)	110.8(3)
C(7)-C(8)-C(9)	112.7(3)
C(7)-C(8)-C(11)	119.4(3)
C(9)-C(8)-C(11)	127.9(3)
C(10)-C(9)-C(8)	112.5(3)
C(10)-C(9)-C(14)	123.0(3)
C(8)-C(9)-C(14)	124.4(3)
C(9)-C(10)-C(15)	132.4(4)
C(9)-C(10)-S(1)	112.0(3)
C(15)-C(10)-S(1)	115.6(3)
O(1)-C(11)-O(2)	121.3(3)
O(1)-C(11)-C(8)	125.1(3)
O(2)-C(11)-C(8)	113.5(3)
O(2)-C(12)-C(13)	105.9(3)
O(2)-C(12)-H(12A)	110.6
C(13)-C(12)-H(12A)	110.6
O(2)-C(12)-H(12B)	110.6

C(13)-C(12)-H(12B)	110.6
H(12A)-C(12)-H(12B)	108.7
C(12)-C(13)-H(13A)	109.5
C(12)-C(13)-H(13B)	109.5
H(13A)-C(13)-H(13B)	109.5
C(12)-C(13)-H(13C)	109.5
H(13A)-C(13)-H(13C)	109.5
H(13B)-C(13)-H(13C)	109.5
C(9)-C(14)-H(14A)	109.5
C(9)-C(14)-H(14B)	109.5
H(14A)-C(14)-H(14B)	109.5
C(9)-C(14)-H(14C)	109.5
H(14A)-C(14)-H(14C)	109.5
H(14B)-C(14)-H(14C)	109.5
N(2)-C(15)-C(16)	122.1(4)
N(2)-C(15)-C(10)	115.9(4)
C(16)-C(15)-C(10)	121.9(4)
C(17)-C(16)-C(15)	118.4(5)
C(17)-C(16)-H(16A)	120.8
C(15)-C(16)-H(16A)	120.8
C(16)-C(17)-C(18)	119.3(5)
C(16)-C(17)-H(17A)	120.3
C(18)-C(17)-H(17A)	120.3
C(19)-C(18)-C(17)	119.5(5)
C(19)-C(18)-H(18A)	120.3
C(17)-C(18)-H(18A)	120.3
N(2)-C(19)-C(18)	121.7(5)
N(2)-C(19)-C(20)	117.2(5)
C(18)-C(19)-C(20)	121.1(5)
C(19)-C(20)-H(20A)	109.5
C(19)-C(20)-H(20B)	109.5
H(20A)-C(20)-H(20B)	109.5
C(19)-C(20)-H(20C)	109.5
H(20A)-C(20)-H(20C)	109.5
H(20B)-C(20)-H(20C)	109.5

Symmetry transformations used to generate equivalent atoms:

Table 4. Anisotropic displacement parameters ($Å^2$x 10^3) for SJP-IK0133. The anisotropic displacement factor exponent takes the form: $-2\pi^2[h^2 a^{*2}U^{11} + ... + 2 h k a^* b^* U^{12}]$

	U^{11}	U^{22}	U^{33}	U^{23}	U^{13}	U^{12}
Br(1)	67(1)	44(1)	46(1)	-4(1)	12(1)	3(1)
Cl(1)	74(1)	60(1)	57(1)	14(1)	11(1)	13(1)
S(1)	62(1)	38(1)	38(1)	1(1)	13(1)	4(1)
O(1)	87(2)	43(2)	47(2)	-8(1)	-2(2)	11(2)
O(2)	66(2)	42(2)	37(1)	1(1)	7(1)	8(1)
N(1)	57(2)	36(2)	36(2)	-2(1)	6(2)	1(1)
N(2)	50(2)	40(2)	62(2)	-7(2)	6(2)	2(2)
C(1)	47(2)	41(2)	41(2)	1(2)	16(2)	-1(2)
C(2)	68(3)	50(2)	39(2)	-6(2)	16(2)	-2(2)
C(3)	63(3)	61(3)	37(2)	3(2)	10(2)	-1(2)
C(4)	46(2)	48(2)	44(2)	7(2)	13(2)	-1(2)
C(5)	52(2)	44(2)	39(2)	-1(2)	12(2)	-2(2)
C(6)	43(2)	42(2)	33(2)	1(2)	11(2)	-5(2)
C(7)	47(2)	38(2)	36(2)	-1(2)	12(2)	-3(2)
C(8)	46(2)	37(2)	38(2)	-2(2)	14(2)	-5(2)
C(9)	42(2)	41(2)	35(2)	-2(2)	15(2)	-5(2)
C(10)	50(2)	39(2)	40(2)	-6(2)	16(2)	-2(2)
C(11)	51(2)	39(2)	37(2)	0(2)	11(2)	-1(2)
C(12)	55(3)	44(2)	52(2)	2(2)	12(2)	5(2)
C(13)	58(3)	57(3)	60(3)	11(2)	8(2)	3(2)
C(14)	56(3)	51(2)	42(2)	-8(2)	6(2)	-5(2)
C(15)	49(2)	41(2)	53(2)	-9(2)	15(2)	-1(2)
C(16)	75(3)	61(3)	62(3)	-18(2)	30(3)	-3(2)
C(17)	80(4)	97(4)	75(4)	-37(3)	34(3)	4(3)
C(18)	76(4)	59(3)	112(5)	-36(3)	16(3)	10(3)
C(19)	54(3)	45(2)	79(3)	-16(2)	-3(2)	5(2)
C(20)	103(5)	44(3)	109(5)	-1(3)	-3(4)	8(3)

Table 5. Hydrogen coordinates (x 10^4) and isotropic displacement parameters ($Å^2$x 10^3) for SJP-IK0133.

	x	y	z	U(eq)
H(1A)	2313	1793	6031	55
H(2B)	5162	2006	12792	64
H(3A)	6755	1521	14248	67
H(5A)	4762	1088	8563	56
H(12A)	-620	2331	-163	63
H(12B)	-2331	2204	374	63
H(13A)	-3345	2344	-3217	93
H(13B)	-3616	1962	-2991	93
H(13C)	-1920	2091	-3524	93
H(14A)	-359	972	-1681	79
H(14B)	-22	1355	-1783	79
H(14C)	-1664	1227	-1147	79
H(16A)	1960	702	-1486	78
H(17A)	2338	168	-2511	99
H(18A)	2684	-267	-170	105
H(20A)	2762	-168	5057	146
H(20B)	3990	-364	4056	146
H(20C)	1826	-421	3220	146

Table 6. Torsion angles [°] for SJP-IK0133.

C(6)-C(1)-C(2)-C(3)	1.3(7)
Br(1)-C(1)-C(2)-C(3)	-178.2(3)
C(1)-C(2)-C(3)-C(4)	-0.8(7)
C(2)-C(3)-C(4)-C(5)	0.2(7)
C(2)-C(3)-C(4)-Cl(1)	-179.9(3)
C(3)-C(4)-C(5)-C(6)	-0.1(6)
Cl(1)-C(4)-C(5)-C(6)	180.0(3)
C(7)-N(1)-C(6)-C(5)	2.3(7)
C(7)-N(1)-C(6)-C(1)	-177.8(4)

C(4)-C(5)-C(6)-N(1)	-179.6(4)
C(4)-C(5)-C(6)-C(1)	0.5(6)
C(2)-C(1)-C(6)-N(1)	179.0(4)
Br(1)-C(1)-C(6)-N(1)	-1.5(5)
C(2)-C(1)-C(6)-C(5)	-1.1(6)
Br(1)-C(1)-C(6)-C(5)	178.4(3)
C(6)-N(1)-C(7)-C(8)	179.2(4)
C(6)-N(1)-C(7)-S(1)	-0.2(6)
C(10)-S(1)-C(7)-N(1)	-179.0(4)
C(10)-S(1)-C(7)-C(8)	1.5(3)
N(1)-C(7)-C(8)-C(9)	179.9(4)
S(1)-C(7)-C(8)-C(9)	-0.6(4)
N(1)-C(7)-C(8)-C(11)	0.3(6)
S(1)-C(7)-C(8)-C(11)	179.8(3)
C(7)-C(8)-C(9)-C(10)	-1.0(5)
C(11)-C(8)-C(9)-C(10)	178.6(4)
C(7)-C(8)-C(9)-C(14)	175.7(4)
C(11)-C(8)-C(9)-C(14)	-4.8(6)
C(8)-C(9)-C(10)-C(15)	-177.9(4)
C(14)-C(9)-C(10)-C(15)	5.4(7)
C(8)-C(9)-C(10)-S(1)	2.1(4)
C(14)-C(9)-C(10)-S(1)	-174.6(3)
C(7)-S(1)-C(10)-C(9)	-2.1(3)
C(7)-S(1)-C(10)-C(15)	177.9(3)
C(12)-O(2)-C(11)-O(1)	-2.9(6)
C(12)-O(2)-C(11)-C(8)	176.9(3)
C(7)-C(8)-C(11)-O(1)	-2.1(6)
C(9)-C(8)-C(11)-O(1)	178.3(4)
C(7)-C(8)-C(11)-O(2)	178.1(3)
C(9)-C(8)-C(11)-O(2)	-1.4(6)
C(11)-O(2)-C(12)-C(13)	-176.7(4)
C(19)-N(2)-C(15)-C(16)	-2.1(6)
C(19)-N(2)-C(15)-C(10)	-178.4(4)
C(9)-C(10)-C(15)-N(2)	-149.3(4)
S(1)-C(10)-C(15)-N(2)	30.7(5)
C(9)-C(10)-C(15)-C(16)	34.4(7)
S(1)-C(10)-C(15)-C(16)	-145.7(4)

N(2)-C(15)-C(16)-C(17)	2.6(7)
C(10)-C(15)-C(16)-C(17)	178.7(4)
C(15)-C(16)-C(17)-C(18)	-1.0(8)
C(16)-C(17)-C(18)-C(19)	-0.9(9)
C(15)-N(2)-C(19)-C(18)	0.1(7)
C(15)-N(2)-C(19)-C(20)	-179.6(4)
C(17)-C(18)-C(19)-N(2)	1.4(8)
C(17)-C(18)-C(19)-C(20)	-178.9(5)

Symmetry transformations used to generate equivalent atoms:

Table 7. Hydrogen bonds for SJP-IK0133 [Å and °].

D-H...A	d(D-H)	d(H...A)	d(D...A)	<(DHA)
N(1)-H(1A)...O(1)	0.86	1.98	2.670(4)	136.6
N(1)-H(1A)...Br(1)	0.86	2.52	3.056(3)	121.6

Symmetry transformations used to generate equivalent atoms:

X-ray data of **8an**

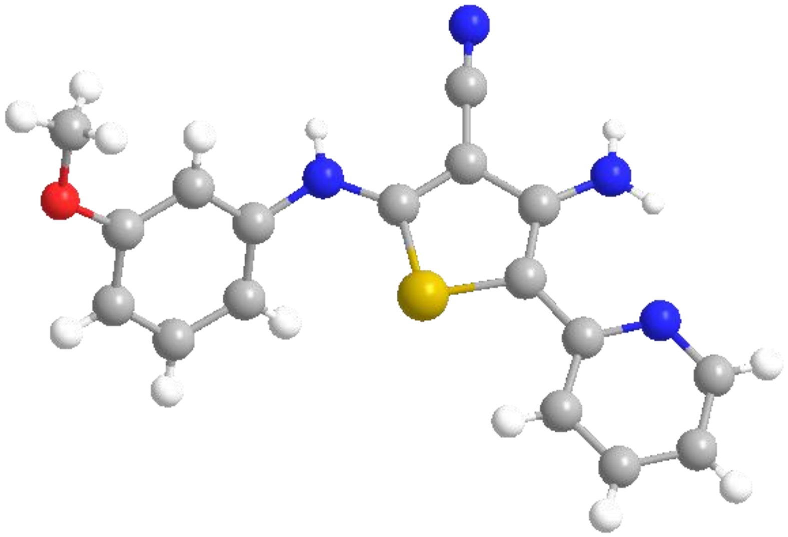

Table 1. Crystal data and structure refinement for KJK-164

Identification code	20170724_0m	
Empirical formula	C17 H14 N4 O S	
Formula weight	322.38	
Temperature	296(1) K	
Wavelength	0.71073 Å	
Crystal system	Triclinic	
Space group	P-1	
Unit cell dimensions	a = 7.8674(2) Å	α= 72.462(2)°.
	b = 9.2190(3) Å	β= 88.647(2)°.
	c = 11.3987(3) Å	γ = 81.174(2)°.
Volume	778.75(4) $Å^3$	
Z	2	
Density (calculated)	1.375 Mg/m^3	
Absorption coefficient	0.218 mm^{-1}	
F(000)	336	
Crystal size	0.30 x 0.16 x 0.08 mm^3	
Theta range for data collection	1.87 to 28.51°	

Index ranges	-10<=h<=10, -11<=k<=12, 0<=l<=15
Reflections collected	3931
Independent reflections	3931 [R(int) = 0.0000]
Completeness to theta = 28.51°	99.0 %
Absorption correction	Multi-scan
Max. and min. transmission	0.9828 and 0.9376
Refinement method	Full-matrix least-squares on F^2
Data / restraints / parameters	3931 / 0 / 208
Goodness-of-fit on F^2	1.062
Final R indices [I>2sigma(I)]	R1 = 0.0467, wR2 = 0.1178
R indices (all data)	R1 = 0.0564, wR2 = 0.1245
Largest diff. peak and hole	0.440 and -0.200 e.Å^{-3}

Table 2. Atomic coordinates (x 10^4) and equivalent isotropic displacement parameters (Å^{2}x 10^3) for KJK-164. U(eq) is defined as one third of the trace of the orthogonalized U^{ij} tensor.

	x	y	z	U(eq)
S(1)	3946(1)	2596(1)	358(1)	39(1)
O(1)	7662(2)	397(2)	5682(1)	61(1)
N(1)	7194(2)	1445(2)	1270(1)	42(1)
N(2)	9251(2)	124(2)	-1362(1)	52(1)
N(3)	4989(2)	1634(2)	-2720(1)	46(1)
N(4)	1571(2)	3007(2)	-2830(1)	45(1)
C(1)	8047(3)	-1219(2)	5873(2)	67(1)
C(2)	7226(2)	1343(2)	4516(1)	42(1)
C(3)	7416(2)	875(2)	3466(1)	39(1)
C(4)	6926(2)	1942(2)	2330(1)	37(1)
C(5)	6267(2)	3455(2)	2237(2)	43(1)
C(6)	6098(2)	3901(2)	3299(2)	47(1)
C(7)	6577(2)	2860(2)	4429(2)	46(1)
C(8)	6066(2)	1767(2)	318(1)	34(1)
C(9)	6379(2)	1412(2)	-770(1)	34(1)
C(10)	4902(2)	1826(2)	-1590(1)	33(1)
C(11)	3465(2)	2470(2)	-1098(1)	35(1)
C(12)	7987(2)	700(2)	-1075(1)	37(1)

C(13)	1784(2)	3093(2)	-1680(1)	37(1)
C(14)	51(2)	3625(2)	-3406(2)	55(1)
C(15)	-1302(2)	4352(2)	-2905(2)	62(1)
C(16)	-1104(2)	4405(2)	-1726(2)	62(1)
C(17)	435(2)	3764(2)	-1090(2)	50(1)

Table 3. Bond lengths [Å] and angles [°] for KJK-164.

S(1)-C(8)	1.7306(14)
S(1)-C(11)	1.7532(15)
O(1)-C(2)	1.3688(19)
O(1)-C(1)	1.424(2)
N(1)-C(8)	1.3502(19)
N(1)-C(4)	1.4171(18)
N(1)-H(1A)	0.8600
N(2)-C(12)	1.143(2)
N(3)-C(10)	1.3510(18)
N(3)-H(3A)	0.8600
N(3)-H(3B)	0.8600
N(4)-C(14)	1.337(2)
N(4)-C(13)	1.352(2)
C(1)-H(1B)	0.9600
C(1)-H(1C)	0.9600
C(1)-H(1D)	0.9600
C(2)-C(3)	1.387(2)
C(2)-C(7)	1.388(2)
C(3)-C(4)	1.390(2)
C(3)-H(3C)	0.9300
C(4)-C(5)	1.384(2)
C(5)-C(6)	1.389(2)
C(5)-H(5A)	0.9300
C(6)-C(7)	1.375(2)
C(6)-H(6A)	0.9300
C(7)-H(7A)	0.9300
C(8)-C(9)	1.3832(19)

C(9)-C(12)	1.4187(19)
C(9)-C(10)	1.439(2)
C(10)-C(11)	1.3816(19)
C(11)-C(13)	1.448(2)
C(13)-C(17)	1.406(2)
C(14)-C(15)	1.375(3)
C(14)-H(14A)	0.9300
C(15)-C(16)	1.373(3)
C(15)-H(15A)	0.9300
C(16)-C(17)	1.380(3)
C(16)-H(16A)	0.9300
C(17)-H(17A)	0.9300
C(8)-S(1)-C(11)	92.51(7)
C(2)-O(1)-C(1)	118.55(14)
C(8)-N(1)-C(4)	125.88(13)
C(8)-N(1)-H(1A)	117.1
C(4)-N(1)-H(1A)	117.1
C(10)-N(3)-H(3A)	120.0
C(10)-N(3)-H(3B)	120.0
H(3A)-N(3)-H(3B)	120.0
C(14)-N(4)-C(13)	117.93(15)
O(1)-C(1)-H(1B)	109.5
O(1)-C(1)-H(1C)	109.5
H(1B)-C(1)-H(1C)	109.5
O(1)-C(1)-H(1D)	109.5
H(1B)-C(1)-H(1D)	109.5
H(1C)-C(1)-H(1D)	109.5
O(1)-C(2)-C(3)	124.21(15)
O(1)-C(2)-C(7)	115.44(14)
C(3)-C(2)-C(7)	120.35(15)
C(2)-C(3)-C(4)	119.01(14)
C(2)-C(3)-H(3C)	120.5
C(4)-C(3)-H(3C)	120.5
C(5)-C(4)-C(3)	120.98(14)
C(5)-C(4)-N(1)	121.23(14)
C(3)-C(4)-N(1)	117.73(13)

C(4)-C(5)-C(6)	119.06(15)
C(4)-C(5)-H(5A)	120.5
C(6)-C(5)-H(5A)	120.5
C(7)-C(6)-C(5)	120.71(15)
C(7)-C(6)-H(6A)	119.6
C(5)-C(6)-H(6A)	119.6
C(6)-C(7)-C(2)	119.89(14)
C(6)-C(7)-H(7A)	120.1
C(2)-C(7)-H(7A)	120.1
N(1)-C(8)-C(9)	126.13(13)
N(1)-C(8)-S(1)	123.05(11)
C(9)-C(8)-S(1)	110.71(11)
C(8)-C(9)-C(12)	124.44(13)
C(8)-C(9)-C(10)	113.66(12)
C(12)-C(9)-C(10)	121.90(12)
N(3)-C(10)-C(11)	125.84(13)
N(3)-C(10)-C(9)	121.90(13)
C(11)-C(10)-C(9)	112.23(12)
C(10)-C(11)-C(13)	127.66(13)
C(10)-C(11)-S(1)	110.88(11)
C(13)-C(11)-S(1)	121.30(11)
N(2)-C(12)-C(9)	177.26(16)
N(4)-C(13)-C(17)	121.21(14)
N(4)-C(13)-C(11)	116.70(13)
C(17)-C(13)-C(11)	122.09(15)
N(4)-C(14)-C(15)	124.06(18)
N(4)-C(14)-H(14A)	118.0
C(15)-C(14)-H(14A)	118.0
C(16)-C(15)-C(14)	118.10(17)
C(16)-C(15)-H(15A)	120.9
C(14)-C(15)-H(15A)	120.9
C(15)-C(16)-C(17)	119.80(17)
C(15)-C(16)-H(16A)	120.1
C(17)-C(16)-H(16A)	120.1
C(16)-C(17)-C(13)	118.82(17)
C(16)-C(17)-H(17A)	120.6
C(13)-C(17)-H(17A)	120.6

Symmetry transformations used to generate equivalent atoms:

Table 4. Anisotropic displacement parameters ($Å^2 x 10^3$) for KJK-164. The anisotropic displacement factor exponent takes the form: $-2\pi^2[h^2 a^{*2}U^{11} + ... + 2 h k a^* b^* U^{12}]$

	U^{11}	U^{22}	U^{33}	U^{23}	U^{13}	U^{12}
S(1)	35(1)	46(1)	36(1)	-19(1)	2(1)	3(1)
O(1)	84(1)	60(1)	34(1)	-15(1)	-4(1)	10(1)
N(1)	37(1)	56(1)	34(1)	-21(1)	-2(1)	7(1)
N(2)	39(1)	68(1)	47(1)	-25(1)	3(1)	6(1)
N(3)	41(1)	66(1)	33(1)	-22(1)	1(1)	2(1)
N(4)	40(1)	51(1)	41(1)	-15(1)	-5(1)	0(1)
C(1)	82(2)	56(1)	52(1)	-7(1)	-5(1)	3(1)
C(2)	43(1)	51(1)	33(1)	-15(1)	0(1)	-3(1)
C(3)	38(1)	43(1)	38(1)	-17(1)	-1(1)	0(1)
C(4)	34(1)	46(1)	34(1)	-18(1)	2(1)	-5(1)
C(5)	49(1)	41(1)	39(1)	-11(1)	0(1)	-6(1)
C(6)	54(1)	40(1)	50(1)	-20(1)	5(1)	-7(1)
C(7)	53(1)	52(1)	40(1)	-23(1)	6(1)	-8(1)
C(8)	33(1)	36(1)	33(1)	-11(1)	3(1)	-2(1)
C(9)	32(1)	37(1)	31(1)	-12(1)	4(1)	-1(1)
C(10)	36(1)	34(1)	30(1)	-10(1)	4(1)	-3(1)
C(11)	36(1)	37(1)	33(1)	-13(1)	1(1)	-1(1)
C(12)	35(1)	45(1)	32(1)	-14(1)	2(1)	-2(1)
C(13)	36(1)	34(1)	42(1)	-14(1)	-1(1)	-3(1)
C(14)	50(1)	62(1)	52(1)	-18(1)	-13(1)	1(1)
C(15)	44(1)	60(1)	79(1)	-25(1)	-20(1)	8(1)
C(16)	39(1)	62(1)	92(2)	-41(1)	-4(1)	10(1)
C(17)	41(1)	55(1)	59(1)	-30(1)	-2(1)	2(1)

Table 5. Hydrogen coordinates (x 10^4) and isotropic displacement parameters (Å^{2}x 10 ^{3}) for KJK-164.

	x	y	z	U(eq)
H(1A)	8163	888	1229	51
H(3A)	4096	1923	-3203	56
H(3B)	5937	1221	-2957	56
H(1B)	8335	-1735	6726	101
H(1C)	9004	-1432	5380	101
H(1D)	7062	-1580	5642	101
H(3C)	7865	-138	3521	47
H(5A)	5942	4162	1473	52
H(6A)	5656	4915	3245	56
H(7A)	6467	3173	5134	55
H(14A)	-100	3559	-4194	66
H(15A)	-2322	4796	-3353	74
H(16A)	-2004	4871	-1357	75
H(17A)	578	3775	-285	60

Table 6. Torsion angles [°] for KJK-164.

C(1)-O(1)-C(2)-C(3)	-11.7(3)
C(1)-O(1)-C(2)-C(7)	169.12(17)
O(1)-C(2)-C(3)-C(4)	180.00(15)
C(7)-C(2)-C(3)-C(4)	-0.9(2)
C(2)-C(3)-C(4)-C(5)	0.4(2)
C(2)-C(3)-C(4)-N(1)	177.75(14)
C(8)-N(1)-C(4)-C(5)	-46.3(2)
C(8)-N(1)-C(4)-C(3)	136.40(16)
C(3)-C(4)-C(5)-C(6)	0.0(2)
N(1)-C(4)-C(5)-C(6)	-177.22(15)
C(4)-C(5)-C(6)-C(7)	0.0(3)
C(5)-C(6)-C(7)-C(2)	-0.5(3)

O(1)-C(2)-C(7)-C(6)	-179.88(16)
C(3)-C(2)-C(7)-C(6)	0.9(3)
C(4)-N(1)-C(8)-C(9)	173.36(15)
C(4)-N(1)-C(8)-S(1)	-10.9(2)
C(11)-S(1)-C(8)-N(1)	-176.38(13)
C(11)-S(1)-C(8)-C(9)	-0.02(12)
N(1)-C(8)-C(9)-C(12)	-2.8(2)
S(1)-C(8)-C(9)-C(12)	-179.01(12)
N(1)-C(8)-C(9)-C(10)	176.86(14)
S(1)-C(8)-C(9)-C(10)	0.64(16)
C(8)-C(9)-C(10)-N(3)	176.78(13)
C(12)-C(9)-C(10)-N(3)	-3.6(2)
C(8)-C(9)-C(10)-C(11)	-1.13(18)
C(12)-C(9)-C(10)-C(11)	178.54(14)
N(3)-C(10)-C(11)-C(13)	-1.3(3)
C(9)-C(10)-C(11)-C(13)	176.52(14)
N(3)-C(10)-C(11)-S(1)	-176.74(12)
C(9)-C(10)-C(11)-S(1)	1.07(16)
C(8)-S(1)-C(11)-C(10)	-0.62(12)
C(8)-S(1)-C(11)-C(13)	-176.40(13)
C(8)-C(9)-C(12)-N(2)	175(4)
C(10)-C(9)-C(12)-N(2)	-5(4)
C(14)-N(4)-C(13)-C(17)	2.1(2)
C(14)-N(4)-C(13)-C(11)	-177.58(15)
C(10)-C(11)-C(13)-N(4)	1.7(2)
S(1)-C(11)-C(13)-N(4)	176.72(11)
C(10)-C(11)-C(13)-C(17)	-177.96(15)
S(1)-C(11)-C(13)-C(17)	-2.9(2)
C(13)-N(4)-C(14)-C(15)	0.6(3)
N(4)-C(14)-C(15)-C(16)	-2.3(3)
C(14)-C(15)-C(16)-C(17)	1.3(3)
C(15)-C(16)-C(17)-C(13)	1.2(3)
N(4)-C(13)-C(17)-C(16)	-3.0(3)
C(11)-C(13)-C(17)-C(16)	176.69(17)

Symmetry transformations used to generate equivalent atoms:

Table 7. Hydrogen bonds for KJK-164 [Å and °].

D-H...A	d(D-H)	d(H...A)	d(D...A)	<(DHA)
N(1)-H(1A)...N(2)#1	0.86	2.09	2.9335(19)	165.6
N(3)-H(3A)...N(4)	0.86	2.18	2.780(2)	127.0
N(3)-H(3B)...O(1)#2	0.86	2.26	3.0623(19)	154.6

Symmetry transformations used to generate equivalent atoms:

#1 -x+2,-y,-z #2 x,y,z-1
